# Inferior Olive HCN1 Channels Coordinate Synaptic Integration and Complex Spike Timing

**DOI:** 10.1016/j.celrep.2018.01.069

**Published:** 2018-02-13

**Authors:** Derek L.F. Garden, Marlies Oostland, Marta Jelitai, Arianna Rinaldi, Ian Duguid, Matthew F. Nolan

**Affiliations:** 1 Centre for Discovery Brain Sciences, University of Edinburgh, Edinburgh EH8 9XD, UK; 2 Simons Initiative for the Developing Brain, University of Edinburgh, Edinburgh EH8 9XD, UK

**Keywords:** excitability, synaptic integration, oscillation, gap junction, inferior olive, cerebellum, movement coordination, neural code, hyperpolarization-activated current, ion channel

## Abstract

Cerebellar climbing-fiber-mediated complex spikes originate from neurons in the inferior olive (IO), are critical for motor coordination, and are central to theories of cerebellar learning. Hyperpolarization-activated cyclic-nucleotide-gated (HCN) channels expressed by IO neurons have been considered as pacemaker currents important for oscillatory and resonant dynamics. Here, we demonstrate that *in vitro*, network actions of HCN1 channels enable bidirectional glutamatergic synaptic responses, while local actions of HCN1 channels determine the timing and waveform of synaptically driven action potentials. These roles are distinct from, and may complement, proposed pacemaker functions of HCN channels. We find that in behaving animals HCN1 channels reduce variability in the timing of cerebellar complex spikes, which serve as a readout of IO spiking. Our results suggest that spatially distributed actions of HCN1 channels enable the IO to implement network-wide rules for synaptic integration that modulate the timing of cerebellar climbing fiber signals.

## Highlights

• HCN1 channels in IO neurons control synaptic response and spiking activity
• Network actions of HCN1 channels enable bidirectional synaptic responses
• Local actions of HCN1 channels control spike timing and spikelet number
• In awake mice, HCN1 channels reduce timing variability of cerebellar complex spikes

## Graphical Abstract

**Figure:**
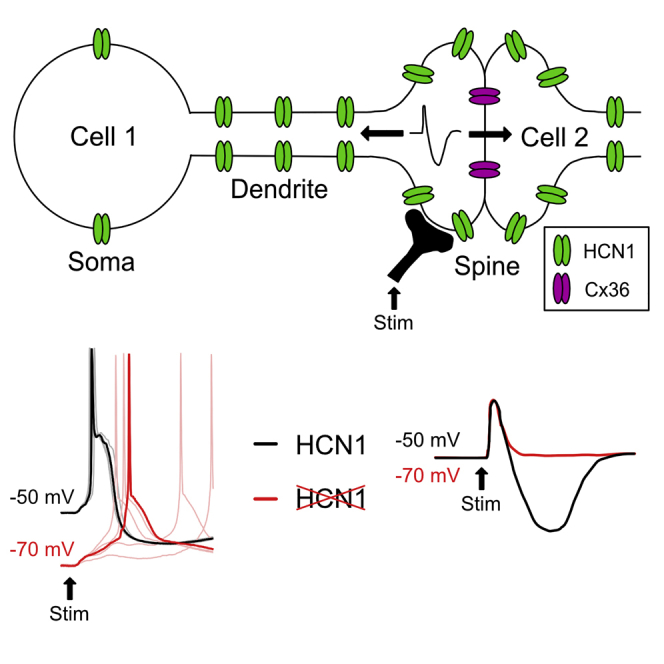

## Teaser

Garden et al. show that in the IO, distinct network-wide and local actions of HCN1 channels, respectively, determine the waveform of synaptic potentials and timing of spike initiation. Thus, spatially distributed actions of HCN1 signaling may support roles of the IO in motor coordination.

## Introduction

Theories of motor learning propose critical roles for the timing of cerebellar complex spikes, which originate from neurons in the inferior olive (IO) (Albus, 1970, De Zeeuw et al., 2011, Marr, 1969). This is supported by evidence that the frequency and timing of IO action potentials instructs the amplitude and direction of synaptic plasticity in the cerebellar cortex (Mathy et al., 2009). Thus, mechanisms that control the timing of spike output from the IO may play key roles in cerebellar-dependent motor coordination and learning.

Spike timing emerges from dynamic interactions between synaptic activity and intrinsic neuronal excitability (Dayan and Abbott, 2001). Neurons in the IO are striking in that intrinsic excitability appears to have a powerful influence on these dynamics. Spontaneous sinusoidal subthreshold membrane potential oscillations and membrane potential resonance emerge from interactions between multiple ion channel types (Benardo and Foster, 1986, Llinás and Yarom, 1981a, Llinás and Yarom, 1981b, Matsumoto-Makidono et al., 2016). Excitability of neurons in the IO also influences integration of synaptic inputs, with glutamatergic inputs to neurons in the IO generating distinct bidirectional synaptic potentials through recruitment of calcium-activated potassium channels (Garden et al., 2017). Gap-junction-mediated electrical synaptic connections between IO neurons synchronize oscillatory activity (Bal and McCormick, 1997, De Zeeuw et al., 1998, Llinas et al., 1974, Long et al., 2002) and have been proposed also to coordinate synaptic integration (Kistler and De Zeeuw, 2005). These distinctive excitable properties have motivated suggestions that the IO has unique computational roles within the brain (De Zeeuw et al., 1998, Welsh and Llinás, 1997). Nevertheless, the extent to which intrinsic excitability of IO neurons influences spike timing in behaving animals is unclear.

Excitability is determined at a molecular level by the set of ion channels that a neuron expresses. Of particular interest are the hyperpolarization-activated cyclic-nucleotide-gated (HCN) family of ion channels, which mediate hyperpolarization-activated currents (*I*_h_) that contribute to pacemaking and integrative properties of many central neurons (Robinson and Siegelbaum, 2003). HCN channels in the IO are suggested to act as pacemakers of oscillatory activity and mediate membrane potential resonance (Bal and McCormick, 1997, Matsumoto-Makidono et al., 2016). In contrast, their impact on synaptic integration in the IO is unclear. The HCN1 subunit is highly expressed in the IO and cerebellar cortex (Notomi and Shigemoto, 2004, Santoro et al., 2000). Global deletion of HCN1 channels causes deficits in learned motor behaviors (Nolan et al., 2003). While impairments in later stages of motor learning can in part be accounted for by contributions of HCN1 channels to synaptic integration in cerebellar Purkinje cells (Rinaldi et al., 2013), it is not clear whether HCN channels in upstream neurons influence activity in the cerebellar cortex. Here, we asked whether HCN1 channels in the IO affect synaptic integration as well as membrane potential resonance and oscillations and whether the contribution of HCN1 channels to excitability in the IO affects action potential firing in behaving animals.

We demonstrate that HCN1 channels mediate *I*_h_ in IO neurons, are required for the inhibitory component of responses to glutamatergic synaptic inputs, and oppose temporal summation of subthreshold inputs while also controlling the timing and waveform of spike output. Whereas the suprathreshold actions of HCN1 rely on local depolarization of the somatic membrane potential, generation of inhibitory components of synaptic responses involves network actions of HCN1 mediated by electrical connections with other IO neurons. We find that genetic deletion of HCN1 increases variability in the timing of complex spikes recorded from cerebellar Purkinje cells during quiet wakefulness and movement. Thus, HCN1 channels within the IO have multiple spatially distributed actions that may impact motor coordination by influencing the pattern of cerebellar complex spike activity.

## Results

### Pharmacological Block of *I*_h_ Modifies Excitability and Synaptic Integration

**Figure 1:**
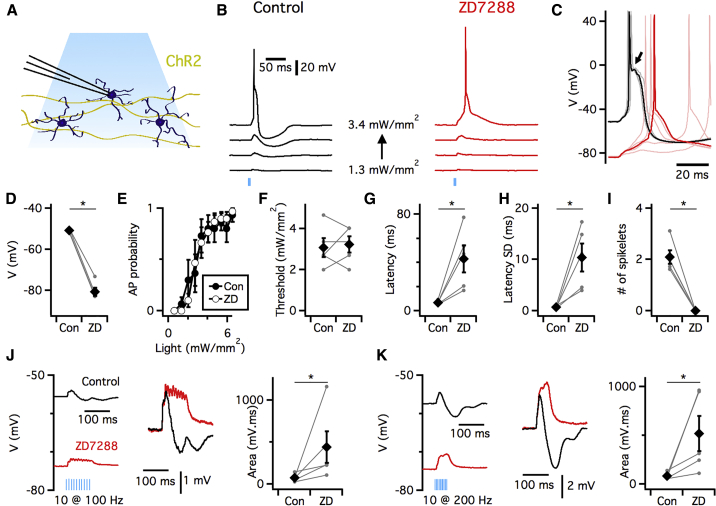
Temporal Integration by IO Neurons Is Modified by Block of HCN Channels (A) Whole-cell recordings were made from neurons in the IO. ChR2 was expressed in axons that project into the IO, but not in IO neurons. (B) Example responses to increasing intensity of light stimulation (vertical bar, 1.3–3.4 mW/mm^2^) of IO neurons from Thy1-ChR2 mice before (left) and during (right) application of 10 μM ZD7288. In the presence of ZD7288, the excitatory component of the subthreshold response is maintained (control: 2.63 ± 0.65 mV, ZD7288: 1.52 ± 0.60 mV, p = 0.081, n = 5, paired t test), but the hyperpolarizing component is abolished (control: −2.72 ± 0.95 mV, ZD7288: −0.04 ± 0.02 mV, p = 0.049, n = 5, paired t test). (C) Suprathreshold responses from (B) on an expanded timescale. Solid trace shows response with median latency, while lighter traces show additional responses to the same intensity of stimulation for each condition. Arrow indicates spikelets. (D) Resting membrane potential is more negative during (ZD) compared with before (Con) perfusion of 10 μM ZD7288 (p = 5.08 × 10^−5^, n = 5, paired t test). Individual data points are shown as filled circles and mean values as black diamonds. (E) Perfusion of ZD7288 does not affect the probability of spike firing as a function of stimulus intensity (F_1,70_ = 0.004, p = 0.96, two-way repeated-measures ANOVA, n = 5). (F–I) Comparison before (Con) and during application of ZD7288 (ZD) of the mean light threshold for AP firing (p = 0.82, n = 5, paired t test) (F), the spike latency at the threshold stimulus intensity (p = 0.01, n = 5, paired t test) (G), the SD of the spike latency at the threshold stimulus intensity (p = 0.02, n = 5, paired t test) (H), and the number of spikelets (p = 0.0014, n = 5, paired t test) (I). (J and K) Example responses to trains of 10 stimuli at 100 Hz (J) and 200 Hz (K) before and during application of ZD7288 (left). ZD7288 increases the area of the depolarization (100 Hz: p = 0.02, 200 Hz: p = 5.14 × 10^−3^, paired t test). Error bars in (D)–(K) indicate SEM.

To investigate whether *I*_h_ influences excitability and synaptic integration by IO neurons, we first examined actions of the HCN channel blocker ZD7288. We made patch-clamp recordings in brain slices from IO principal neurons identified by their large soma and characteristic action potential after depolarization (Llinás and Yarom, 1981b). To investigate synaptic responses, we used mice that express channelrhodopsin 2 (ChR2) and enhanced yellow fluorescent protein (eYFP) under control of the Thy1 promoter (Arenkiel et al., 2007). In these mice, activation of ChR2 with low intensities of 480-nm light reliably evoked bidirectional glutamatergic postsynaptic potentials (PSPs) in IO neurons (Garden et al., 2017) (Figures 1A and 1B). At higher intensities, synaptic excitation triggered action potentials and associated spikelets (Figures 1B and 1C).

We found that perfusion of ZD7288 hyperpolarized IO neurons by ∼30 mV (p = 5.08 × 10^−5^, n = 5, paired t test) (Figure 1C and 1D). The inhibitory component of the subthreshold PSP was abolished by ZD7288 (∼1% of control amplitude, p = 0.049, paired t test), while the excitatory component was maintained (**∼** 55% of control amplitude, p = 0.081, paired t test) (Figure 1B). Surprisingly, given the large change in membrane potential, block of *I*_h_ did not affect the relationship between stimulus intensity and spike probability (F_1,70_ = 0.004 p = 0.96, two-way repeated-measures ANOVA) (Figure 1E) or the threshold light intensity required to trigger a spike (p = 0.82, paired t test) (Figure 1F). However, the mean and SD of the latency of the spike response were increased more than 5-fold by ZD7288 (p = 0.01 and p = 0.02 respectively, paired t test) (Figures 1C, 1G, and 1H), while spikelets associated with the action potentials were abolished (p = 0.0014, paired t test) (Figures 1C and 1I).

We also examined subthreshold temporal summation in response to trains of synaptic input activated at 100 and 200 Hz (Figures 1J and 1K). In control conditions, trains at either stimulation frequency caused an initial rapid depolarization that reached a maximal amplitude within ∼ 16 ms of stimulation, followed by a hyperpolarization that peaked within ∼70 ms of stimulation (see also Garden et al., 2017, Turecek et al., 2014). Following block of *I*_h_ with ZD7288 the hyperpolarizing response was abolished, while the amplitude and duration of the depolarizing component were increased. These changes were also reflected in a large increase in the area of the synaptic responses (100 Hz: p = 0.02, n = 5; 200 Hz, p = 5.14 × 10^−3^, n = 4, paired t test).

Together, these data indicate that pharmacological block of *I*_h_ increases the latency and variability in the timing of synaptically driven action potentials, prevents associated spikelets, abolishes inhibitory components of subthreshold synaptic inputs, and increases temporal summation of subthreshold responses. Thus, *I*_h_ may be a major determinant of the way IO neurons respond to synaptic input, with roles that appear distinct from its known functions in other neuron types.

### HCN1 Channels Enable the Hyperpolarizing Component of Long-Range Synaptic Responses

Do HCN1 channels in IO neurons mediate the integrative roles of *I*_h_ suggested by pharmacological manipulation? To address this, we used mice in which the *HCN1* gene is deleted (Nolan et al., 2003). Comparison of HCN1 knockout (*HCN1*^−/−^) mice with control (*HCN1*^*+/+*^) mice demonstrated that HCN1 channels mediate the prominent *I*_h_ recorded from IO neurons and that deletion of HCN1 causes similar changes in resting properties of IO neurons to pharmacological block of *I*_h_ (Figure S1). These data are largely consistent with and extend recent observations of changes in membrane currents and integrative properties of IO neurons in *HCN1*^−/−^ mice (Matsumoto-Makidono et al., 2016). We therefore went on to address the influence of HCN1 channels on subthreshold PSPs by comparing responses to excitatory synaptic input of IO neurons from *HCN1*^*+/+*^ and *HCN1*^−/−^ mice.

**Figure 2:**
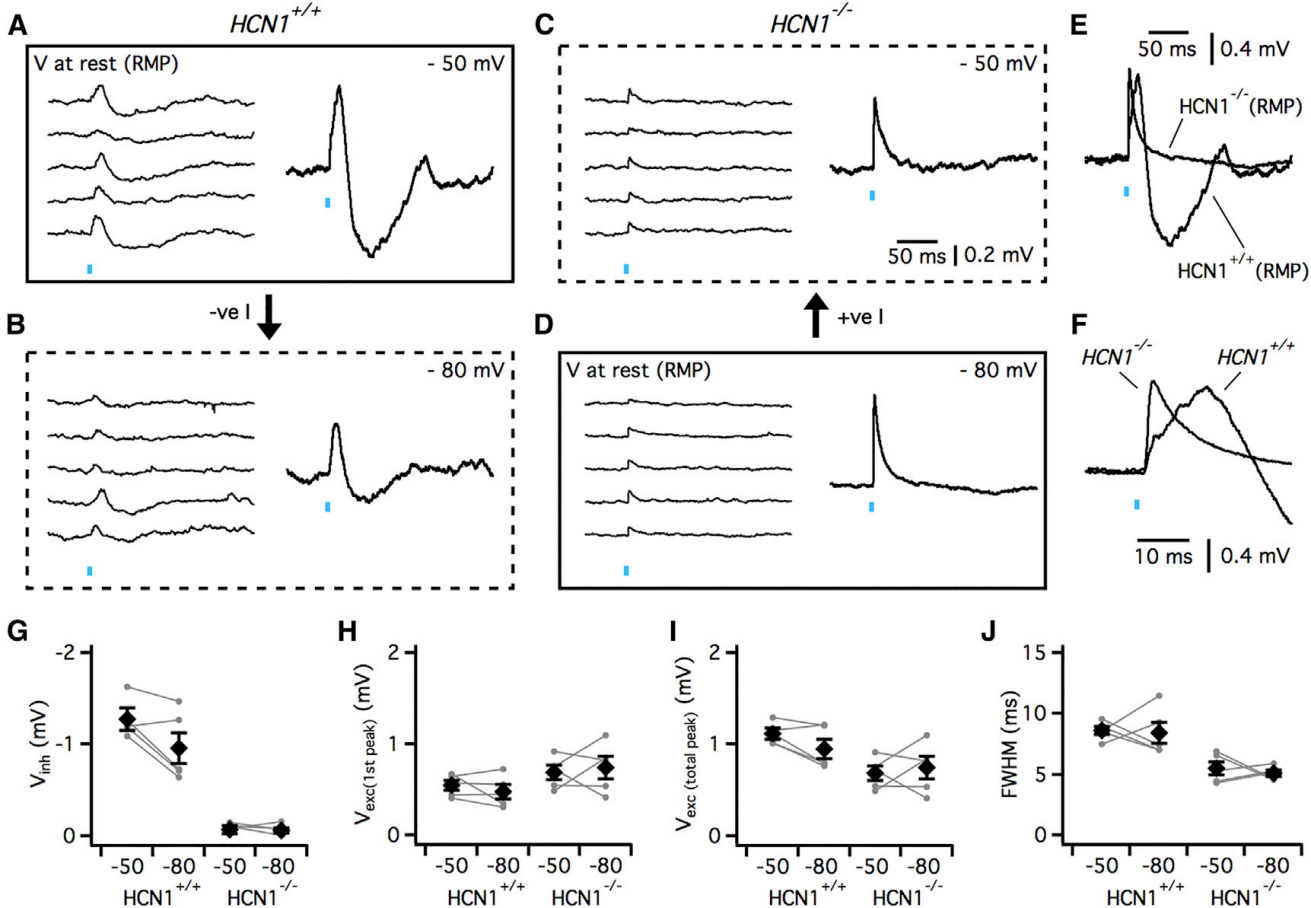
HCN1 Channels Control the Waveform of Responses to Long-Range Synaptic Input (A–D) Example responses of IO neurons from *HCN*^*+/+*^ (A and B) and *HCN*^−/−^ mice (C and D) to optical activation of neocortical inputs recorded with membrane potential at −50 mV (A and C) or −80 mV (B and D). The same stimulus is repeated 5 times. Mean responses are shown to the right of individual traces. Boxes constructed from solid lines indicate responses recorded at the resting potential and arrows indicate direction of injected current. (E and F) Responses from (A) and (D) on a faster timescale illustrate differences at the resting membrane potential of the inhibitory (E) and excitatory (F) components of PSPs. (G–J) Comparison between *HCN*^*+/+*^ and *HCN*^*−/−*^ mice of the amplitude of the inhibitory component (genotype: F_1,16_ = 110.8 p = 1.34 × 10^−8^; membrane potential: F_1,16_ = 2.50 p = 0.13) (G), the amplitude of the first peak of the excitatory component (genotype: F_1,16_ = 5.64 p = 0.03; membrane potential: F_1,16_ = 0.006 p = 0.94) (H), the maximal amplitude of the excitatory component (genotype: F_1,16_ = 12.2 p = 0.003; membrane potential: p = 0.57) (I), and the width of the excitatory component at its half maximum amplitude (FWHM) (genotype: F_1,16_ = 34.7 p = 2.29 × 10^−5^; membrane potential: F_1,16_ = 0.26 p = 0.62) (J). All comparisons use a two-way ANOVA (n = 5). Individual data points are shown as filled circles and mean values as diamonds. Error bars indicate SEM.

To evaluate responses to glutamatergic synaptic inputs in *HCN1*^*+/+*^ and *HCN1*^−/−^ mice, we used slices from mice in which adeno-associated virus (AAV) expressing ChR2 was injected into the motor cortex (Garden et al., 2017). In these experiments, we first compared responses of each genotype when neurons were at their resting membrane potential (Figures 2A and 2D–2F). Because deletion or block of HCN1 channels causes a large hyperpolarization of the resting membrane potential of IO neurons, and because this might be expected to modify the driving force for synaptic currents and gating of other voltage-gated ion channels, we also investigated whether the waveform of synaptic responses differed when compared at similar membrane potentials (Figures 2B and 2C). Thus, neurons from *HCN1*^*+/+*^ mice were hyperpolarized to −80 mV by injection of negative current (Figure 2B), whereas neurons from *HCN1*^−/−^ mice were depolarized to −50 mV by injection of positive current (Figure 2C).

Whereas in IO neurons from HCN1^+/+^ mice the inhibitory components of the response to neocortical input was observed at resting potential and at −80 mV (Figures 2A, 2B, 2E, and 2G), it was completely absent at both test potentials in IO neurons from HCN1^−/−^ mice (F_1,16_ = 110.8, p = 1.34 × 10^−8^ for effect of genotype, ANOVA, n = 5) (Figures 2C–2E and 2G). In addition, while initial excitatory responses were present in neurons from both groups of mice, their waveform differed. Excitatory responses from HCN1^+/+^ mice had two or more peaks, whereas for IO neurons from HCN1^−/−^ mice, they had only a single peak (Figure 2F). In the absence of HCN1, the amplitude of the first peak was slightly larger (F_1,16_ = 5.6, p = 0.03, ANOVA), while the maximum amplitude was reduced (F_1,16_ = 12.2, p = 0.003, ANOVA) and the overall duration of the excitatory component was shorter (F_1,16_ = 34.7 p = 2.29 × 10^−5^, ANOVA) (Figure 2G–2J). Changing the membrane potential between −50 mV and −80 mV had relatively little effect on the amplitude of the excitatory (F_1,16_ = 0.006, p = 0.94 for effect of membrane potential on the first peak and F_1,16_ = 0.34, p = 0.57 for maximum amplitude, ANOVA, n = 5) or the inhibitory component of the PSP (F_1,16_ = 2.5, p = 0.13). We obtained similar results using the blocker ZD7288, indicating that the dependence of inhibitory potentials on HCN1 channels is a direct result of the absence of *HCN1* rather than a secondary adaptation following gene deletion (Figure S2).

Together, these data indicate that direct activation of HCN1 channels is required for inhibitory components of IO responses to long-range glutamatergic inputs and controls the waveform of excitatory components. Because hyperpolarization of the resting potential of neurons from *HCN1*^*+/+*^ mice did not replicate, and depolarization of neurons from *HCN1*^−/−^ mice did not rescue, the effects of *HCN1* deletion (cf. Figures 2A–2D and 2G–2J), the requirement of HCN1 channels for the inhibitory component of the GluA synaptic responses of IO neurons is independent of their actions on the somatic membrane potential.

### HCN1 Channels in Adjoining Electrically Coupled IO Neurons Are Sufficient for Generation of the Inhibitory Component of Glutamatergic Synaptic Responses

**Figure 3:**
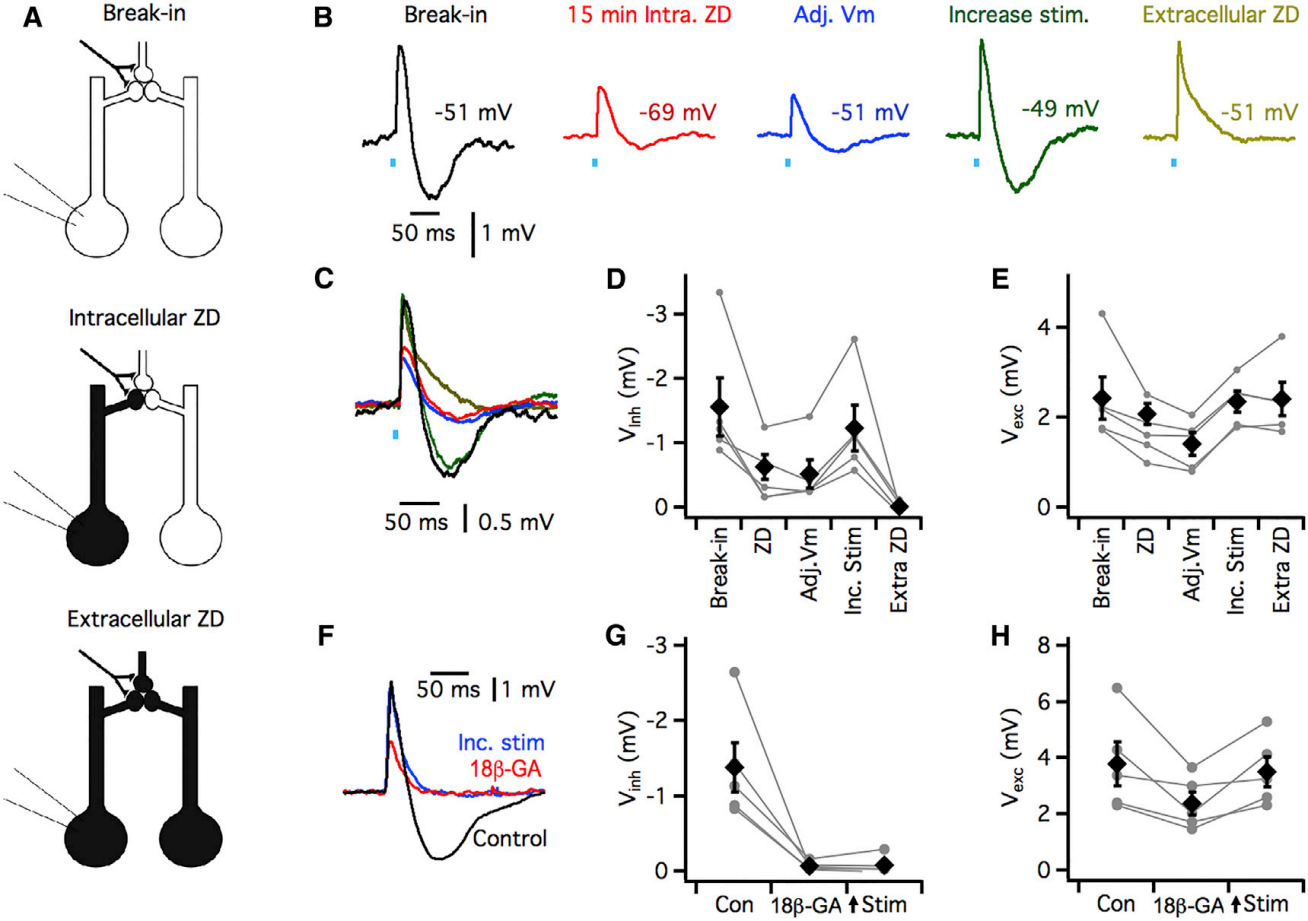
Gap-Junction-Mediated Long-Range Interactions Contribute to HCN1 Channel-Dependent Hyperpolarizing Components of Synaptic Responses (A) Schematic illustrating glomerular organization of synaptic inputs and strategy for blocking *I*_h_ only in the recorded cell (intracellular ZD) and in all cells (extracellular ZD). (B and C) Examples of synaptic responses recorded from a neuron in the IO of a Thy1-ChR2 mouse with 20 μM ZD7288 included in the intracellular solution. Response are shown in order (B), or overlaid (C), immediately following break-in (black), at 15 minutes following break-in (red), after adjusting the membrane potential to the value at break-in (blue), after increasing the stimulus intensity to restore the amplitude of the depolarizing component (green), and during bath application of 10 μM ZD7288 (yellow). (D and E) Summary plots of the amplitude of the inhibitory (D) and excitatory (E) components in each condition. Diamonds indicate mean ± SEM and circles indicate individual experiments. The amplitude of inhibitory and excitatory components depended on condition (V_inh_: F_4,16_ = 11.2 p = 1.59 × 10^−4^; V_exc_: F_4,16_ = 6.403 p = 0.028, one-way repeated-measures ANOVA, n = 5). Inhibitory, but not excitatory, components were reduced in amplitude 15 minutes after intracellular perfusion of ZD7288 (p = 0.033 and p = 0.45, respectively, versus break-in, Fisher’s LSD), and after adjustment of the membrane potential to its initial level (p = 0.019 and p = 0.039), both components were not significantly different from their break-in value after increasing the stimulus intensity (p = 0.43 and 0.86), and the inhibitory component was abolished in extracellular ZD (p = 0.001 and 0.95). (F) Examples of synaptic responses recorded from a neuron in the IO of a Thy1-ChR2 mouse in control conditions and then during bath application of the gap junction blocker 18β-glycyrrhetinic acid (18β-GA) (150 μM). (G and H) The amplitudes of inhibitory (p = 0.0008, paired t test, n = 4) (G) and excitatory (p = 0.0007) (H) components of the synaptic response were reduced in 18β-GA. Diamonds indicate mean ± SEM, and circles indicate individual experiments.

Excitatory synapses in the IO are organized in glomeruli, which may mediate interactions between adjacent postsynaptic neurons. Each glomerulus contains up to 8 dendritic spines that originate from different IO neurons and are connected to one another by gap junctions (de Zeeuw et al., 1990) (Figure 3A). A theoretical model of synaptic integration in the IO predicts that excitatory input will trigger local spikes that propagate between spines within a glomerulus via gap junctions and that will appear as bidirectional responses at the soma of each neuron (Kistler and De Zeeuw, 2005). Our observation of bidirectional glutamatergic synaptic responses that are relatively insensitive to somatic membrane potential is consistent with predictions of this model (Figure 2; Garden et al., 2017). We therefore reasoned that the actions of HCN1 channels on synaptic responses may in part originate from neurons electrically connected to the recorded cell. In this case, we expect that whereas block of *I*_h_ in all connected neurons will abolish the inhibitory component of synaptic responses (Figures 1 and 2), block of *I*_h_ in only the recorded cell will not (cf. Figure 3A). In addition, the inhibitory component might also be sensitive to block of gap junction connections between IO neurons. We therefore set out to test these predictions.

We used intracellular delivery of ZD7288 to block HCN1 channels in the recorded neuron without affecting HCN1 channels in other cells in the network (Figures 3A–3E). Over the first 10 minutes of recording with ZD7288 included in the intracellular solution, the membrane potential of IO neurons hyperpolarized and sag responses were abolished, indicating block of *I*_h_ (Figures S3A–S3E). No further change in membrane potential or sag was observed, indicating that block of *I*_h_ in the recorded neuron was complete. In parallel with the hyperpolarization of the membrane potential, the amplitude of inhibitory (p = 0.033 versus break-in, Fisher’s LSD, n = 5), but not excitatory (p = 0.45 versus break in, Fisher’s LSD), components of glutamatergic synaptic responses were reduced, but neither component was abolished (Figures 3B–3E). Strikingly, we found that increasing the intensity of synaptic stimulation rescued the inhibitory component of the synaptic response (p = 0.43 versus break-in, Fisher’s LSD) (Figures 3B–3E). Therefore, HCN1 channels in the recorded neuron are not necessary for the inhibitory component of synaptic responses. To check whether in this experiment the inhibitory component nevertheless requires HCN1 channels, we subsequently bath applied ZD7288. We again found that the inhibitory component was abolished (p = 0.001 versus break in, Fisher’s LSD), suggesting that it reflects a network-wide requirement for HCN1 channels (Figures 3B–3E).

If the inhibitory component of glutamatergic synaptic responses reflects an action of HCN1 distributed across networks of electrically connected IO neurons, then it should also be sensitive to block of gap junctions. To test this possibility, we examined the effects on synaptic responses of bath application of either 18β-glycyrrhetinic acid (18β-GA) (Figures 3F–3H) or carbenoxolone (Figure S3F–S3H), which have both previously been shown to specifically block gap-junction-mediated electrical communication between IO neurons (Leznik and Llinás, 2005, Placantonakis et al., 2006). Application of 18β-GA for 10 min resulted in a moderate increase in input resistance (control 31.8 ± 2.2 MΩ, 18β-GA 37.6 ± 0.7 MΩ, p = 0.15, n = 5, paired t test) without any change in membrane potential (control −53.7 ± 1.9 mV, 18β-GA −54.9 ± 1.5 mV, p = 0.57, n = 5, paired t test). We found that block of gap junctions with 18β-GA reduced the amplitude of the depolarizing component of the glutamatergic synaptic response (p = 0.044, n = 5, paired t test) and completely abolished the hyperpolarizing component (p = 0.017, n = 5, paired t test) (Figures 3F–3H). The hyperpolarizing component remained absent when we increased the stimulus intensity to restore the depolarizing component (Figures 3F–3H). We obtained similar results using carbenoxolone (Figures S3F–S3H). These data support the idea that the HCN1 channels in adjoining electrically connected neurons contribute to the inhibitory component of glutamatergic synaptic responses.

### HCN1 Channels Regulate Spiking Properties of IO Neurons by Controlling their Somatic Resting Membrane Potential

**Figure 4:**
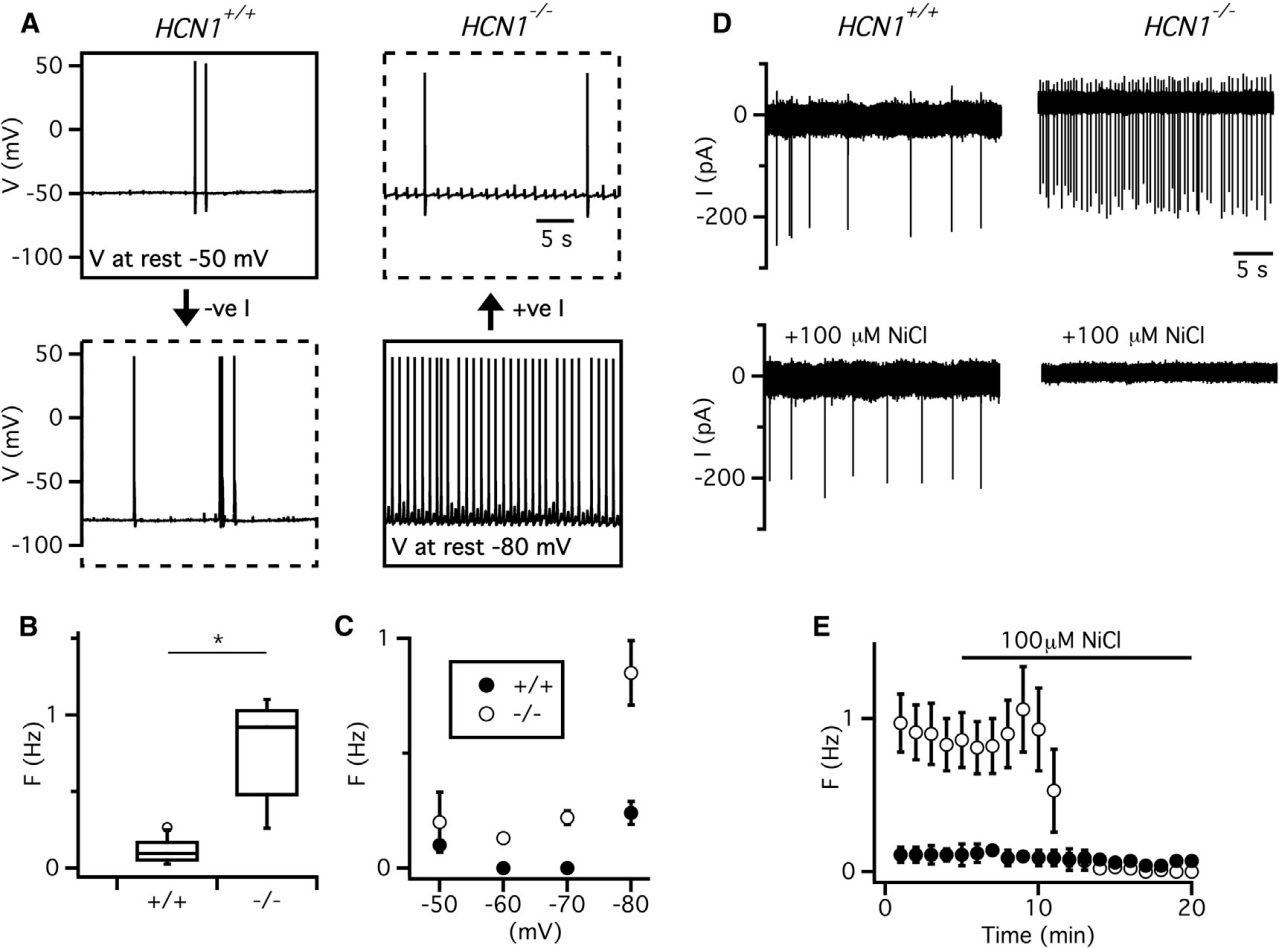
Deletion of HCN1 Increases Spontaneous Action Potential Firing by Recruiting T-Type Ca^2+^ Channels (A) Membrane potential recordings illustrating spontaneous activity of IO neurons recorded from *HCN1*^*+/+*^ mice (left) and *HCN1*^−/−^ mice (right). Examples show resting activity (solid line boxes) and activity when the membrane potential is adjusted by injection of current so that in *HCN*^*+/+*^ mice it is comparable to the resting value from *HCN*^−/−^ mice and vice versa (broken line boxes). (B) Boxplots of action potential frequency at the resting membrane potential (p = 7 × 10^−5^, t test, n = 9 in each group). (C) Plot of spike frequency as a function of membrane potential (F_1,56_ = 16.4 p = 0.0001 for effect of genotype; F_3,56_ = 8.7 p = 8.02 × 10^−5^ for interaction between genotype and membrane potential, ANOVA, n = 8 for both groups). (D) Examples of cell attached recordings from IO neurons from *HCN1*^*+/+*^ mice (left) and *HCN1*^−/−^ mice (right) in control conditions (upper) and during block of T-type Ca^2+^ channels with Ni^2+^ (lower). (E) Mean spike frequency versus time for effect of Ni^2+^. ANOVA indicated a significant effect on spike frequency of genotype (F_1,12_ = 9.5 p = 0.0096) and Ni^2+^ (F_1,12_ = 24.9 p = 0.0003) and a significant interaction between the two manipulations (F_1,12_ = 12.9 p = 0.0037). Post hoc tests indicate that Ni^2+^ has no significant effect on frequency in *HCN*^*+/+*^ neurons (p = 0.99, Tukey’s HSD, n = 5) but reduced frequency in *HCN1*^−/−^ neurons (p = 0.0003). Error bars in (C) and (E) indicate SEM.

We next asked whether HCN1 channels control action potential initiation, as suggested by our pharmacological experiments (Figures 1G and 1H), and whether mechanisms similar to those controlling the inhibitory component of synaptic potentials are involved. We first investigated spontaneous action potential firing. Strikingly, and distinct from suggested pacemaker roles of *I*_h_ (Bal and McCormick, 1997), the frequency of spontaneous action potentials fired by IO neurons was increased by deletion of HCN1 (p = 7 × 10^−5^, unpaired t test) (Figures 4A and 4B). Unlike the inhibitory glutamatergic synaptic responses, which are not restored by depolarization of the somatic membrane potential (Figure 2), when the somatic membrane potential of neurons from *HCN1*^−/−^ mice was adjusted to approximately −50 mV, the frequency of spontaneous action potentials became indistinguishable from *HCN1*^*+/+*^ mice (p = 0.82, unpaired t test) (Figure 4C). Application of the blocker ZD7288 to neurons from *HCN1*^*+/+*^ mice produced effects similar to deletion of HCN1 (Figure S4). Moreover, differences between *HCN1*^*+/+*^ and *HCN1*^−/−^ mice in their resting potential and spike frequency where abolished by ZD7288, indicating that these effects of HCN1 deletion also result directly from the absence of currents mediated by HCN1 channels and not from secondary adaptations (Figure S4). We also addressed possible effects of HCN1 on sinusoidal subthreshold oscillations. We observed sinusoidal subthreshold oscillations at resting potential in ∼33% of *HCN1*^*+/+*^ mice (n = 9/27), which is consistent with previous observations *in vivo* and *in vitro* (Khosrovani et al., 2007). We did not observe resting sinusoidal subthreshold oscillations from any *HCN1*^−/−^ mice (n = 0/27), while the membrane potential at potentials equivalent to the resting potential of *HCN1*^*+/+*^ mice was dominated by ongoing asymmetric spikelet activity (Figure 4A), which likely reflects spontaneous spiking by IO neurons electrically coupled to the recorded cell.

The increase in firing rate following deletion of HCN1 is at first paradoxical given the associated profound hyperpolarization of the membrane potential. However, hyperpolarization of IO neurons can promote action potential firing through recruitment of T-type Ca^2+^ channels (Llinás and Yarom, 1981b). Consistent with this mechanism, we found that bath application of Ni^+^, which blocks T-type channels (Lee et al., 1999), abolished spontaneous action potential firing in *HCN1*^−/−^ mice (p = 0.0003, Tukey’s HSD, n = 5), but not *HCN1*^*+/+*^ mice (p = 0.99, Tukey’s HSD) (Figures 4D and 4E). This differential effect of Ni^+^ does not result from upregulation of T-type channels following deletion of HCN1, as the amplitude and kinetics of T-type currents were similar in neurons from both groups of mice (p = 0.79, unpaired t test) (Figure S5). Thus, HCN1 channels in IO neurons suppress spontaneous firing by driving voltage-dependent inactivation of somatic T-type channels. This effect of HCN1 channels can explain why the threshold and probability of synaptically driven spike firing is not affected by block of *I*_h_, even though there is a profound membrane potential hyperpolarization (Figures 1E and 1F).

**Figure 5:**
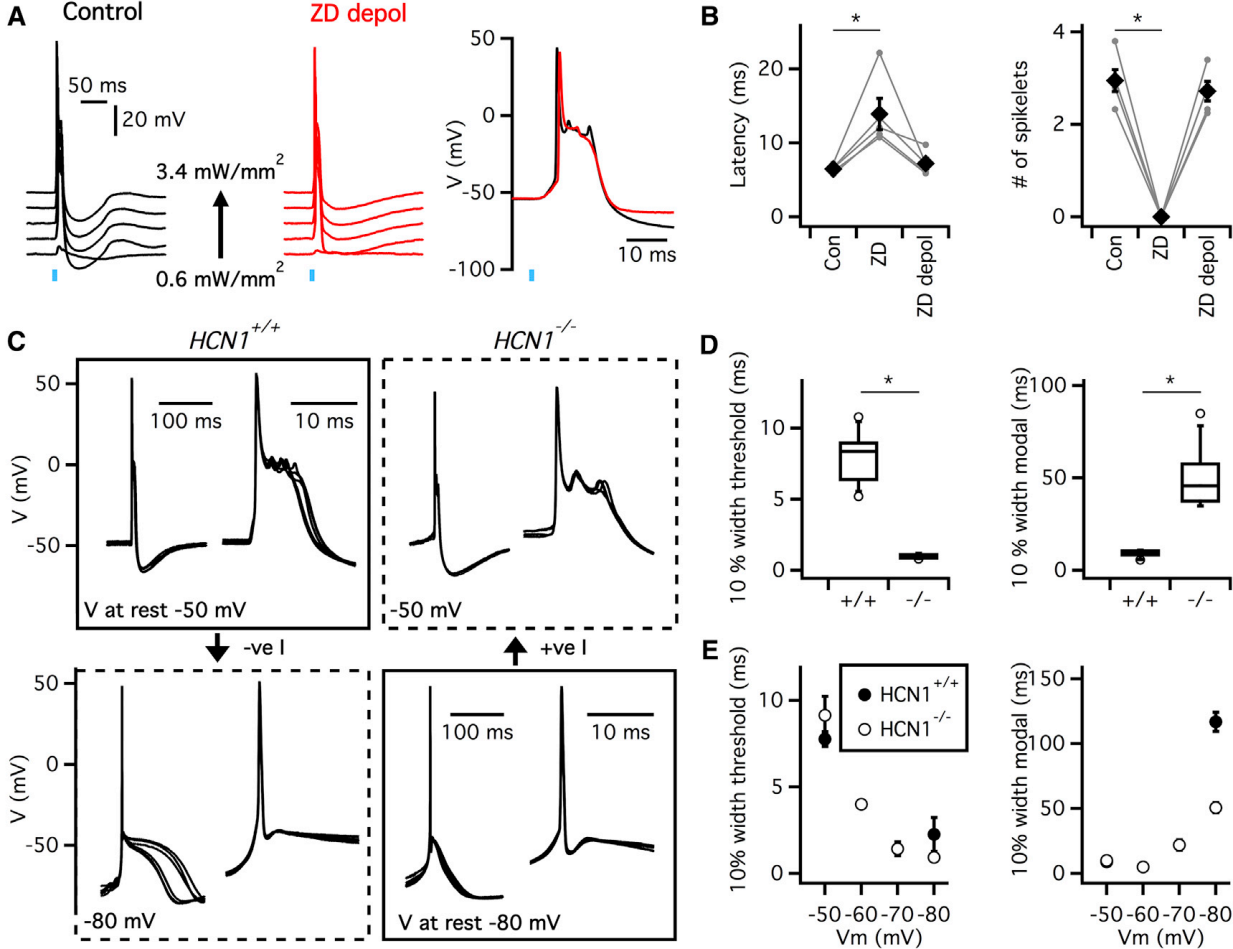
Waveforms of Spontaneous Action Potentials Are Modified by Deletion of HCN1 (A) Examples of synaptic responses of IO neurons from Thy1-Chr2 mice to varying intensity optical stimulation in control conditions (left) and during perfusion of ZD7288 while also injecting positive current to restore the membrane potential to its control value (center). Threshold spikes shown on an expanded timescale indicate that spike latency and number of spikelets are similar (right). (B) Latency (left) and number of spikelets (right) for synaptically driven action potentials activated in control conditions (Con), during perfusion of ZD7288 (ZD) and during perfusion of ZD7288 with the membrane potential restored to its control value (ZD depol). Perfusion of ZD7288 modifies the latency (F_2,8_ = 11.5 p = 0.004 for effect of condition, one-way repeated-measures ANOVA; control versus ZD7288, p = 0.015, Fisher’s LSD, n = 5) and number of spikelets (F_2,8_ = 149.5 p = 4.6 × 10^−7^ and p = 8.4 × 10^−8^, respectively), but after restoration of the somatic membrane potential, both are indistinguishable from their control values (control versus ZD depol, p = 0.68 and p = 0.39 respectively, Fisher’s LSD, n = 5). (C) Voltage waveforms of action potentials recorded from IO neurons in the absence of injected current (solid line boxes) and when the membrane potential is adjusted by injection of negative (*HCN1*^*+/+*^) or positive current (*HCN1*^−/−^) (broken line boxes). In each box waveforms to the right show the action potentials on an expanded timescale. (D) Boxplots of width of the action potential waveform measured relative to the estimated threshold for initiation of the action potential (p = 4.3 × 10^−10^, t test, n = 9) and width of the action potential complex measured relative to the modal membrane potential (p = 6.0 × 10^−7^). (E) Width of the spike (F_1,24_ = 37.9 p = 2.3 × 10^−6^ for interaction between genotype and membrane potential, ANOVA) (left) and the spike complex (F_1,24_ = 31.9 p = 8.2 × 10^−6^ effect of genotype, two-way ANOVA) (right), plotted as a function of membrane potential. Error bars in (B) and (E) indicate SEM.

Do the effects of HCN1 channels on the somatic resting membrane potential also explain changes in the latency and waveform of synaptically driven action potentials observed following pharmacological block of *I*_h_? If they do, then these changes should also be reversed by depolarization of the somatic membrane potential. Consistent with this prediction, we found that during pharmacological block of *I*_h_, the latency of synaptically driven action potentials was again increased (p = 0.018, Fisher’s LSD, n = 5) and spikelets were abolished (p = 2.54 × 10^−4^), but following depolarization of the somatic membrane potential the spike latency (p = 0.28) and number of spikelets (p = 0.19) were indistinguishable from their values prior to application of ZD7288 (Figures 5A and 5B). If the sensitivity of spike content to ZD7288 reflects specific block of HCN1 channels then similar changes in the number of spikelets associated with spontaneous action potentials should be found in HCN1^−/−^ mice. Indeed, there were 2.04 ± 0.38 spikelets during the ADP of spontaneous action potentials from HCN1^+/+^ mice, whereas spikelets were completely absent during the ADP of spontaneous action potentials in HCN1^−/−^ mice (0 ± 0 spikelets, n = 9) (Figure 5C). These and other measures of spike shape, which were also modified by deletion of HCN1 (Figures 5C and 5D) or pharmacological block of *I*_h_ (Figure S4), were rescued by restoration of the somatic membrane potential and were mimicked by somatic hyperpolarization of IO neurons from wild-type mice (Figures 5C, 5E, and S4D–S4F). Together, these data indicate that the influence of HCN1 channels on a neuron’s somatic membrane potential controls its spontaneous and synaptically driven action potentials.

### Deletion of HCN1 Increases Variability in Cerebellar Complex Spike Patterns Recorded during Quiet Wakefulness and Movement

**Figure 6:**
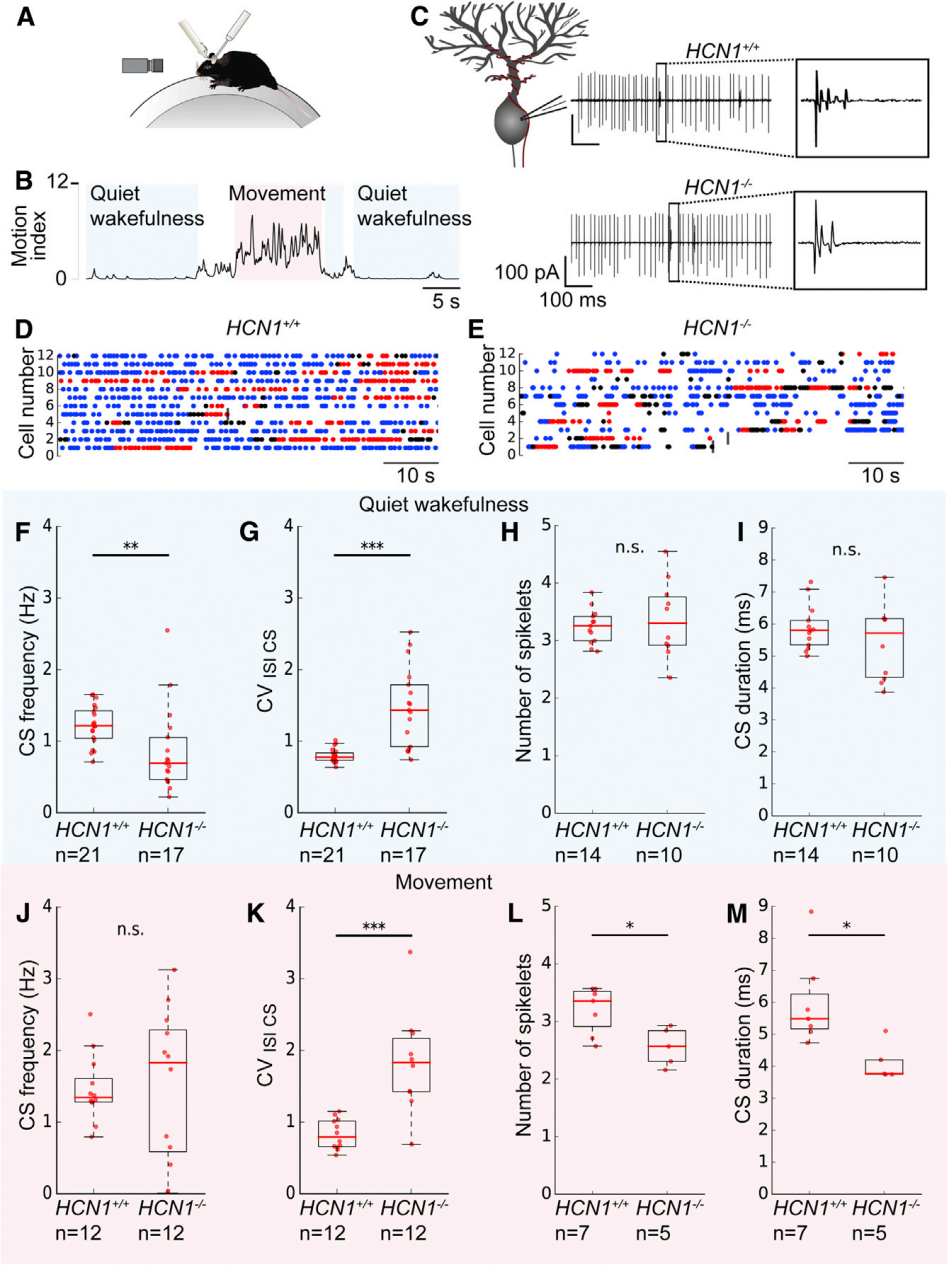
Deletion of HCN1 Increases Variability in the Timing of Complex Spikes Recorded from Cerebellar Purkinje Cells (A) Schematic of the *in vivo* recording setup. The head-fixed mouse is able to run on a cylindrical treadmill. A recording electrode is lowered through the cerebellar cortex to reach the Purkinje cell layer. (B) A motion index is calculated based on video analysis to determine clear periods of quiet wakefulness and movement. (C) Examples of cell-attached patch clamp recordings from *HCN1*^*+/+*^ and *HCN1*^−/−^ Purkinje cells showing simple spikes and complex spikes. The enlarged area shows complex spike waveforms. (D and E) Examples of the distribution of complex spikes from individual Purkinje cells over time in *HCN1*^*+/+*^ mice (D) and in *HCN1*^−/−^ mice (E). Each line represents data from one Purkinje cell. Blue dots indicate a complex spike during a period of quiet wakefulness, and red dots indicate a complex spike during a period of movement. Black dots indicate a complex spike in a period that cannot be clearly defined as either quite wakefulness or movement. A maximum of 70 s is shown, or less if the recording was of a shorter duration, in which case a vertical dark gray line indicates the end of the recording. (F–I) During quiet wakefulness, complex spikes in *HCN1*^−/−^ mice have reduced frequency (p = 0.002, Mann-Whitney U test) (F) and increased CV (p = 0.000) (G) compared to *HCN1*^*+/+*^ mice. The number of spikelets (p = 0.828) (H) and complex spike duration (p = 0.734) (I) do not differ significantly (Table S1). (J–M) During movement, *HCN1*^−/−^ mice show an increase in the CV of complex spike firing compared to *HCN1*^*+/+*^ mice (p = 0.001, Mann-Whitney *U* test) (K) but no change in complex spike frequency (p = 0.840) (J). The number of spikelets per complex spike (p = 0.034) (L) and complex spike duration (p = 0.014) (M) are both also reduced (Table S1).

Given the striking influence of HCN1 channels in the IO on synaptic integration and action potential initiation that we describe above, along with their previously described roles in pacemaking and resonance (Bal and McCormick, 1997, Matsumoto-Makidono et al., 2016), we asked whether HCN1 channels influence activity of IO neurons in behaving animals. As the IO is relatively difficult to access for direct electrophysiological recordings, we instead addressed this question using cell-attached recordings of complex spike activity from cerebellar Purkinje cells in awake, head-fixed mice (Figures 6A–6C). Because IO action potentials reliably trigger Purkinje cell complex spikes, and because complex spike duration is proportional to the number of spikelets in the IO spike (Mathy et al., 2009), these properties serve as a readout of activity in the IO (Eccles et al., 1964). We focused on Purkinje cells in the vermis of lobule V of the cerebellum, as this region integrates sensory input with motor commands and is involved in adaptive motor coordination (Apps and Hawkes, 2009).

We compared complex spike activity between *HCN1*^*+/+*^ and *HCN1*^−/−^ mice during quiet wakefulness and periods of movement (Figures 6D–6E). We find that deletion of HCN1 causes a striking change in the coefficient of variation (CV) of the interval between complex spikes in both behavioral states (Figures 6G, 6K, S6A, and S6B; Table S1). This was manifest as an increase in the mean CV across the population and in the variability of the CV between cells (Table S1). These changes in firing pattern were accompanied by a smaller reduction in complex spike frequency during quiet wakefulness, but not during movement (Figures 6F and 6J; Table S1). When we evaluated the number of spikelets and complex spike duration, we did not find significant differences during quiet wakefulness (Figures 6H and 6I), but both were reduced during movement (Figures 6L and 6M). We also investigated the effect of movement within cells. We did not find significant differences in any of the measured complex spike properties between quiet wakefulness and movement in either *HCN1*^*+/+*^ or *HCN1*^−/−^ mice (Figures S6C–S6N; Table S2; see also Jelitai et al., 2016). The frequency of simple spikes was not affected by a global deletion of HCN1 (Table S1). Thus, HCN1 channels do not appear to impact the frequency of simple spikes fired by cerebellar Purkinje cells and have relatively little effect on the frequency of complex spikes originating from the IO. Instead, these data are consistent with HCN1 channels influencing the timing of action potential firing in the IO and the number of spikelets following an action potential during movement.

## Discussion

Our results establish local and network-wide roles for HCN1 channels in control of synaptic integration in the IO and provide evidence that in behaving animals HCN1 channels in the IO influence spike timing. We find that HCN1 channels are required for *I*_h_ in IO neurons and contribute substantially to their resting and active membrane properties. HCN1 channels acting in part via gap junctions from adjoining IO neurons enable the inhibitory component of the PSP, whereas HCN1 channels acting at the soma control the timing and content of action potentials fired by IO neurons. The combination of local and long-distance actions of HCN1 channels, which contrast with their roles in other neuron types, supports the idea that the IO carries out distinct network-level computations.

### Distal HCN1 Channel Signaling Enables Inhibitory Components of Glutamatergic Synaptic Responses

How do HCN1 channels enable the hyperpolarizing component of the response to glutamatergic synaptic input? How can this requirement for HCN1 channels be reconciled with previous findings that the hyperpolarizing component is mediated by calcium-activated potassium channels (Garden et al., 2017)? By demonstrating that inhibitory components of bidirectional GluA-mediated synaptic responses are abolished by pharmacological and genetic manipulations of HCN1 channels, we provide direct evidence for a role of HCN1 while also ruling out off-target effects of *I*_h_ blockers (Felix et al., 2003) or adaptation following genetic manipulations (Chen et al., 2010). The direct contribution of *I*_h_ to the resting membrane conductance is unlikely to explain the requirement of *I*_h_ for inhibitory responses, as in this case, blocking *I*_h_ would substantially increase the amplitude of the depolarizing response (cf. Stuart and Spruston, 1998), whereas the maximal amplitude of this component is either maintained or slightly reduced after deletion of HCN1 or block of *I*_h_ (Figures 2 and 3). Instead, our observations can be explained in the framework of a two-stage model of synaptic integration by IO neurons (Kistler and De Zeeuw, 2005). According to this model, the hyperpolarizing component of the glutamatergic PSP is mediated by Ca^2+^-activated potassium channels, whose opening is driven by local Ca^2+^ spikes triggered by the depolarizing component of the synaptic response (Kistler and De Zeeuw, 2005). This model is supported by pharmacological and electrophysiological analysis of glutamatergic inputs to neurons in the IO (Garden et al., 2017). In this scenario, resting activation of HCN1 channels located on dendrites (Matsumoto-Makidono et al., 2016) will maintain depolarization of spines within synaptic glomeruli, which in turn enables synaptic input to trigger dendritic Ca^2+^ spikes. Thus, in the absence of HCN1, the dendrite is hyperpolarized and glutamatergic input can no longer trigger the Ca^2+^ spikes. This may explain the reduction in the peak of the depolarizing components of the glutamatergic PSP following deletion of HCN1 (Figure 2) and pharmacological block of *I*_h_ (Figure 3), while the absence of a resulting Ca^2+^ influx and activation of Ca^2+^-activated potassium channels can account for the abolished hyperpolarizing response to glutamatergic inputs.

Our results suggest that the control of hyperpolarizing components of the PSP by HCN1 channels involves network-wide actions mediated by gap junctions. Whereas functions of HCN1 channels are usually restricted to the neuron in which they are expressed (Magee, 2000), differential actions of intracellular and extracellular block of *I*_h_, along with effects of gap junction block, indicate that the inhibitory components of PSPs involve actions of HCN1 channels in adjoining electrically connected neurons (Figure 3). The relative insensitivity of synaptic responses to changes in the somatic membrane potential of the recorded cell (Figure 4) is also consistent with synaptic potentials originating at locations that are electrically distant from the soma. Given that glomeruli at which IO neurons receive excitatory inputs are connected by gap junctions (Kistler and De Zeeuw, 2005), HCN1 channels in all cells contributing to a glomerulus may permit bidirectional responses by maintaining the membrane potential of spines in a depolarized state so that subsequent excitatory input can trigger local spikes. Therefore, inhibitory components of the synaptic response should still be present when a HCN1^+/+^ neuron is hyperpolarized or when HCN channels are blocked by intracellular ZD7288. This is consistent with our experimental observations (Figure 3). In contrast, if depolarization from HCN1 channels is absent in all neurons and all of the spines within the glomeruli are hyperpolarized, then synaptic activation of dendritic Ca^2+^ spikes and subsequent activation of small conductance (SK) or large conductance (BK) calcium-activated potassium channels becomes unlikely, and no inhibitory component will be recorded at the soma. This is consistent with our recordings both from HCN1^−/−^ mice and from HCN1^+/+^ mice during extracellular application of ZD7288 (Figure 3). These interactions between gap junctions, intrinsic properties, and synaptic input appear distinct from synaptic integration in largely passive dendrites of cerebellar Golgi cells, which are also connected by gap junctions (Vervaeke et al., 2012).

### Local Actions of HCN1 Channels Controls Action Potential Initiation and Content

In addition to controlling subthreshold integration, we found that HCN1 channels reduce the latency for synaptically driven action potential firing and support generation of spikelets during the action potential afterdepolarization (Figures 1 and 5). In several neuronal cell types that generate action potentials spontaneously, *I*_h_ is thought to act as an excitatory pacemaker current (Robinson and Siegelbaum, 2003). In contrast, in IO neurons, HCN1 channels reduce the frequency of spontaneous action potential firing but are required for the prolonged action potential ADP and its superimposed spikelets (Figures 4 and 5). These observations are initially paradoxical, as inward current flowing through HCN1 channels should drive pacemaking at resting potentials (Bal and McCormick, 1997), while HCN1 channels close at positive membrane potentials reached during the action potential ADP (Figure 1C). However, they are consistent with previous reports that T-type calcium channels drive spontaneous activity of hyperpolarized IO neurons (Llinás and Yarom, 1986) and observations of the voltage-dependence of the ADP (Llinás and Yarom, 1981a). Thus, the depolarizing influence of HCN1 channels causes inactivation of T-type Ca^2+^ channels, which prevents T-type Ca^2+^ channels from driving spontaneous firing, and enables the spike ADP. This interpretation is consistent with the reversibility of these phenotypes by somatic depolarization (Figures 4 and 5) and a lack of evidence for adaptation by other membrane conductances following deletion of HCN1 (Figures S5). In other neuron types, *I*_h_ also controls excitability via its actions on membrane potential, causing modified gating of voltage-gated ion channels (George et al., 2009). Just as for the influence of HCN1 channels on the excitability of IO neurons, these functions reflect influence of *I*_h_ on signaling within the recorded cell.

### Implications for Computation by IO Networks during Motor Behavior

How do HCN1 channels influence the firing of IO neurons during behavior? Using complex spike firing by Purkinje cells as a readout of firing by neurons in the IO, we find that *in vivo* HCN1 channels primarily affect the pattern of climbing fiber activity. This is apparent as a striking increase in the variability of the interval between complex spikes. This increase in complex spike CV occurs both during quiet wakefulness and during movement (Figures 6D, 6E, 6G, and 6K). A dominant role for HCN1 in controlling synaptic integration within the IO may be consistent with these observations. Thus, HCN1 determines the timing of action potentials triggered by the depolarizing component of glutamatergic PSPs (Figure 1), and because it is required for the hyperpolarizing component of the PSP (Figures 1 and 2), it should also determine the spatial and temporal integration of bidirectional glutamatergic responses (cf. Garden et al., 2017). The changes to spike timing *in vivo* appear unlikely to be accounted for by a direct influence of HCN1 channels on spontaneous spiking of IO neurons, as we find that *in vitro*, when background synaptic activity is absent, HCN1 channels suppress spontaneous firing and increase the variability of spike intervals (Figure 4). This is the opposite of our finding in behaving animals. This difference may be because *in vivo* glutamatergic synaptic input drives spontaneous activity of IO neurons (Lang, 2001). Resonant or oscillatory roles of HCN1 channels also appear unlikely to explain the increase in variability of complex spike timing, as both would act on timescales in the 5- to 10-Hz range (interspike intervals of 100–200 ms), whereas the interspike intervals that contribute to increased variability are much longer (Figure S6). The changes in firing pattern are also unlikely to result from an absence of HCN1 from cerebellar Purkinje cells, as deletion of HCN1 channels does not affect the initiation or properties of Purkinje cell complex spikes (Rinaldi et al., 2013). Together, these observations indicate that models for computation by the IO must account for multiple complementary roles of HCN1 channels and point toward the importance of the influence of excitability on synaptic integration within the IO. These changes would be expected to influence motor coordination through altered signaling and plasticity in the cerebellar cortex; for example, through climbing-fiber-driven modification of parallel fiber input to Purkinje cells (Mathy et al., 2009), as well as modifications to climbing fiber synapses and intrinsic plasticity in Purkinje cells (Grasselli et al., 2016, Hansel and Linden, 2000, Ohtsuki et al., 2012).

In conclusion, our results indicate that integrative mechanisms in the IO are engaged during movement. HCN1 channels have both local and long-range actions in the IO, while their disruption modifies patterns of IO firing during behavior. This diversity of cellular functions for a single ion channel is consistent with the evolution of combinatorial patterns of ion channel expression that enable particular neuron types to perform specific computations (Marder and Goaillard, 2006).

## Experimental Procedures

Further details and an outline of methods and resources used in this work can be found in Supplemental Experimental Procedures.

### Animals

Experimental studies conformed to the policies of the UK Animals (Scientific Procedures) Act 1986 and European Directive 2010/62/EU on the protection of animals used for experimental purposes. Experiments were carried out under a project license granted by the UK Home Office and according to the guidelines laid down by the University of Edinburgh’s Animal Welfare Committee.

C57BL/6 mice (all males), mice expressing ChR2 under the control of the Thy1 promoter (Thy1-ChR2-YFP line 18, stock number 007612, The Jackson Laboratory, Bar Harbor, ME) (Arenkiel et al., 2007), and mice with a global deletion of HCN1 (*HCN1*^−/−^; Nolan et al., 2003) and their wild-type littermates (*HCN*^*+/+*^) (both males and females) were housed on a 12-hr light/dark cycle (light on 7:00–19.00 hr) in standard breeding cages. Food and water were available *ad libitum*. For brain slice experiments, the median age of mice used was 46 days (range 28–116 days). For *in vivo* experiments, the median age of mice used was 56.5 days (range, 48–76 days). During all experiments, the experimenter was blind to the group the mice were in.

### Data Analysis and Statistical Methods

*In vitro* electrophysiological data were analyzed in IGOR pro (Wavemetrics) using Neuromatic (http://www.neuromatic.thinkrandom.com/) and custom-written routines or Axograph. *In vivo* electrophysiological data were analyzed using custom-written programs in Python (https://www.python.org). Simple spikes, complex spikes, and their associated spikelets were automatically detected and then visually verified. The reported number of spikelets per complex spike excludes the initial sodium spike component. Complex spike duration was defined as the time between the peak of the first sodium spike of the complex spike to the peak of the last spikelet of the same complex spike. Further statistical analysis was carried out using Python, IGOR pro, Excel (Microsoft), IBM SPSS Statistics version 17.0 (NY, USA), or R (www.R-project.org). Mean values are reported as ± SEM. Statistical significance was tested with linear regression, Student’s t test, one-way ANOVA, and post hoc Fisher’s LSD or Tukey’s HSD where appropriate, two-way repeated-measures ANOVA, the Kolmogorov-Smirnov test, or the Mann-Whitney *U* test.

## Supplemental Information

Document S1. Supplemental Experimental Procedures, Figures S1–S6, and Tables S1 and S2

Document S2. Article plus Supplemental Information
