# Supplementary material for: Inferior Olive HCN1 Channels Coordinate Synaptic Integration and Complex Spike Timing
Source: Cell Rep. 2018 Feb 13;22(7):1722–33. doi: 10.1016/j.celrep.2018.01.069 (PMC5847187; doi:10.1016/j.celrep.2018.01.069)
Supplement: Document S1. Supplemental Experimental Procedures, Figures S1–S6, and Tables S1 and S2 [file mmc1.pdf]

Cell Reports, Volume *22*

Supplemental Information

Inferior Olive HCN1 Channels Coordinate Synaptic Integration and Complex Spike Timing

Derek L.F. Garden, Marlies Oostland, Marta Jelitai, Arianna Rinaldi, Ian Duguid, and Matthew F. Nolan

SUPPLEMENTAL EXPERIMENTAL PROCEDURES

CONTACT FOR REAGENT AND RESOURCE SHARING

Matthew Nolan (mattnolan@ed.ac.uk) is the Lead Contact for reagent and resource sharing. All published reagents will be shared on an unrestricted basis; reagent requests should be directed to the lead author.

EXPERIMENTAL MODEL AND SUBJECT DETAILS

Experimental studies conformed to the policies of the UK Animals (Scientific Procedures) Act 1986 and European Directive 2010/62/EU on the protection of animals used for experimental purposes. Experiments were carried out under a project licence granted by the UK Home Office and according to the guidelines laid down by the University of Edinburgh's Animal Welfare Committee.

C57BL/6 mice, all males, mice expressing ChR2 under the control of the Thy1 promoter (Thy1-ChR2-YFP line 18, stock number 007612 from The Jackson Laboratory, Barr Harbor, ME)(Arenkiel et al., 2007), and mice with a global deletion of HCN1 ($HCN1^{-/-}$, Nolan et al. 2003) and their wildtype littermates ($HCN^{+/+}$), both males and females, were housed on a 12h light/dark cycle (light on 7:00 – 19.00h) in standard breeding cages. Food and water were available ad libitum. During all experiments the experimenter was blind to the group the mice were in.

METHOD DETAILS

Virus injections. C57BL/6 mice, all males, aged 5–6 weeks, were anesthetized with isoflurane and kept on a feedback-controlled heating pad (Homeothermic Blanket System 50300, Stoelting). Post-operative pain was prevented by administering 0.05 mg/kg buprenorphine hydrochloride (Vetergesic) during the procedure, and by giving the animals access to Vetergesic in jelly form during recovery. Injections targeting the motor cortex were carried out as described previously (Garden et al., 2017). The mice were mounted into a stereotaxic frame and a cut was made to expose the skull. For

injections into the neocortex, holes were drilled in the skull bilaterally above areas containing primarily M1 and M2 (1.2-1.4 ML, 1.0-1.5 AP from bregma) and the underlying dura was carefully removed. A pipette was then inserted at a depth of 1 mm from the pial surface and 500 nl of adeno-associated virus (AAV) (pACAGW-ChR2-Venus, Vector biolabs) was injected over 5 minutes. Pipettes were left for 5 minutes post-injection before removal.

In vitro electrophysiology. Slice preparation and patch-clamp recording from neurons in the IO was as described previously (Garden et al., 2017). The median age of mice used was 46 days (range 28 - 116 days). We did not find any change in measured parameters with age of the mice. Mice were killed by decapitation following isoflurane anesthesia and their brains rapidly removed, and placed in cooled (4-6 °C) oxygenated modified artificial cerebrospinal fluid (ACSF) composed of the following (in mM): NaCl (86), KCl (2.5), $CaCl_2$ (0.5), $MgCl_2$ (7), NaH_2PO_4 (1.2), $NaHCO_3$ (25), glucose (25), and sucrose (75), continuously bubbled with 95 % O_2 and 5 % CO_2 (pH = 7.4). The brain was placed ventral-side up and a coronal cut made through the widest part of the brainstem and cerebellum. The cut surface was glued to the stage of a sectioning system (Leica VT1200), with the caudal part of the brain facing upwards. Coronal sections of thickness 200 µm were cut submerged under cold modified ACSF. After slicing, brain slices were immediately immersed in regular ACSF, consisting of the following (in mM): NaCl (124), KCl (2.5), $CaCl_2$ (2), $MgCl_2$(1), NaH_2PO_4 (1.2), $NaHCO_3$ (25), and glucose (20), continuously bubbled with 95 % O2 and 5 % CO2 (pH=7.4). Slices were kept at a temperature of 33 – 35°C for 10 – 20 mins and then passively cooled to room temperature (20 – 24°C). Neurons in the IO were visually identified under infrared illumination with DIC optics. Whole-cell recordings were obtained at 35°C–37°C from the soma of IO neurons using electrodes with resistance 2–5 MΩ when filled with intracellular solution containing the following (in mM): K gluconate (130), KCl (10), EGTA (0.5), HEPES (10), MgCl (2), EGTA (0.1), NA_2ATP (2), NA_2GTP (0.3), and phosphocreatine (10)(pH adjusted to 7.3 with KOH). Recordings were made using a Multiclamp 700B amplifier (Molecular Devices, Sunnydale) and Axograph X software (Axograph Scientific, Sydney). Series resistances were < 15 MΩ for voltage-clamp experiments and < 40 MΩ for current-clamp experiments. Series resistance in voltage-clamp recordings was compensated by 70%–80%. For current-clamp recordings

appropriate bridge and electrode capacitance compensations were applied. Membrane current and voltage were filtered at 1–2 KHz and 4–20 KHz and sampled at 5–10 KHz and 10–50 KHz for voltage- and current-clamp experiments, respectively. Input resistance was calculated from the steady-state voltage response to injected 80 pA current steps. The sag was calculated as the steady-state voltage response to an injected negative current of 160 pA divided by the peak of this response. A lower value represents a larger membrane potential sag.

For optogenetic activation of ChR2 expressing axons an LED was attached to the epifluorescence port of the microscope used for identification of recorded neurons (see (Garden et al., 2017)). Activation of the LED was controlled by an analogue voltage output from a data acquisition board.

Measurement of I_h was carried out in ACSF of the following composition (in mM): NaCl (115), NaH_2PO_4 (1.2), KCl (5), $NaHCO_3$ (25), glucose (20), $CaCl_2$ (2), $MgCl_2$ (1), $BaCl_2$ (1), $CdCl_2$ (0.1), 4-AP (1), TEA (5), NBQX (0.005), picrotoxin (0.05), and TTX (0.0005). Experiments to examine the effects of pharmacological block of ion channels on the light-evoked responses were performed with blockers of ionotropic glutamate and GABA receptors added to the ACSF. All chemicals were purchased from Sigma (St. Louis, MO) with the exception of NBQX, D-AP5, picrotoxin and ZD7288 from Abcam biochemicals (Cambridge, UK). Drugs were made fresh daily from frozen stocks concentrated 1000-fold.

In vivo electrophysiology in awake mice. Mice with global deletion of HCN1 (*HCN1*$^{-/-}$) and their wild-type littermates (*HCN1*$^{+/+}$), both males and females, were obtained as previously described (Nolan et al. 2003). For all experiments, the mice were on a mixed average 50:50% 129SVEV:C57BL/6 background. Genotype was determined from ear notch biopsies by real-time PCR (Transnetyx, Cordova, TN, USA) and confirmed after each experiment using DNA from tail biopsies. Mice were housed in standard breeding cages with access to a running wheel for at least a week before the start of the experiment. Mice were kept on a reversed 12h light/dark cycle (light on 19:00 – 7:00h) for 2-4 weeks before the start of the experiment.

Surgical procedures and *in vivo* awake recordings were performed as previously described (Jelitai et al., 2016). All surgical procedures were performed under 1.5% isoflurane anaesthesia and with mice kept on a feedback-controlled heating pad (Homeothermic Blanket System 50300, Stoelting). A small lightweight headplate (0.75 g) was implanted using cyanoacrylate adhesive and dental acrylic (Jet Denture Repair, Lang Dental Manufacturing Co.) and sealed with a Kwik-Cast sealant (World Precision Instruments). Post-operative pain was prevented by administering 0.05 mg/kg buprenorphine hydrochloride (Vetergesic) during the procedure. After at least 24 hours of recovery, the Kwik-Cast sealant was removed and a craniectomy (~300 x 300 μm, using a Volvere Max GX35 hand drill, NSK Dental) was performed above lobule V of the cerebellum (2.5 mm posterior to lambda and 0.75 mm lateral to midline) and the dura removed. The craniectomy was sealed with agar (1.5%) and Kwik-Cast sealant and mice were returned to the home cage for ~1 hr to recover from anaesthesia before recording commenced. Post-operative pain was prevented by administering a non-steroidal, anti-inflammatory agent carprofen (4 mg/kg, Rimadyl) during the procedure.

In vivo cell-attached recordings were made from Purkinje cells in lobule V of the cerebellar vermis of awake mice head-fixed on a spherical treadmill. Mice were habituated to the head-restraint and experimental setup for 30-60 minutes before each recording session. Head-restrained mice were free to run, walk or sit on the cylindrical treadmill. The Kwik-Cast sealant was removed at the start of the recording. *In vivo* external solution consisted of the following (in mM): NaCl (150), KCl (2.5), HEPES (10), CaCl2 (1.5), MgCl2 (1), with pH 7.3. Glass pipettes (resistance 5-8 MΩ) were filled with internal solution consisting of (in mM): K-gluconate (135), KCl (7), HEPES (10), sodium phosphocreatine (10), MgATP (2), Na_2ATP (2), Na_2GTP (0.5), with pH 7.2 and 285-295 mOsm and lowered to the right depth at an angle of 60° using a micromanipulator (Scientifica). Biocytin (1-2 mg/ml) was added before recording. Cell-attached recordings were performed at 250-400 μm from the pial surface using a Multiclamp 700B amplifier (Molecular Devices, USA). The signal was filtered at 10 kHz and acquired at 20 kHz using PClamp 10 software using a DigiData 1440A DAC interface (Molecular Devices, USA). Purkinje cells were identified based on the occurrence of both simple spikes and complex spikes.

Recordings used for analysis lasted from 18 s to 372 s during quiet wakefulness (*HCN1*$^{+/+}$: mean average 166.9 ± 19.5 s, n = 21, in 7 mice; *HCN1*$^{-/-}$: mean average 185.3 ± 22.4 s, n = 17, in 9 mice) and from 5 s to 71 s during movement (*HCN1*$^{+/+}$: mean average 26.6 ± 7.2 s, n = 12, in 6 mice; *HCN1*$^{-/-}$: mean average 20.9 ± 3.5 s, n = 12 , in 6 mice). Each recording contained 10 – 539 complex spikes during quiet wakefulness (*HCN1*$^{+/+}$: mean average 194 ± 26 complex spikes; *HCN1*$^{-/-}$: mean average 135 ± 31 complex spikes) and 7 –95 complex spikes during movement (*HCN1*$^{+/+}$: mean average 38 ± 8 complex spikes; *HCN1*$^{-/-}$: mean average 33 ± 9 complex spikes).

Motion index. We used a motion index to separate periods of quiet wakefulness, in which the animal is not moving but seemingly still alert (not asleep), from periods were the animal was clearly moving. The motion index was calculated as described previously (Jelitai et al., 2016). All movements (positioning, grooming and locomotion) were captured using a digital camera (60 fps) and synchronized with each electrophysiological recording. We calculated the motion index for each successive frame: $MI_f=S^N{}_{i=1}(c_{f+1,i}-c_{f,i})2$, where $c_{f,i}$ is the grayscale level of the pixel i in frame f. Movement was defined as periods where the motion index was above 1 a.u. for at least 2 s. Periods of quiet wakefulness were defined as periods with a motion index below 1 a.u. for at least 2 s. Any periods which were not clearly movement or clearly quiet wakefulness were not analyzed. Visual inspection of each video confirmed the periods of movement (where the mice were either walking or running, but not grooming) and quiet wakefulness.

Immunohistochemistry. Procedures for immunohistochemical labeling of HCN1 channels are as described previously (Rinaldi et al., 2013). Mice were transcardially perfused and brains were left overnight in 4% PFA at 4°C, before being put in 30% sucrose for at least 36 hours at 4 ^{o}C. The caudal parts of the brains containing both cerebellum and brainstem were sliced coronally at a thickness of 40 µm at a freezing microtome (HM 450, Thermo Scientific) and embedded in O.C.T. compound (VWR). Slices were washed for 3×10 min in phosphate-buffered saline (PBS), and blocking was performed in 10 % normal goat serum (NGS) in PBS-0.5 % Triton (PBS-T) for 2 hours at room temperature. Primary polyclonal rabbit antibody against HCN1 (Neuromab) was incubated at a

dilution of 1:1000 in PBS-T with 1% NGS overnight at room temperature. Excess antibody was removed by washing 4×10 min in PBS, and secondary antibody (goat anti-rabbit Alexa 546, A11010, Molecular Probes) was incubated at a dilution of 1:1,000 in PBS-T with 1% NGS for 2 h. Slices were washed for 3×10 min in PBS and a 4,6-diamidino-2-phenylindole (DAPI) staining was performed for 5 min (1:6,000 dilution of staining solution in PBS, original staining solution stock 2 mg/ml, 32670 Sigma, in H_2O). Slices were washed 3×10 min in PBS and embedded with mowiol. Imaging of sections was carried out using a Nikon A1R confocal microscope.

Data analysis and statistical methods. *In vitro* electrophysiological data was analyzed in IGOR pro (Wavemetrics) using Neuromatic (http://www.neuromatic.thinkrandom.com/) and custom-written routines, or using Axograph. *In vivo* electrophysiological data was analyzed using custom-written programs in Python (www.python.org). Simple spikes, complex spikes and their associated spikelets were automatically detected and then visually verified. The reported number of spikelets per complex spike excludes the initial sodium spike component. Complex spike duration was defined as the time between the peak of the first sodium spike of the complex spike to the peak of the last spikelet of the same complex spike. Further statistical analysis was carried out using Python (www.python.org), IGOR pro, Excel (Microsoft), IBM SPSS Statistics version 17.0 (New York, USA), or R (www.R-project.org). Mean values are reported as ± standard error of the mean (SEM). Statistical significance was tested with with linear regression, Student's t test, one-way ANOVA and post hoc Fisher's LSD or Tukey's HSD where appropriate, two-way repeated measures ANOVA, Kolmogorov-Smirnov test, or the Mann-Whitney *U* test.

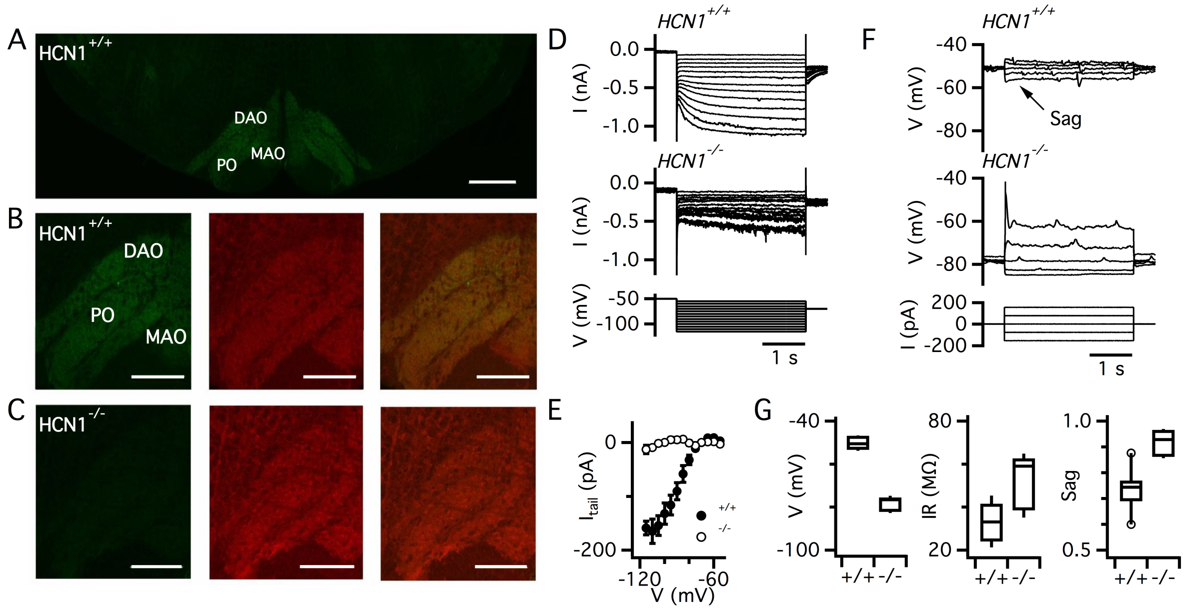

Figure S1. HCN1 channels mediate I_h in IO neurons (relates to Figure 2)

(A) Low magnification image of coronal brain stem section from a *HCN1*$^{+/+}$ mouse labelled with an antibody against HCN1 (green). DAO is dorsal accessory olive, PO is principal olive and MAO is medial accessory olive (scale bar 500 µm).

(B) Higher magnification view of the section in (A) illustrating double labeling with antibodies against HCN1 (left), MAP2 (centre) and merged (right)(scale bar 250 µm).

(C) Images of the IO from *HCN1*$^{-/-}$ mice labelled with antibodies against HCN1 (left), MAP2 (centre) and merged (right) (scale bar 250 µm).

(D) Voltage-clamp recordings of membrane current responses to voltage steps (lower) obtained from IO neurons in slices from *HCN1*$^{+/+}$ mice (upper) and *HCN1*$^{-/-}$ mice (middle).

(E) Plot of mean tail currents measured at -70 mV as a function of test potential. The maximum tail current amplitude for neurons from *HCN1*$^{+/+}$ mice was -172 ± 21 pA (n=9) compared with -12 ± 8 pA (n=9) for neurons from *HCN1*$^{-/-}$ mice (p=6.3 x10^{-4}, t-test).

(F) Examples of membrane potential responses to current steps (lower) recorded from IO neurons from *HCN1*$^{+/+}$ mice (upper) and *HCN1*$^{-/-}$ mice (middle).

(G) Box plots of modal resting membrane potential (p = 4.1 x 10^{-14}, t-test, n = 9)(left), input resistance (p = 7.0 x 10^{-4}) and sag (p = 8.8 x 10^{-6}).

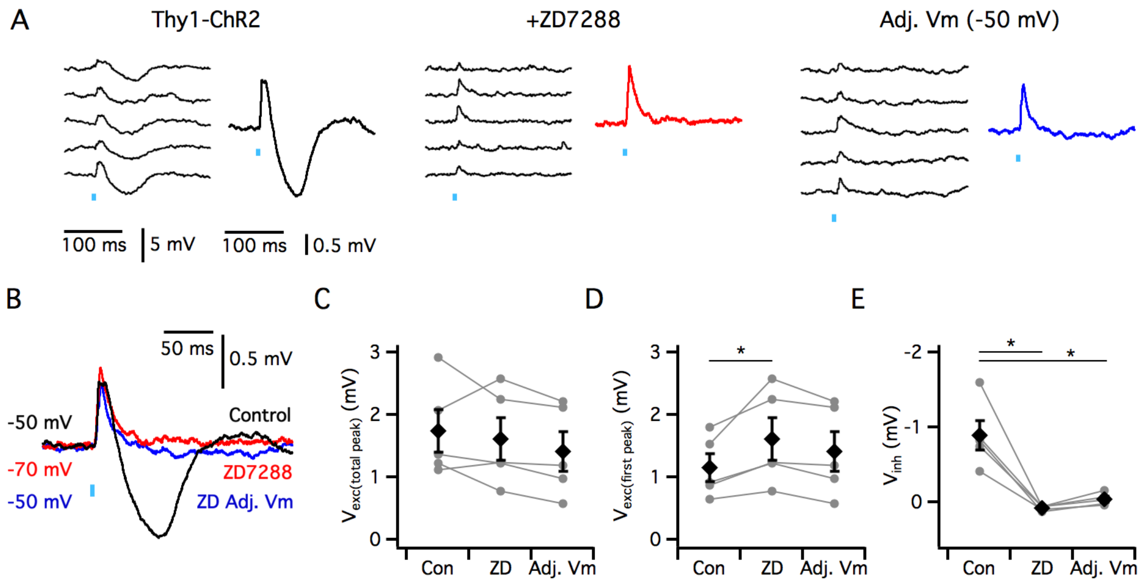

Figure S2. ZD7288-sensitive inhibitory component of PSPs is not rescued by depolarization (relates to Figure 2)

(A) Example responses of Thy1-ChR2 mice to optical activation in control conditions (left), and during application of ZD7288 in the absence of applied current (centre) and during injection of positive current to restore the membrane potential to its control value (right).

(B) Average responses superimposed for each condition in (A).

(C-E) Plots of the amplitude of the depolarizing (C, D) and hyperpolarizing (E) components for each condition. The maximum amplitude of the excitatory component (C) does not depend upon the condition ($F_{2,8}$ = 1.1 p = 0.37 one-way repeated measure ANOVA, n =5). The amplitude of the first peak (D) of the excitatory component depends on condition ($F_{2,8}$ = 8.1 p = 0.01, one-way repeated measure ANOVA), is increased by ZD7288 (Con v ZD p = 0.01, Fisher's LSD) and is restored by depolarization (Con v ZD AdjVm p = 0.22, Fisher's LSD). The amplitude of the inhibitory component (E) depends on condition ($F_{2,8}$ = 26.7 p = 0.007, one-way repeated measure ANOVA) is reduced by ZD7288 (Con vs ZD p = 6.4×10^{-5}, Fisher's LSD) and is not restored by subsequent depolarization (Con v ZD AdjVm p = 0.0002, Fisher's LSD).

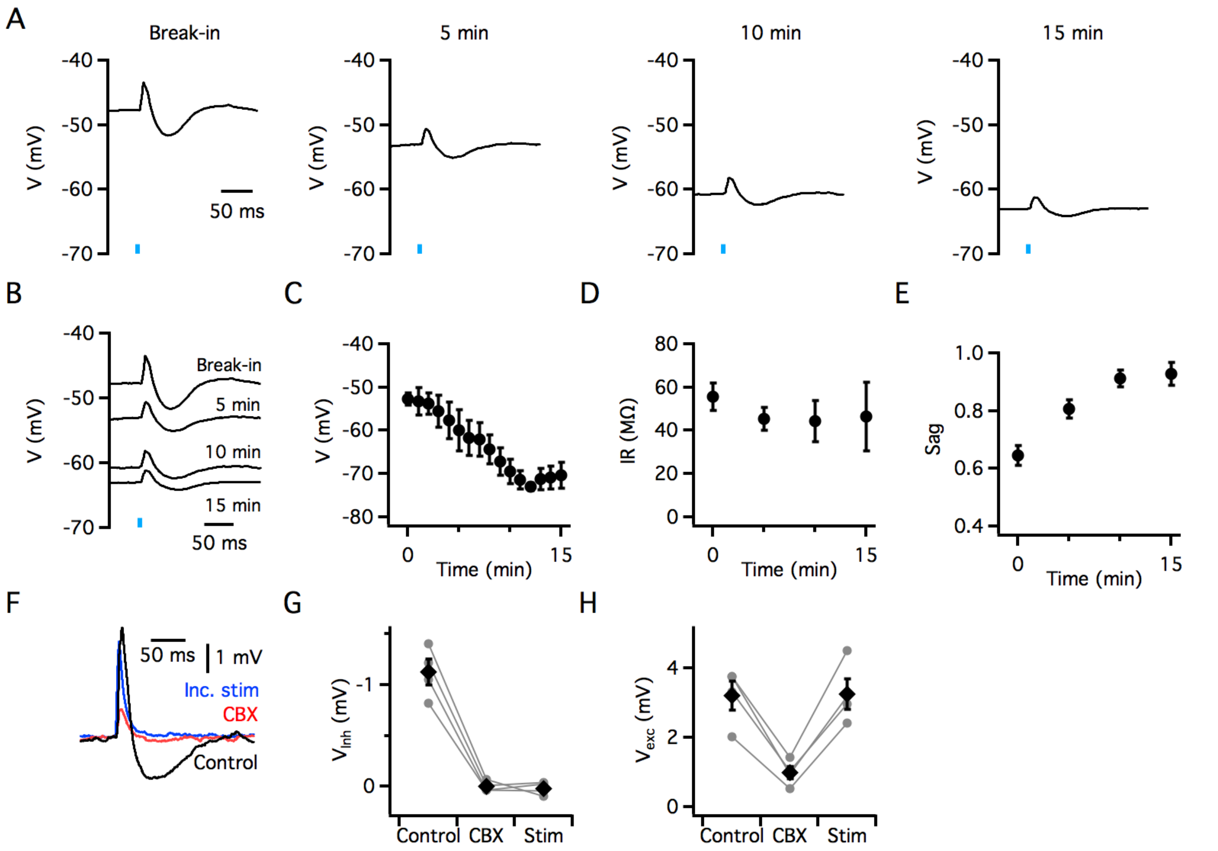

Figure S3. Change in intrinsic membrane properties during intracellular dialysis with ZD7288 (relates to Figure 3)

(A) Examples of light-evoked synaptic potentials recorded from a Thy1-ChR2 mouse at different times after break in.

(B) Data from (A) replotted to illustrate the change in membrane potential during intracellular dialysis with ZD7288.

(C-E) Mean resting membrane potential (C) ($F_{3,12}$ = 17.7, p = 0.0001, one-way repeated measures ANOVA, 0 min v 15 min p = 0.0005, Fisher's LSD, n = 5) , input resistance (D) ($F_{3,12}$ = 1.1, p = 0.40, one-way repeated measures ANOVA, n =5) and membrane potential sag response to current steps (E) ($F_{3,12}$ = 27.7, p = 1.1 x 10^{-5}, one-way repeated measures ANOVA, 0 min v 15 min p = 2.6 x 10^{-6}) plotted as a function of time since break in.

(F) Examples of light-evoked synaptic potentials recorded from a Thy1-ChR2 mouse in control conditions, during perfusion of carbenoxolone and then with the stimulus intensity increased.

(G-H) In the presence of carbenoxolone the amplitude of the depolarizing component of the glutamatergic synaptic response (control 3.5 ± 0.2 mV, carbenoxolone 1.3 ± 0.3, p =

0.0007, n = 4, paired t-test)(G) was reduced and the hyperpolarizing component was completely abolished ($p = 0.0008$, n = 4, paired t-test)(H). Carbenoxolone was bath applied for 30 minutes, also resulting in a moderate increase in input resistance (control 21.1 ± 4.9 MΩ, carbenoxolone 32.5 ± 9.1 MΩ, $p = 0.33$, n = 4, paired t-test) without any change in membrane potential (control -53.49 ± 2.06 mV, carbenoxolone -53.64 ± 0.68 mV, $p = 0.95$, n = 4, paired t-test).

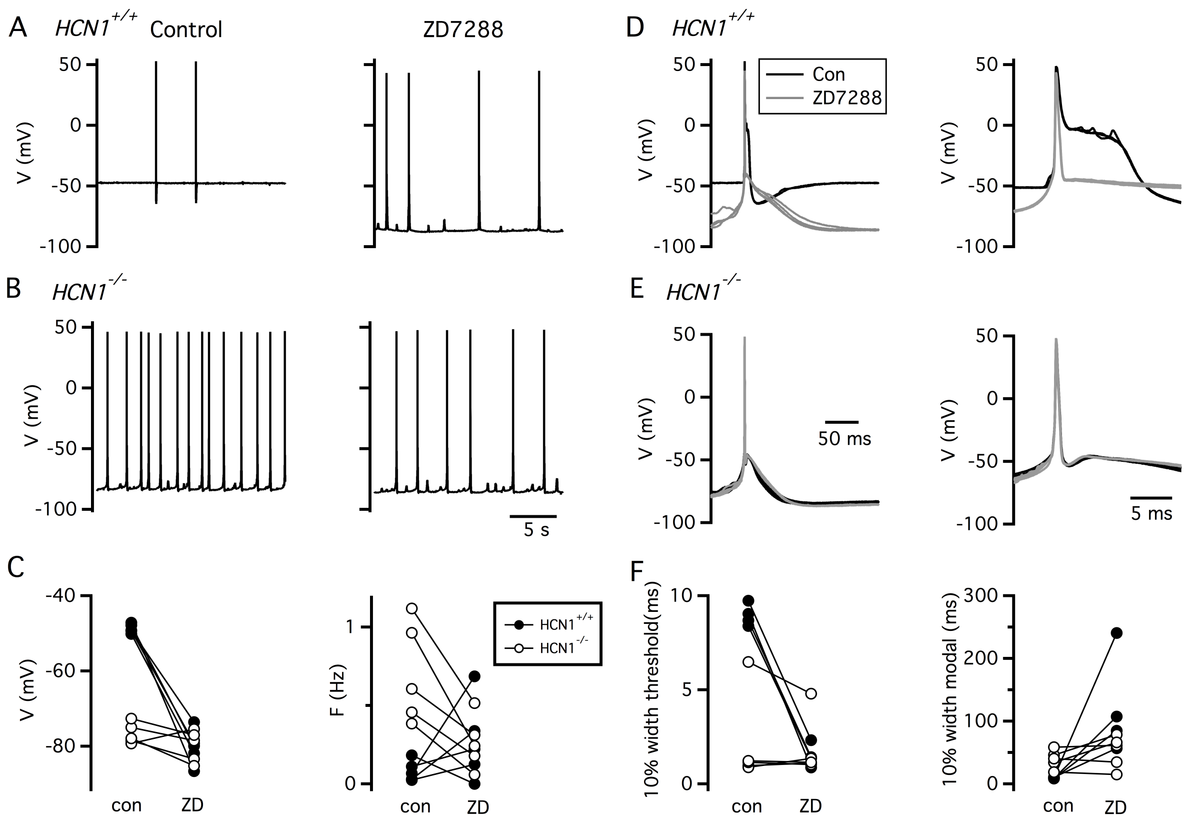

Figure S4. Effects of HCN1 deletion on action potential firing are reproduced by pharmacological block of I_h (relates to Figures 4 and 5)

(A-B) Examples of membrane potential of neurons *HCN1*$^{+/+}$ mice (A) and *HCN1*$^{-/-}$ mice (B) recorded in control conditions (left) and subsequently during perfusion of ZD7288 (right).

(C) Membrane potential ($F_{1,16}$ = 123.1 p = 6.36 x 10^{-9} for effect of ZD7288, $F_{1,16}$ = 81.6 p = 1.1 x 10^{-7} for interaction between genotype and ZD7288, one way repeated measures ANOVA, *HCN1*$^{+/+}$ Con v ZD p = 1.0 x 10^{-7} *HCN1*$^{-/-}$ Con v ZD p = 0.48, Tukey's HSD, n = 5), spike frequency ($F_{1,16}$ = 1.3 p = 0.28 for effect of ZD7288, $F_{1,16}$ = 11.6 p = 0.004 for interaction between genotype and ZD7288, one way repeated measures ANOVA, *HCN1*$^{+/+}$ Con v ZD p = 0.32 *HCN1*$^{-/-}$ Con v ZD p = 0.033, Tukey's HSD, n = 5) and coefficient of variation ($F_{1,16}$ = 8.8 p = 0.01 for effect of ZD7288, $F_{1,16}$ = 7.0 p = 0.019 for interaction between genotype and ZD7288, one way repeated measures ANOVA, *HCN1*$^{+/+}$ Con v ZD p = 0.09 *HCN1*$^{-/-}$ Con v ZD p = 0.68, Tukey's HSD, n = 5) for all neurons from *HCN1*$^{+/+}$ mice (closed circles) and *HCN1*$^{-/-}$ mice (open circles) in control conditions (con) and during subsequent perfusion of ZD7288 (ZD)

(D-E) Waveforms of consecutive spontaneous action potentials recorded from *HCN1*$^{+/+}$ (D) and *HCN1*$^{-/-}$ IO neurons (E) recorded in control conditions and during subsequent perfusion of ZD7288. Waveforms to the right show the spikes on a 10x expanded time scale.

(F) Width of the spike (left) ($F_{1,16}$ = 20.7 p = 0.0004 for effect of ZD7288, $F_{1,16}$ = 22.3 p = 0.0003 for interaction between genotype and ZD7288, one way repeated measures ANOVA, *HCN1*$^{+/+}$ Con v ZD p = 6.7 x 10^{-5} *HCN1*$^{-/-}$ Con v ZD p = 0.99, Tukey's HSD, n = 5), and the spike complex (right) ($F_{1,16}$ = 8.6 p = 0.011 for effect of ZD7288, $F_{1,16}$ = 6.8 p = 0.021 for interaction between genotype and ZD7288, one way repeated measures ANOVA, *HCN1*$^{+/+}$ Con v ZD p = 0.008 *HCN1*$^{-/-}$ Con v ZD p = 0.97, Tukey's HSD, n = 5), measured as in Figure 6, for all neurons perfused with ZD7288, labelled as in (C).

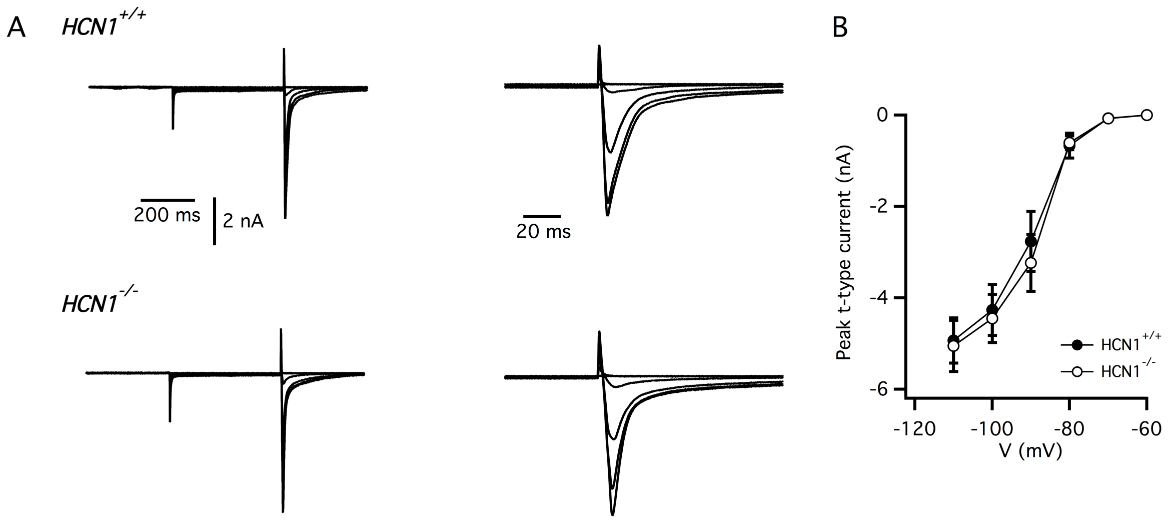

Figure S5. Deletion of HCN1 does not cause adaptation of T-type currents in IO neurons (relates to Figure 4)

(A) Examples of T-type currents recorded from IO neurons from *HCN1*$^{+/+}$ (top) and *HCN1*$^{-/-}$ mice (bottom).

(B) The mean peak amplitude of isolated T-type currents is plotted as a function of membrane potential for IO neurons from *HCN1*$^{+/+}$ and *HCN1*$^{-/-}$ mice. There was no significant difference in the maximal tail current amplitude (p=0.79, n=5, t-test).

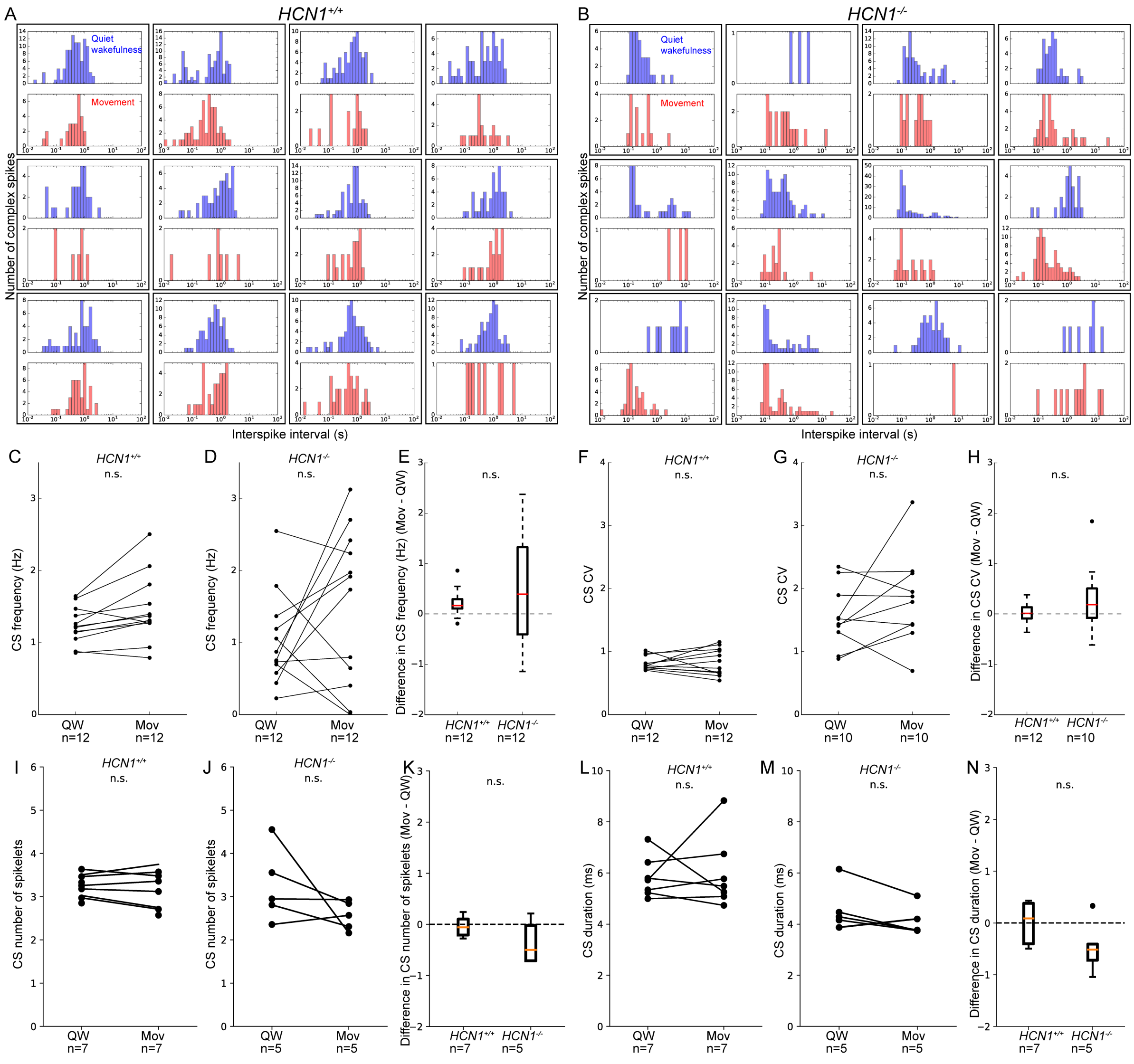

Figure S6. Complex spike properties during quiet wakefulness and movement (relates to Figure 6)

(A - B) Histograms of ISI distributions of individual cells from *HCN1+/+* mice (A) and from *HCN1-/-* mice (B), during quiet wakefulness (blue) and movement (red). Each black box indicates data for one Purkinje cell.

(C - D) Absolute values of complex spike frequency during quiet wakefulness and movement in *HCN1+/+* mice (C) and *HCN1-/-* mice (D).

(E) Difference in complex spike frequency between periods of movement and periods of quiet wakefulness.

(F - G) Absolute values of complex spike CV during quiet wakefulness and movement in *HCN1+/+* mice (F) and *HCN1-/-* mice (G).

(H) Change in complex spike CV during periods of movement when compared to periods of quiet wakefulness.

(I-J) Absolute values of the number of CS spikelets during quiet wakefulness and movement in *HCN1*$^{+/+}$ mice (I) and *HCN1*$^{-/-}$ mice (J).

(K) Difference in the number of CS spikelets between movement and quiet wakefulness.

(L-M) The duration of complex spikes recorded during quiet wakefulness and movement from *HCN1*$^{+/+}$ mice (L) and *HCN1*$^{-/-}$ mice (M).

(N) Difference in the CS duration between movement and quiet wakefulness.

	Quiet wakefulness		Movement	
	HCN1$^{+/+}$	*HCN1*$^{-/-}$	*HCN1*$^{+/+}$	*HCN1*$^{-/-}$
**CS frequency (Hz)**	1.21 ± 0.06 (n=21)	0.87 ± 0.14 (n=17)	1.46 ± 0.14 (n = 12)	1.50 ± 0.31 (n = 12)
**Difference in mean**[a]	*U* = 72, p = 0.002		*U* = 68, p = 0.840	
**Difference in distribution**[b]	*D* = 0.599, p = 0.001		*D* = 0.333, p = 0.433	
**CS CV**	0.79 ± 0.02 (n=21)	1.48 ± 0.13 (n=17)	0.83 ± 0.06 (n = 12)	1.83 ± 0.23 (n = 12)
**Difference in mean**[a]	*U* = 27, p = 0.000		*U* = 7, p = 0.001	
**Difference in distribution**[b]	*D* = 0.7507, p = 0.000		*D* = 0.9000, p = 0.000	
**Number of spikelets**	3.24 ± 0.08 (n=14)	3.37 ± 0.21 (n = 10)	3.20 ± 0.16 (n = 7)	2.56 ± 0.15 (n = 5)
**Difference in mean***	*U* = 61, p = 0.828		*U* = 4, p = 0.034	
**Difference in distribution**[b]	*D* = 0.3571, p = 0.363			
**CS duration (ms)**	5.81 ± 0.20 ms (n = 14)	6.14 ± 0.88 ms (n = 10)	5.99 ± 0.53 (n = 7)	4.11 ± 0.26 (n = 5)
**Difference in mean**[a]	*U* = 59, p = 0.734		*U* = 2, p = 0.014	
**Difference in distribution**[b]	*D* = 0.400, p = 0.237			
**SS frequency (Hz)**	54.50 ± 6.71 (n=21)	49.41 ± 3.72 (n=17)	67.10 ± 7.65 (n = 12)	62.89 ± 7.99 (n = 12)
**Difference in mean**[a]	*U* = 132, p = 0.255		*U* = 64, p = 0.665	
**Difference in distribution**[b]	*D* = 0.252, p = 0.525		*D* = 0.167, p = 0.991	

[a] Mann-Whitney U test
[b] Kolmogorov-Smirnov test

Table S1. Properties of complex spikes and simple spikes recorded from Purkinje cells *in vivo* (relates to Figure 6)

Data recorded from Purkinje cells in awake, behaving mice, comparing *HCN1*$^{+/+}$ and *HCN1*$^{-/-}$ mice during periods of quiet wakefulness and periods of movement.

	HCN1$^{+/+}$		*HCN1*$^{-/-}$	
	**Quiet wakefulness**	**Movement**	**Quiet wakefulness**	**Movement**
**CS frequency (Hz)**	1.24 ± 0.07 (n = 12)	1.46 ± 0.14 (n = 12)	1.02 ± 0.19 (n = 12)	1.50 ± 0.31 (n = 12)
**Difference in mean**[a]	*U* = 47, p = 0.158		*U* = 56, p = 0.370	
**Difference in distribution**[b]	*D* = 0.333, p = 0.433		*D* = 0.4167, p = 0.186	
**Difference in CS frequency (Mov – QW)**	0.22 ± 0.08 (n = 12)		0.48 ± 0.34 (n = 10)	
**Difference in mean**[a]	*U* = 65, p = 0.707			
**CS CV**	0.81 ± 0.03 (n = 12)	0.83 ± 0.06 (n = 12)	1.55 ± 0.16 (n = 10)	1.84 ± 0.23 (n = 10)
**Difference in mean**[a]	*U* = 67, p = 0.796		*U* = 40, p = 0.472	
**Difference in distribution**[b]	*D* = 0.4167, p = 0.186		*D* = 0.300, p = 0.675	
**Difference in CS CV (Mov – QW)**	0.019 ± 0.057 (n = 12)		0.28 ± 0.22 (n = 10)	
**Difference in mean**[a]	*U* = 47, p = 0.410			
**CS number of spikelets**	3.24 ± 0.10 (n = 7)	3.20 ± 0.16 (n = 7)	3.24 ± 0.38 (n = 5)	2.56 ± 0.15 (n = 5)
**Difference in mean**[a]	*U* = 24, p = 1.00		*U* = 20, p = 0.144	
**Difference in CS number of spikelets (Mov – QW)**	-0.044 ± 0.076 (n = 7)		-0.68 ± 0.46 (n = 5)	
**Difference in mean**[a]	*U* = 25, p = 0.256			
**CS duration**	5.83 ± 0.30 (n = 7)	5.99 ± 0.53 (n = 7)	4.59 ± 0.40 (n = 5)	4.11 ± 0.26 (n = 5)
**Difference in mean**[a]	*U* = 26, p = 0.90		*U* = 19, p = 0.210	
**Difference in CS duration (Mov – QW)**	0.16 ± 0.59 (n = 7)		-0.47 ± 0.23 (n = 5)	
**Difference in mean**[a]	*U* = 26, p = 0.194			

[a] Mann-Whitney U test
[b] Kolmogorov-Smirnov test

Table S2. Properties of complex spike firing compared between periods of quiet wakefulness and periods of movement in *HCN1*$^{+/+}$ and *HCN1*$^{-/-}$ mice (relates to Figure 6)

Data recorded from Purkinje cells in awake, behaving mice, comparing periods of quiet wakefulness and periods of movement in *HCN1*$^{+/+}$ and *HCN1*$^{-/-}$ mice. Only cells that had both a period of quiet wakefulness and a period of movement were taken into account. Statistical comparisons correspond to the data shown in Figure S6(C-N).
