# Supplementary material for: Inferior Olive HCN1 Channels Coordinate Synaptic Integration and Complex Spike Timing
Source: Cell Rep. 2018 Feb 13;22(7):1722–33. doi: 10.1016/j.celrep.2018.01.069 (PMC5847187; doi:10.1016/j.celrep.2018.01.069)
Supplement: Document S2. Article plus Supplemental Information [file mmc2.pdf]

Cell Reports

Article

Inferior Olive HCN1 Channels Coordinate Synaptic Integration and Complex Spike Timing

Graphical Abstract

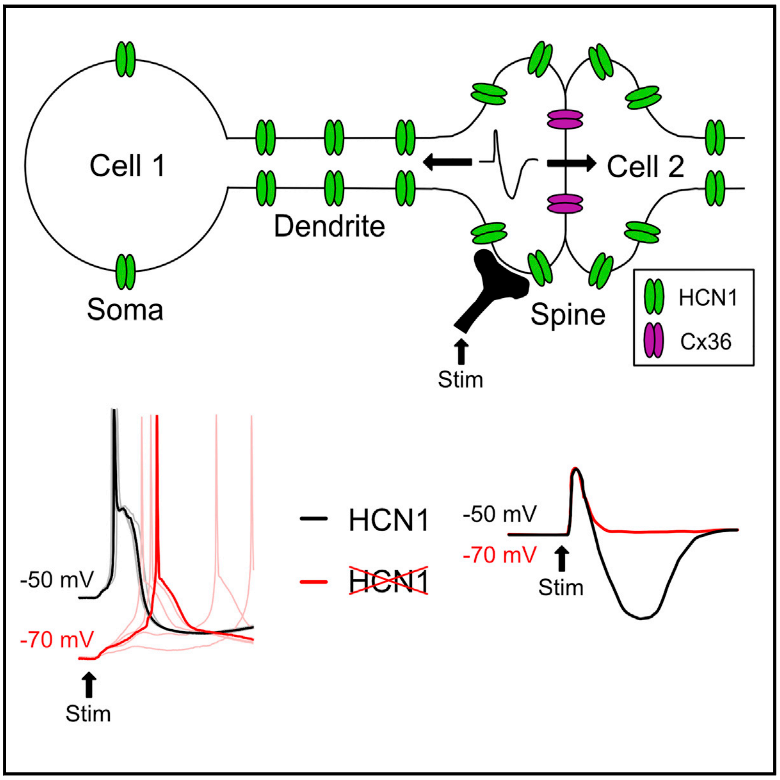

Highlights

- HCN1 channels in IO neurons control synaptic response and spiking activity
- Network actions of HCN1 channels enable bidirectional synaptic responses
- Local actions of HCN1 channels control spike timing and spikelet number
- In awake mice, HCN1 channels reduce timing variability of cerebellar complex spikes

Authors

Derek L.F. Garden, Marlies Oostland, Marta Jelitai, Arianna Rinaldi, Ian Duguid, Matthew F. Nolan

Correspondence

mattnolan@ed.ac.uk

In Brief

Garden et al. show that in the IO, distinct network-wide and local actions of HCN1 channels, respectively, determine the waveform of synaptic potentials and timing of spike initiation. Thus, spatially distributed actions of HCN1 signaling may support roles of the IO in motor coordination.

Garden et al., 2018, Cell Reports *22*, 1722–1733
February 13, 2018 © 2018 The Authors.
https://doi.org/10.1016/j.celrep.2018.01.069

CellPress

OPEN ACCESS
CellPress

Cell Reports
Article

Inferior Olive HCN1 Channels Coordinate Synaptic Integration and Complex Spike Timing

Derek L.F. Garden,[1,3] Marlies Oostland,[1,3] Marta Jelitai,[1] Arianna Rinaldi,[1] Ian Duguid,[1,2] and Matthew F. Nolan[1,2,4,*]
[1]Centre for Discovery Brain Sciences, University of Edinburgh, Edinburgh EH8 9XD, UK
[2]Simons Initiative for the Developing Brain, University of Edinburgh, Edinburgh EH8 9XD, UK
[3]These authors contributed equally
[4]Lead Contact
*Correspondence: mattnolan@ed.ac.uk
https://doi.org/10.1016/j.celrep.2018.01.069

SUMMARY

Cerebellar climbing-fiber-mediated complex spikes originate from neurons in the inferior olive (IO), are critical for motor coordination, and are central to theories of cerebellar learning. Hyperpolarization-activated cyclic-nucleotide-gated (HCN) channels expressed by IO neurons have been considered as pacemaker currents important for oscillatory and resonant dynamics. Here, we demonstrate that *in vitro*, network actions of HCN1 channels enable bidirectional glutamatergic synaptic responses, while local actions of HCN1 channels determine the timing and waveform of synaptically driven action potentials. These roles are distinct from, and may complement, proposed pacemaker functions of HCN channels. We find that in behaving animals HCN1 channels reduce variability in the timing of cerebellar complex spikes, which serve as a readout of IO spiking. Our results suggest that spatially distributed actions of HCN1 channels enable the IO to implement network-wide rules for synaptic integration that modulate the timing of cerebellar climbing fiber signals.

INTRODUCTION

Theories of motor learning propose critical roles for the timing of cerebellar complex spikes, which originate from neurons in the inferior olive (IO) (Albus, 1970; De Zeeuw et al., 2011; Marr, 1969). This is supported by evidence that the frequency and timing of IO action potentials instructs the amplitude and direction of synaptic plasticity in the cerebellar cortex (Mathy et al., 2009). Thus, mechanisms that control the timing of spike output from the IO may play key roles in cerebellar-dependent motor coordination and learning.

Spike timing emerges from dynamic interactions between synaptic activity and intrinsic neuronal excitability (Dayan and Abbott, 2001). Neurons in the IO are striking in that intrinsic excitability appears to have a powerful influence on these dynamics. Spontaneous sinusoidal subthreshold membrane potential oscillations and membrane potential resonance emerge from interactions between multiple ion channel types (Benardo and Foster, 1986; Llinás and Yarom, 1981a, 1981b; Matsumoto-Makidono et al., 2016). Excitability of neurons in the IO also influences integration of synaptic inputs, with glutamatergic inputs to neurons in the IO generating distinct bidirectional synaptic potentials through recruitment of calcium-activated potassium channels (Garden et al., 2017). Gap-junction-mediated electrical synaptic connections between IO neurons synchronize oscillatory activity (Bal and McCormick, 1997; De Zeeuw et al., 1998; Llinas et al., 1974; Long et al., 2002) and have been proposed also to coordinate synaptic integration (Kistler and De Zeeuw, 2005). These distinctive excitable properties have motivated suggestions that the IO has unique computational roles within the brain (De Zeeuw et al., 1998; Welsh and Llinás, 1997). Nevertheless, the extent to which intrinsic excitability of IO neurons influences spike timing in behaving animals is unclear.

Excitability is determined at a molecular level by the set of ion channels that a neuron expresses. Of particular interest are the hyperpolarization-activated cyclic-nucleotide-gated (HCN) family of ion channels, which mediate hyperpolarization-activated currents (I_h) that contribute to pacemaking and integrative properties of many central neurons (Robinson and Siegelbaum, 2003). HCN channels in the IO are suggested to act as pacemakers of oscillatory activity and mediate membrane potential resonance (Bal and McCormick, 1997; Matsumoto-Makidono et al., 2016). In contrast, their impact on synaptic integration in the IO is unclear. The HCN1 subunit is highly expressed in the IO and cerebellar cortex (Notomi and Shigemoto, 2004; Santoro et al., 2000). Global deletion of HCN1 channels causes deficits in learned motor behaviors (Nolan et al., 2003). While impairments in later stages of motor learning can in part be accounted for by contributions of HCN1 channels to synaptic integration in cerebellar Purkinje cells (Rinaldi et al., 2013), it is not clear whether HCN channels in upstream neurons influence activity in the cerebellar cortex. Here, we asked whether HCN1 channels in the IO affect synaptic integration as well as membrane potential resonance and oscillations and whether the contribution of HCN1 channels to excitability in the IO affects action potential firing in behaving animals.

We demonstrate that HCN1 channels mediate I_h in IO neurons, are required for the inhibitory component of responses to glutamatergic synaptic inputs, and oppose temporal summation of subthreshold inputs while also controlling the timing and waveform of spike output. Whereas the suprathreshold actions of HCN1 rely on local depolarization of the somatic membrane potential, generation of inhibitory components of synaptic responses involves network actions of HCN1 mediated by electrical connections with other IO neurons. We find that genetic deletion of

 © 2018 The Authors.
This is an open access article under the CC BY license (http://creativecommons.org/licenses/by/4.0/).

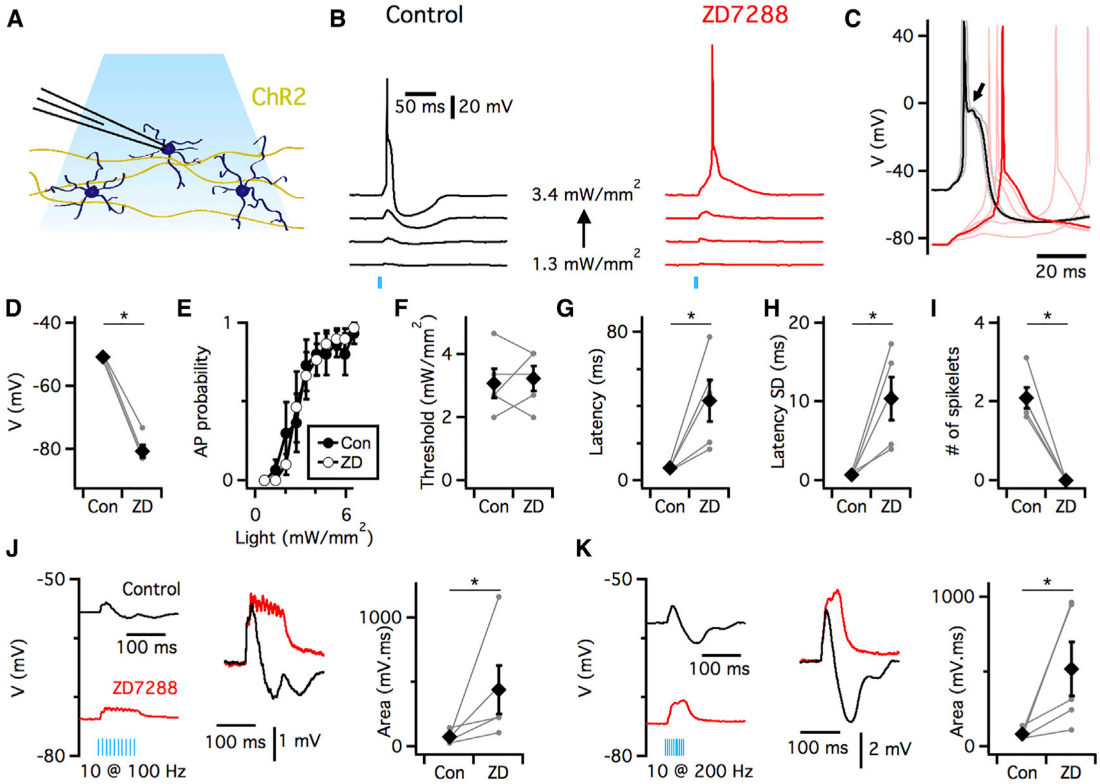

Figure 1. Temporal Integration by IO Neurons Is Modified by Block of HCN Channels

(A) Whole-cell recordings were made from neurons in the IO. ChR2 was expressed in axons that project into the IO, but not in IO neurons.

(B) Example responses to increasing intensity of light stimulation (vertical bar, 1.3–3.4 mW/mm^2) of IO neurons from Thy1-ChR2 mice before (left) and during (right) application of 10 μM ZD7288. In the presence of ZD7288, the excitatory component of the subthreshold response is maintained (control: 2.63 ± 0.65 mV, ZD7288: 1.52 ± 0.60 mV, p = 0.081, n = 5, paired t test), but the hyperpolarizing component is abolished (control: −2.72 ± 0.95 mV, ZD7288: −0.04 ± 0.02 mV, p = 0.049, n = 5, paired t test).

(C) Suprathreshold responses from (B) on an expanded timescale. Solid trace shows response with median latency, while lighter traces show additional responses to the same intensity of stimulation for each condition. Arrow indicates spikelets.

(D) Resting membrane potential is more negative during (ZD) compared with before (Con) perfusion of 10 μM ZD7288 ($p = 5.08 \times 10^{-5}$, n = 5, paired t test). Individual data points are shown as filled circles and mean values as black diamonds.

(E) Perfusion of ZD7288 does not affect the probability of spike firing as a function of stimulus intensity ($F_{1,70}$ = 0.004, p = 0.96, two-way repeated-measures ANOVA, n = 5).

(F–I) Comparison before (Con) and during application of ZD7288 (ZD) of the mean light threshold for AP firing (p = 0.82, n = 5, paired t test) (F), the spike latency at the threshold stimulus intensity (p = 0.01, n = 5, paired t test) (G), the SD of the spike latency at the threshold stimulus intensity (p = 0.02, n = 5, paired t test) (H), and the number of spikelets (p = 0.0014, n = 5, paired t test) (I).

(J and K) Example responses to trains of 10 stimuli at 100 Hz (J) and 200 Hz (K) before and during application of ZD7288 (left). ZD7288 increases the area of the depolarization (100 Hz: p = 0.02, 200 Hz: $p = 5.14 \times 10^{-3}$, paired t test).

Error bars in (D)–(K) indicate SEM.

HCN1 increases variability in the timing of complex spikes recorded from cerebellar Purkinje cells during quiet wakefulness and movement. Thus, HCN1 channels within the IO have multiple spatially distributed actions that may impact motor coordination by influencing the pattern of cerebellar complex spike activity.

RESULTS

Pharmacological Block of I_h Modifies Excitability and Synaptic Integration

To investigate whether I_h influences excitability and synaptic integration by IO neurons, we first examined actions of the HCN channel blocker ZD7288. We made patch-clamp recordings in brain slices from IO principal neurons identified by their large soma and characteristic action potential after depolarization (Llinás and Yarom, 1981b). To investigate synaptic responses, we used mice that express channelrhodopsin 2 (ChR2) and enhanced yellow fluorescent protein (eYFP) under control of the Thy1 promoter (Arenkiel et al., 2007). In these mice, activation of ChR2 with low intensities of 480-nm light reliably evoked bidirectional glutamatergic postsynaptic potentials (PSPs) in IO neurons (Garden et al., 2017) (Figures 1A and 1B). At higher intensities, synaptic excitation triggered action potentials and associated spikelets (Figures 1B and 1C).

We found that perfusion of ZD7288 hyperpolarized IO neurons by ~30 mV ($p = 5.08 \times 10^{-5}$, n = 5, paired t test) (Figure 1C and 1D). The inhibitory component of the subthreshold PSP was abolished by ZD7288 (~1% of control amplitude, p = 0.049, paired t test), while the excitatory component was maintained (~ 55% of control amplitude, p = 0.081, paired t test) (Figure 1B). Surprisingly, given the large change in membrane potential, block of I_h did not affect the relationship between stimulus intensity and spike probability ($F_{1,70}$ = 0.004 p = 0.96, two-way repeated-measures ANOVA) (Figure 1E) or the threshold light intensity required to trigger a spike (p = 0.82, paired t test) (Figure 1F). However, the mean and SD of the latency of the spike response were increased more than 5-fold by ZD7288 (p = 0.01 and p = 0.02 respectively, paired t test) (Figures 1C, 1G, and 1H), while spikelets associated with the action potentials were abolished (p = 0.0014, paired t test) (Figures 1C and 1I).

We also examined subthreshold temporal summation in response to trains of synaptic input activated at 100 and 200 Hz (Figures 1J and 1K). In control conditions, trains at either stimulation frequency caused an initial rapid depolarization that reached a maximal amplitude within ~ 16 ms of stimulation, followed by a hyperpolarization that peaked within ~70 ms of stimulation (see also Garden et al., 2017; Turecek et al., 2014). Following block of I_h with ZD7288 the hyperpolarizing response was abolished, while the amplitude and duration of the depolarizing component were increased. These changes were also reflected in a large increase in the area of the synaptic responses (100 Hz: p = 0.02, n = 5; 200 Hz, $p = 5.14 \times 10^{-3}$, n = 4, paired t test).

Together, these data indicate that pharmacological block of I_h increases the latency and variability in the timing of synaptically driven action potentials, prevents associated spikelets, abolishes inhibitory components of subthreshold synaptic inputs, and increases temporal summation of subthreshold responses. Thus, I_h may be a major determinant of the way IO neurons respond to synaptic input, with roles that appear distinct from its known functions in other neuron types.

HCN1 Channels Enable the Hyperpolarizing Component of Long-Range Synaptic Responses

Do HCN1 channels in IO neurons mediate the integrative roles of I_h suggested by pharmacological manipulation? To address this, we used mice in which the *HCN1* gene is deleted (Nolan et al., 2003). Comparison of HCN1 knockout (*HCN1*$^{-/-}$) mice with control (*HCN1*$^{+/+}$) mice demonstrated that HCN1 channels mediate the prominent I_h recorded from IO neurons and that deletion of HCN1 causes similar changes in resting properties of IO neurons to pharmacological block of I_h (Figure S1). These data are largely consistent with and extend recent observations of changes in membrane currents and integrative properties of IO neurons in *HCN1*$^{-/-}$ mice (Matsumoto-Makidono et al., 2016). We therefore went on to address the influence of HCN1 channels on subthreshold PSPs by comparing responses to excitatory synaptic input of IO neurons from *HCN1*$^{+/+}$ and *HCN1*$^{-/-}$ mice.

To evaluate responses to glutamatergic synaptic inputs in *HCN1*$^{+/+}$ and *HCN1*$^{-/-}$ mice, we used slices from mice in which adeno-associated virus (AAV) expressing ChR2 was injected into the motor cortex (Garden et al., 2017). In these experiments, we first compared responses of each genotype when neurons were at their resting membrane potential (Figures 2A and 2D–2F). Because deletion or block of HCN1 channels causes a large hyperpolarization of the resting membrane potential of IO neurons, and because this might be expected to modify the driving force for synaptic currents and gating of other voltage-gated ion channels, we also investigated whether the waveform of synaptic responses differed when compared at similar membrane potentials (Figures 2B and 2C). Thus, neurons from *HCN1*$^{+/+}$ mice were hyperpolarized to −80 mV by injection of negative current (Figure 2B), whereas neurons from *HCN1*$^{-/-}$ mice were depolarized to −50 mV by injection of positive current (Figure 2C).

Whereas in IO neurons from HCN1$^{+/+}$ mice the inhibitory components of the response to neocortical input was observed at resting potential and at −80 mV (Figures 2A, 2B, 2E, and 2G), it was completely absent at both test potentials in IO neurons from HCN1$^{-/-}$ mice ($F_{1,16}$ = 110.8, $p = 1.34 \times 10^{-8}$ for effect of genotype, ANOVA, n = 5) (Figures 2C–2E and 2G). In addition, while initial excitatory responses were present in neurons from both groups of mice, their waveform differed. Excitatory responses from HCN1$^{+/+}$ mice had two or more peaks, whereas for IO neurons from HCN1$^{-/-}$ mice, they had only a single peak (Figure 2F). In the absence of HCN1, the amplitude of the first peak was slightly larger ($F_{1,16}$ = 5.6, p = 0.03, ANOVA), while the maximum amplitude was reduced ($F_{1,16}$ = 12.2, p = 0.003, ANOVA) and the overall duration of the excitatory component was shorter ($F_{1,16}$ = 34.7 $p = 2.29 \times 10^{-5}$, ANOVA) (Figure 2G–2J). Changing the membrane potential between −50 mV and −80 mV had relatively little effect on the amplitude of the excitatory ($F_{1,16}$ = 0.006, p = 0.94 for effect of membrane potential on the first peak and $F_{1,16}$ = 0.34, p = 0.57 for maximum amplitude, ANOVA, n = 5) or the inhibitory component of the PSP ($F_{1,16}$ = 2.5, p = 0.13). We obtained similar results using the blocker ZD7288, indicating that the dependence of inhibitory potentials on HCN1 channels is a direct result of the absence of *HCN1* rather than a secondary adaptation following gene deletion (Figure S2).

Together, these data indicate that direct activation of HCN1 channels is required for inhibitory components of IO responses to long-range glutamatergic inputs and controls the waveform of excitatory components. Because hyperpolarization of the resting potential of neurons from *HCN1*$^{+/+}$ mice did not replicate, and depolarization of neurons from *HCN1*$^{-/-}$ mice did not rescue, the effects of *HCN1* deletion (cf. Figures 2A–2D and 2G–2J), the requirement of HCN1 channels for the inhibitory component of the GluA synaptic responses of IO neurons is independent of their actions on the somatic membrane potential.

HCN1 Channels in Adjoining Electrically Coupled IO Neurons Are Sufficient for Generation of the Inhibitory Component of Glutamatergic Synaptic Responses

Excitatory synapses in the IO are organized in glomeruli, which may mediate interactions between adjacent postsynaptic neurons. Each glomerulus contains up to 8 dendritic spines that originate from different IO neurons and are connected to one another by gap junctions (de Zeeuw et al., 1990) (Figure 3A). A theoretical model of synaptic integration in the IO predicts that excitatory input will trigger local spikes that propagate between spines

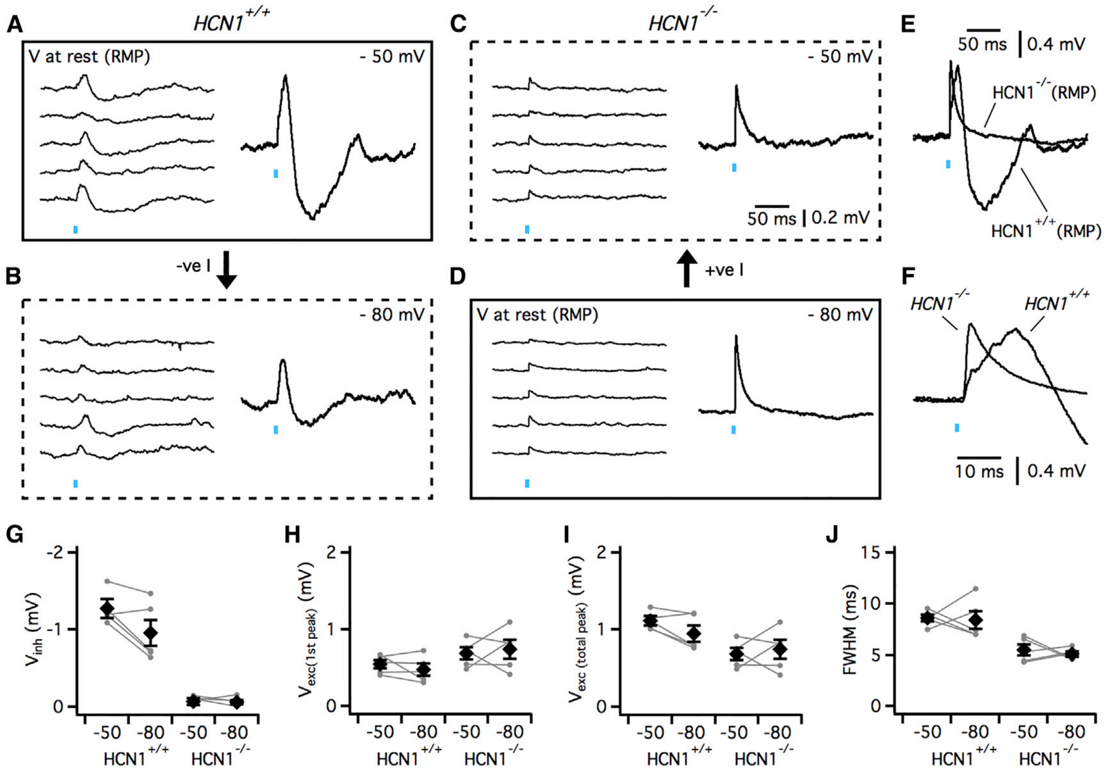

Figure 2. HCN1 Channels Control the Waveform of Responses to Long-Range Synaptic Input

(A–D) Example responses of IO neurons from $HCN^{+/+}$ (A and B) and $HCN^{-/-}$ mice (C and D) to optical activation of neocortical inputs recorded with membrane potential at −50 mV (A and C) or −80 mV (B and D). The same stimulus is repeated 5 times. Mean responses are shown to the right of individual traces. Boxes constructed from solid lines indicate responses recorded at the resting potential and arrows indicate direction of injected current.

(E and F) Responses from (A) and (D) on a faster timescale illustrate differences at the resting membrane potential of the inhibitory (E) and excitatory (F) components of PSPs.

(G–J) Comparison between $HCN^{+/+}$ and $HCN^{-/-}$ mice of the amplitude of the inhibitory component (genotype: $F_{1,16} = 110.8$ $p = 1.34 \times 10^{-8}$; membrane potential: $F_{1,16} = 2.50$ $p = 0.13$) (G), the amplitude of the first peak of the excitatory component (genotype: $F_{1,16} = 5.64$ $p = 0.03$; membrane potential: $F_{1,16} = 0.006$ $p = 0.94$) (H), the maximal amplitude of the excitatory component (genotype: $F_{1,16} = 12.2$ $p = 0.003$; membrane potential: $p = 0.57$) (I), and the width of the excitatory component at its half maximum amplitude (FWHM) (genotype: $F_{1,16} = 34.7$ $p = 2.29 \times 10^{-5}$; membrane potential: $F_{1,16} = 0.26$ $p = 0.62$) (J). All comparisons use a two-way ANOVA (n = 5). Individual data points are shown as filled circles and mean values as diamonds. Error bars indicate SEM.

within a glomerulus via gap junctions and that will appear as bidirectional responses at the soma of each neuron (Kistler and De Zeeuw, 2005). Our observation of bidirectional glutamatergic synaptic responses that are relatively insensitive to somatic membrane potential is consistent with predictions of this model (Figure 2; Garden et al., 2017). We therefore reasoned that the actions of HCN1 channels on synaptic responses may in part originate from neurons electrically connected to the recorded cell. In this case, we expect that whereas block of I_h in all connected neurons will abolish the inhibitory component of synaptic responses (Figures 1 and 2), block of I_h in only the recorded cell will not (cf. Figure 3A). In addition, the inhibitory component might also be sensitive to block of gap junction connections between IO neurons. We therefore set out to test these predictions.

We used intracellular delivery of ZD7288 to block HCN1 channels in the recorded neuron without affecting HCN1 channels in other cells in the network (Figures 3A–3E). Over the first 10 minutes of recording with ZD7288 included in the intracellular solution, the membrane potential of IO neurons hyperpolarized and sag responses were abolished, indicating block of I_h (Figures S3A–S3E). No further change in membrane potential or sag was observed, indicating that block of I_h in the recorded neuron was complete. In parallel with the hyperpolarization of the membrane potential, the amplitude of inhibitory (p = 0.033 versus break-in, Fisher's LSD, n = 5), but not excitatory (p = 0.45 versus break in, Fisher's LSD), components of glutamatergic synaptic responses were reduced, but neither component was abolished (Figures 3B–3E). Strikingly, we found that increasing the intensity of synaptic stimulation rescued the inhibitory component of the synaptic response (p = 0.43 versus break-in, Fisher's LSD) (Figures 3B–3E). Therefore, HCN1 channels in the recorded neuron are not necessary for the inhibitory component of synaptic responses. To check whether in this experiment the inhibitory component nevertheless requires HCN1 channels, we subsequently bath applied ZD7288. We again found that the inhibitory component was abolished (p = 0.001 versus break in, Fisher's LSD), suggesting that it reflects a network-wide requirement for HCN1 channels (Figures 3B–3E).

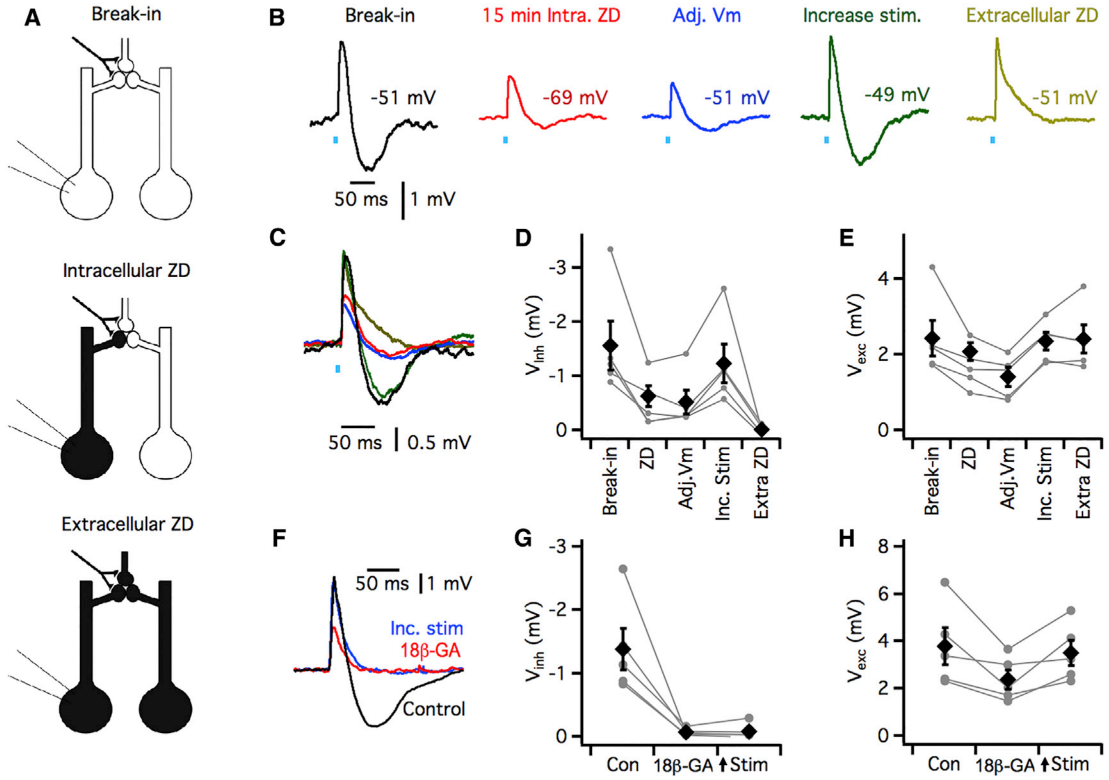

Figure 3. Gap-Junction-Mediated Long-Range Interactions Contribute to HCN1 Channel-Dependent Hyperpolarizing Components of Synaptic Responses

(A) Schematic illustrating glomerular organization of synaptic inputs and strategy for blocking I_h only in the recorded cell (intracellular ZD) and in all cells (extracellular ZD).

(B and C) Examples of synaptic responses recorded from a neuron in the IO of a Thy1-ChR2 mouse with 20 μM ZD7288 included in the intracellular solution. Response are shown in order (B), or overlaid (C), immediately following break-in (black), at 15 minutes following break-in (red), after adjusting the membrane potential to the value at break-in (blue), after increasing the stimulus intensity to restore the amplitude of the depolarizing component (green), and during bath application of 10 μM ZD7288 (yellow).

(D and E) Summary plots of the amplitude of the inhibitory (D) and excitatory (E) components in each condition. Diamonds indicate mean ± SEM and circles indicate individual experiments. The amplitude of inhibitory and excitatory components depended on condition (V_{inh}: $F_{4,16} = 11.2$ $p = 1.59 \times 10^{-4}$; V_{exc}: $F_{4,16} = 6.403$ $p = 0.028$, one-way repeated-measures ANOVA, n = 5). Inhibitory, but not excitatory, components were reduced in amplitude 15 minutes after intracellular perfusion of ZD7288 (p = 0.033 and p = 0.45, respectively, versus break-in, Fisher's LSD), and after adjustment of the membrane potential to its initial level (p = 0.019 and p = 0.039), both components were not significantly different from their break-in value after increasing the stimulus intensity (p = 0.43 and 0.86), and the inhibitory component was abolished in extracellular ZD (p = 0.001 and 0.95).

(F) Examples of synaptic responses recorded from a neuron in the IO of a Thy1-ChR2 mouse in control conditions and then during bath application of the gap junction blocker 18β-glycyrrhetinic acid (18β-GA) (150 μM).

(G and H) The amplitudes of inhibitory (p = 0.0008, paired t test, n = 4) (G) and excitatory (p = 0.0007) (H) components of the synaptic response were reduced in 18β-GA. Diamonds indicate mean ± SEM, and circles indicate individual experiments.

If the inhibitory component of glutamatergic synaptic responses reflects an action of HCN1 distributed across networks of electrically connected IO neurons, then it should also be sensitive to block of gap junctions. To test this possibility, we examined the effects on synaptic responses of bath application of either 18β-glycyrrhetinic acid (18β-GA) (Figures 3F–3H) or carbenoxolone (Figure S3F–S3H), which have both previously been shown to specifically block gap-junction-mediated electrical communication between IO neurons (Leznik and Llinás, 2005; Placantonakis et al., 2006). Application of 18β-GA for 10 min resulted in a moderate increase in input resistance (control 31.8 ± 2.2 MΩ, 18β-GA 37.6 ± 0.7 MΩ, p = 0.15, n = 5, paired t test) without any change in membrane potential (control −53.7 ± 1.9 mV, 18β-GA −54.9 ± 1.5 mV, p = 0.57, n = 5, paired t test). We found that block of gap junctions with 18β-GA reduced the amplitude of the depolarizing component of the glutamatergic synaptic response (p = 0.044, n = 5, paired t test) and completely abolished the hyperpolarizing component (p = 0.017, n = 5, paired t test) (Figures 3F–3H). The hyperpolarizing component remained absent when we increased the stimulus intensity to restore the depolarizing component (Figures 3F–3H). We obtained similar results using carbenoxolone (Figures S3F–S3H). These data support the idea that the HCN1 channels in adjoining electrically connected neurons contribute to the inhibitory component of glutamatergic synaptic responses.

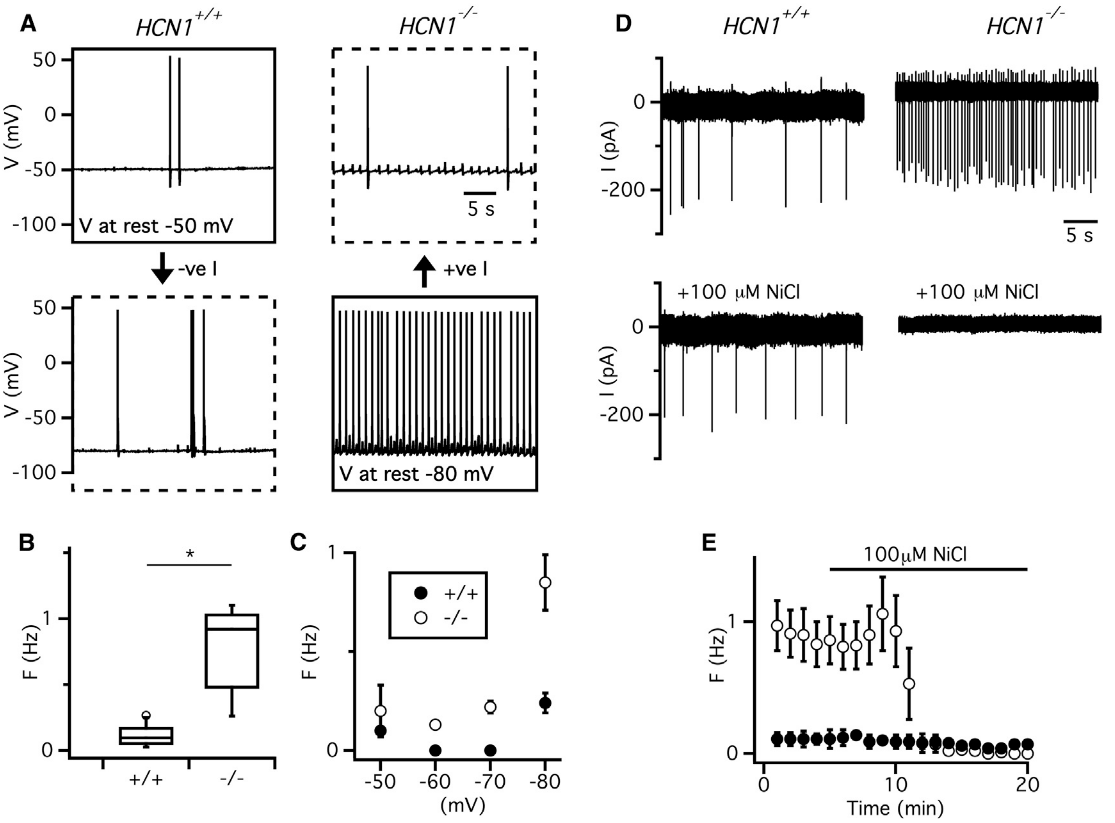

Figure 4. Deletion of HCN1 Increases Spontaneous Action Potential Firing by Recruiting T-Type Ca^{2+} Channels

(A) Membrane potential recordings illustrating spontaneous activity of IO neurons recorded from *HCN1*$^{+/+}$ mice (left) and *HCN1*$^{-/-}$ mice (right). Examples show resting activity (solid line boxes) and activity when the membrane potential is adjusted by injection of current so that in *HCN*$^{+/+}$ mice it is comparable to the resting value from *HCN*$^{-/-}$ mice and vice versa (broken line boxes).

(B) Boxplots of action potential frequency at the resting membrane potential ($p = 7 \times 10^{-5}$, t test, n = 9 in each group).

(C) Plot of spike frequency as a function of membrane potential ($F_{1,56} = 16.4$ p = 0.0001 for effect of genotype; $F_{3,56} = 8.7$ $p = 8.02 \times 10^{-5}$ for interaction between genotype and membrane potential, ANOVA, n = 8 for both groups).

(D) Examples of cell attached recordings from IO neurons from *HCN1*$^{+/+}$ mice (left) and *HCN1*$^{-/-}$ mice (right) in control conditions (upper) and during block of T-type Ca^{2+} channels with Ni^{2+} (lower).

(E) Mean spike frequency versus time for effect of Ni^{2+}. ANOVA indicated a significant effect on spike frequency of genotype ($F_{1,12} = 9.5$ p = 0.0096) and Ni^{2+} ($F_{1,12} = 24.9$ p = 0.0003) and a significant interaction between the two manipulations ($F_{1,12} = 12.9$ p = 0.0037). Post hoc tests indicate that Ni^{2+} has no significant effect on frequency in *HCN*$^{+/+}$ neurons (p = 0.99, Tukey's HSD, n = 5) but reduced frequency in *HCN1*$^{-/-}$ neurons (p = 0.0003).

Error bars in (C) and (E) indicate SEM.

HCN1 Channels Regulate Spiking Properties of IO Neurons by Controlling their Somatic Resting Membrane Potential

We next asked whether HCN1 channels control action potential initiation, as suggested by our pharmacological experiments (Figures 1G and 1H), and whether mechanisms similar to those controlling the inhibitory component of synaptic potentials are involved. We first investigated spontaneous action potential firing. Strikingly, and distinct from suggested pacemaker roles of I_h (Bal and McCormick, 1997), the frequency of spontaneous action potentials fired by IO neurons was increased by deletion of HCN1 ($p = 7 \times 10^{-5}$, unpaired t test) (Figures 4A and 4B). Unlike the inhibitory glutamatergic synaptic responses, which are not restored by depolarization of the somatic membrane potential (Figure 2), when the somatic membrane potential of neurons from *HCN1*$^{-/-}$ mice was adjusted to approximately −50 mV, the frequency of spontaneous action potentials became indistinguishable from *HCN1*$^{+/+}$ mice (p = 0.82, unpaired t test) (Figure 4C). Application of the blocker ZD7288 to neurons from *HCN1*$^{+/+}$ mice produced effects similar to deletion of HCN1 (Figure S4). Moreover, differences between *HCN1*$^{+/+}$ and *HCN1*$^{-/-}$ mice in their resting potential and spike frequency where abolished by ZD7288, indicating that these effects of HCN1 deletion also result directly from the absence of currents mediated by HCN1 channels and not from secondary adaptations (Figure S4). We also addressed possible effects of HCN1 on sinusoidal subthreshold oscillations. We observed sinusoidal subthreshold oscillations at resting potential in ~33% of *HCN1*$^{+/+}$ mice (n = 9/27), which is consistent with previous observations *in vivo* and *in vitro* (Khosrovani et al., 2007). We did not observe resting sinusoidal subthreshold oscillations from any *HCN1*$^{-/-}$ mice (n = 0/27), while the membrane potential at potentials equivalent to the

resting potential of $HCN1^{+/+}$ mice was dominated by ongoing asymmetric spikelet activity (Figure 4A), which likely reflects spontaneous spiking by IO neurons electrically coupled to the recorded cell.

The increase in firing rate following deletion of HCN1 is at first paradoxical given the associated profound hyperpolarization of the membrane potential. However, hyperpolarization of IO neurons can promote action potential firing through recruitment of T-type Ca^{2+} channels (Llinás and Yarom, 1981b). Consistent with this mechanism, we found that bath application of Ni^{+}, which blocks T-type channels (Lee et al., 1999), abolished spontaneous action potential firing in $HCN1^{-/-}$ mice (p = 0.0003, Tukey's HSD, n = 5), but not $HCN1^{+/+}$ mice (p = 0.99, Tukey's HSD) (Figures 4D and 4E). This differential effect of Ni^{+} does not result from upregulation of T-type channels following deletion of HCN1, as the amplitude and kinetics of T-type currents were similar in neurons from both groups of mice (p = 0.79, unpaired t test) (Figure S5). Thus, HCN1 channels in IO neurons suppress spontaneous firing by driving voltage-dependent inactivation of somatic T-type channels. This effect of HCN1 channels can explain why the threshold and probability of synaptically driven spike firing is not affected by block of I_h, even though there is a profound membrane potential hyperpolarization (Figures 1E and 1F).

Do the effects of HCN1 channels on the somatic resting membrane potential also explain changes in the latency and waveform of synaptically driven action potentials observed following pharmacological block of I_h? If they do, then these changes should also be reversed by depolarization of the somatic membrane potential. Consistent with this prediction, we found that during pharmacological block of I_h, the latency of synaptically driven action potentials was again increased (p = 0.018, Fisher's LSD, n = 5) and spikelets were abolished ($p = 2.54 \times 10^{-4}$), but following depolarization of the somatic membrane potential the spike latency (p = 0.28) and number of spikelets (p = 0.19) were indistinguishable from their values prior to application of ZD7288 (Figures 5A and 5B). If the sensitivity of spike content to ZD7288 reflects specific block of HCN1 channels then similar changes in the number of spikelets associated with spontaneous action potentials should be found in $HCN1^{-/-}$ mice. Indeed, there were 2.04 ± 0.38 spikelets during the ADP of spontaneous action potentials from $HCN1^{+/+}$ mice, whereas spikelets were completely absent during the ADP of spontaneous action potentials in $HCN1^{-/-}$ mice (0 ± 0 spikelets, n = 9) (Figure 5C). These and other measures of spike shape, which were also modified by deletion of HCN1 (Figures 5C and 5D) or pharmacological block of I_h (Figure S4), were rescued by restoration of the somatic membrane potential and were mimicked by somatic hyperpolarization of IO neurons from wild-type mice (Figures 5C, 5E, and S4D–S4F). Together, these data indicate that the influence of HCN1 channels on a neuron's somatic membrane potential controls its spontaneous and synaptically driven action potentials.

Deletion of HCN1 Increases Variability in Cerebellar Complex Spike Patterns Recorded during Quiet Wakefulness and Movement

Given the striking influence of HCN1 channels in the IO on synaptic integration and action potential initiation that we describe above, along with their previously described roles in pacemaking and resonance (Bal and McCormick, 1997; Matsumoto-Makidono et al., 2016), we asked whether HCN1 channels influence activity of IO neurons in behaving animals. As the IO is relatively difficult to access for direct electrophysiological recordings, we instead addressed this question using cell-attached recordings of complex spike activity from cerebellar Purkinje cells in awake, head-fixed mice (Figures 6A–6C). Because IO action potentials reliably trigger Purkinje cell complex spikes, and because complex spike duration is proportional to the number of spikelets in the IO spike (Mathy et al., 2009), these properties serve as a readout of activity in the IO (Eccles et al., 1964). We focused on Purkinje cells in the vermis of lobule V of the cerebellum, as this region integrates sensory input with motor commands and is involved in adaptive motor coordination (Apps and Hawkes, 2009).

We compared complex spike activity between $HCN1^{+/+}$ and $HCN1^{-/-}$ mice during quiet wakefulness and periods of movement (Figures 6D–6E). We find that deletion of HCN1 causes a striking change in the coefficient of variation (CV) of the interval between complex spikes in both behavioral states (Figures 6G, 6K, S6A, and S6B; Table S1). This was manifest as an increase in the mean CV across the population and in the variability of the CV between cells (Table S1). These changes in firing pattern were accompanied by a smaller reduction in complex spike frequency during quiet wakefulness, but not during movement (Figures 6F and 6J; Table S1). When we evaluated the number of spikelets and complex spike duration, we did not find significant differences during quiet wakefulness (Figures 6H and 6I), but both were reduced during movement (Figures 6L and 6M). We also investigated the effect of movement within cells. We did not find significant differences in any of the measured complex spike properties between quiet wakefulness and movement in either $HCN1^{+/+}$ or $HCN1^{-/-}$ mice (Figures S6C–S6N; Table S2; see also Jelitai et al., 2016). The frequency of simple spikes was not affected by a global deletion of HCN1 (Table S1). Thus, HCN1 channels do not appear to impact the frequency of simple spikes fired by cerebellar Purkinje cells and have relatively little effect on the frequency of complex spikes originating from the IO. Instead, these data are consistent with HCN1 channels influencing the timing of action potential firing in the IO and the number of spikelets following an action potential during movement.

DISCUSSION

Our results establish local and network-wide roles for HCN1 channels in control of synaptic integration in the IO and provide evidence that in behaving animals HCN1 channels in the IO influence spike timing. We find that HCN1 channels are required for I_h in IO neurons and contribute substantially to their resting and active membrane properties. HCN1 channels acting in part via gap junctions from adjoining IO neurons enable the inhibitory component of the PSP, whereas HCN1 channels acting at the soma control the timing and content of action potentials fired by IO neurons. The combination of local and long-distance actions of HCN1 channels, which contrast with their roles in other

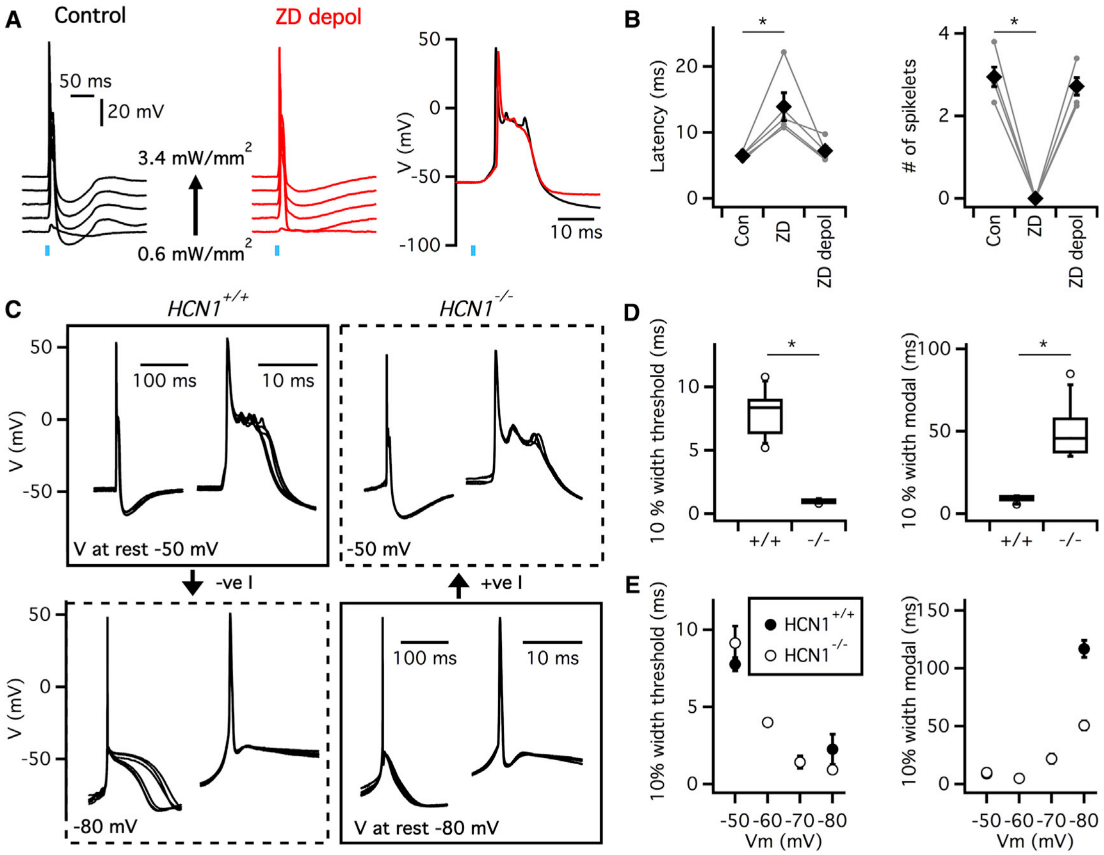

Figure 5. Waveforms of Spontaneous Action Potentials Are Modified by Deletion of HCN1

(A) Examples of synaptic responses of IO neurons from Thy1-Chr2 mice to varying intensity optical stimulation in control conditions (left) and during perfusion of ZD7288 while also injecting positive current to restore the membrane potential to its control value (center). Threshold spikes shown on an expanded timescale indicate that spike latency and number of spikelets are similar (right).

(B) Latency (left) and number of spikelets (right) for synaptically driven action potentials activated in control conditions (Con), during perfusion of ZD7288 (ZD) and during perfusion of ZD7288 with the membrane potential restored to its control value (ZD depol). Perfusion of ZD7288 modifies the latency ($F_{2,8} = 11.5$ $p = 0.004$ for effect of condition, one-way repeated-measures ANOVA; control versus ZD7288, $p = 0.015$, Fisher's LSD, $n = 5$) and number of spikelets ($F_{2,8} = 149.5$ $p = 4.6 \times 10^{-7}$ and $p = 8.4 \times 10^{-8}$, respectively), but after restoration of the somatic membrane potential, both are indistinguishable from their control values (control versus ZD depol, $p = 0.68$ and $p = 0.39$ respectively, Fisher's LSD, $n = 5$).

(C) Voltage waveforms of action potentials recorded from IO neurons in the absence of injected current (solid line boxes) and when the membrane potential is adjusted by injection of negative (*HCN1*$^{+/+}$) or positive current (*HCN1*$^{-/-}$) (broken line boxes). In each box waveforms to the right show the action potentials on an expanded timescale.

(D) Boxplots of width of the action potential waveform measured relative to the estimated threshold for initiation of the action potential ($p = 4.3 \times 10^{-10}$, t test, $n = 9$) and width of the action potential complex measured relative to the modal membrane potential ($p = 6.0 \times 10^{-7}$).

(E) Width of the spike ($F_{1,24} = 37.9$ $p = 2.3 \times 10^{-6}$ for interaction between genotype and membrane potential, ANOVA) (left) and the spike complex ($F_{1,24} = 31.9$ $p = 8.2 \times 10^{-6}$ effect of genotype, two-way ANOVA) (right), plotted as a function of membrane potential.

Error bars in (B) and (E) indicate SEM.

neuron types, supports the idea that the IO carries out distinct network-level computations.

Distal HCN1 Channel Signaling Enables Inhibitory Components of Glutamatergic Synaptic Responses

How do HCN1 channels enable the hyperpolarizing component of the response to glutamatergic synaptic input? How can this requirement for HCN1 channels be reconciled with previous findings that the hyperpolarizing component is mediated by calcium-activated potassium channels (Garden et al., 2017)? By demonstrating that inhibitory components of bidirectional GluA-mediated synaptic responses are abolished by pharmacological and genetic manipulations of HCN1 channels, we provide direct evidence for a role of HCN1 while also ruling out off-target effects of I_h blockers (Felix et al., 2003) or adaptation following genetic manipulations (Chen et al., 2010). The direct contribution of I_h to the resting membrane conductance is unlikely to explain the requirement of I_h for inhibitory responses, as in this case, blocking I_h would substantially increase the amplitude of the depolarizing response (cf. Stuart and Spruston, 1998), whereas the maximal amplitude of this component is either maintained or slightly reduced after deletion of HCN1 or block

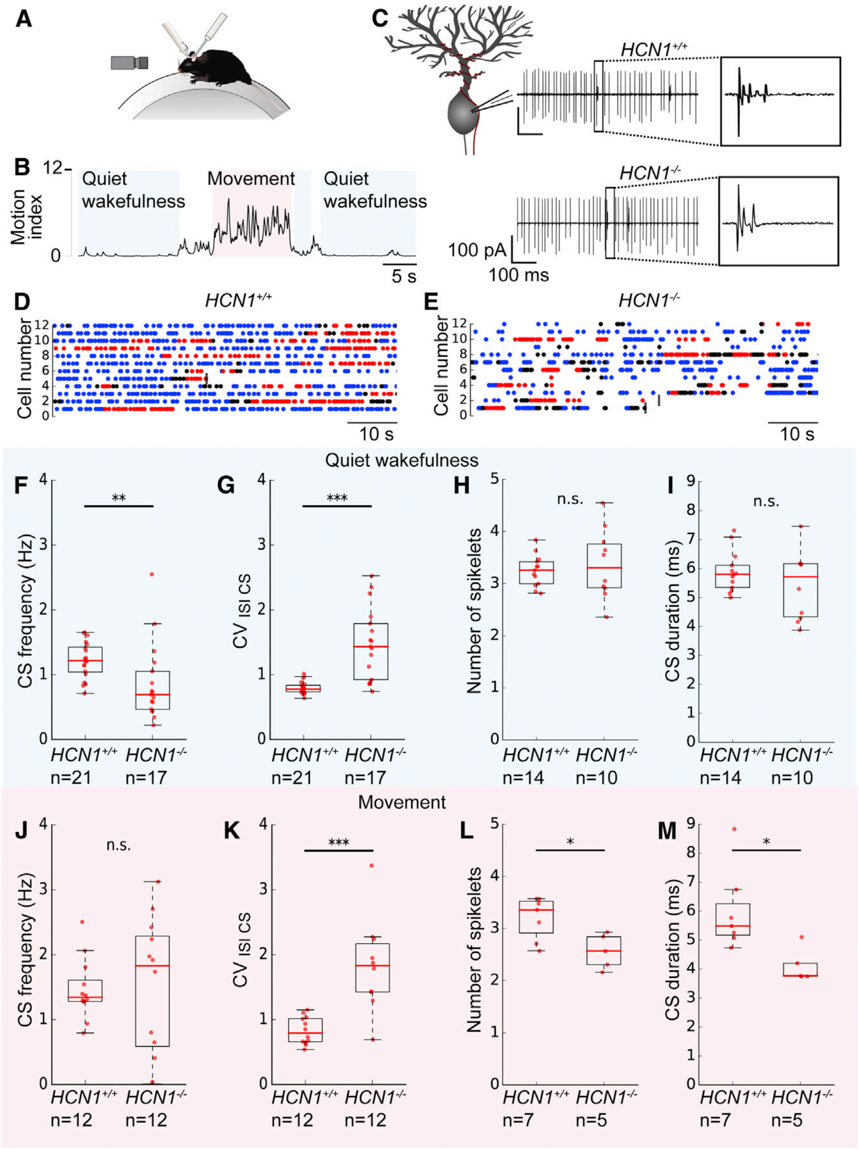

Figure 6. Deletion of HCN1 Increases Variability in the Timing of Complex Spikes Recorded from Cerebellar Purkinje Cells

(A) Schematic of the *in vivo* recording setup. The head-fixed mouse is able to run on a cylindrical treadmill. A recording electrode is lowered through the cerebellar cortex to reach the Purkinje cell layer.

(B) A motion index is calculated based on video analysis to determine clear periods of quiet wakefulness and movement.

(C) Examples of cell-attached patch clamp recordings from $HCN1^{+/+}$ and $HCN1^{-/-}$ Purkinje cells showing simple spikes and complex spikes. The enlarged area shows complex spike waveforms.

(D and E) Examples of the distribution of complex spikes from individual Purkinje cells over time in $HCN1^{+/+}$ mice (D) and in $HCN1^{-/-}$ mice (E). Each line represents data from one Purkinje cell. Blue dots indicate a complex spike during a period of quiet wakefulness, and red dots indicate a complex spike during a period of movement. Black dots indicate a complex spike in a period that cannot be clearly defined as either quite wakefulness or movement. A maximum of 70 s is shown, or less if the recording was of a shorter duration, in which case a vertical dark gray line indicates the end of the recording.

(F–I) During quiet wakefulness, complex spikes in $HCN1^{-/-}$ mice have reduced frequency (p = 0.002, Mann-Whitney U test) (F) and increased CV (p = 0.000) (G) compared to $HCN1^{+/+}$ mice. The number of spikelets (p = 0.828) (H) and complex spike duration (p = 0.734) (I) do not differ significantly (Table S1).

(J–M) During movement, $HCN1^{-/-}$ mice show an increase in the CV of complex spike firing compared to $HCN1^{+/+}$ mice (p = 0.001, Mann-Whitney *U* test) (K) but no change in complex spike frequency (p = 0.840) (J). The number of spikelets per complex spike (p = 0.034) (L) and complex spike duration (p = 0.014) (M) are both also reduced (Table S1).

of I_h (Figures 2 and 3). Instead, our observations can be explained in the framework of a two-stage model of synaptic integration by IO neurons (Kistler and De Zeeuw, 2005). According to this model, the hyperpolarizing component of the glutamatergic PSP is mediated by Ca^{2+}-activated potassium channels, whose opening is driven by local Ca^{2+} spikes triggered by the depolarizing component of the synaptic response (Kistler and De Zeeuw, 2005). This model is supported by pharmacological and electrophysiological analysis of glutamatergic inputs to neurons in the IO (Garden et al., 2017). In this scenario, resting activation of HCN1 channels located on dendrites (Matsumoto-Makidono et al., 2016) will maintain depolarization of spines within synaptic glomeruli, which in turn enables synaptic input to trigger dendritic Ca^{2+} spikes. Thus, in the absence of HCN1, the dendrite is hyperpolarized and glutamatergic input can no longer trigger the Ca^{2+} spikes. This may explain the reduction in the peak of the depolarizing components of the glutamatergic PSP following deletion of HCN1 (Figure 2) and pharmacological block of I_h (Figure 3), while the absence of a resulting Ca^{2+} influx and activation of Ca^{2+}-activated potassium channels can account for the abolished hyperpolarizing response to glutamatergic inputs.

Our results suggest that the control of hyperpolarizing components of the PSP by HCN1 channels involves network-wide actions mediated by gap junctions. Whereas functions of HCN1 channels are usually restricted to the neuron in which they are expressed (Magee, 2000), differential actions of intracellular and extracellular block of I_h, along with effects of gap junction block, indicate that the inhibitory components of PSPs involve actions of HCN1 channels in adjoining electrically connected neurons (Figure 3). The relative insensitivity of synaptic responses to changes in the somatic membrane potential of the recorded cell (Figure 4) is also consistent with synaptic potentials

originating at locations that are electrically distant from the soma. Given that glomeruli at which IO neurons receive excitatory inputs are connected by gap junctions (Kistler and De Zeeuw, 2005), HCN1 channels in all cells contributing to a glomerulus may permit bidirectional responses by maintaining the membrane potential of spines in a depolarized state so that subsequent excitatory input can trigger local spikes. Therefore, inhibitory components of the synaptic response should still be present when a $HCN1^{+/+}$ neuron is hyperpolarized or when HCN channels are blocked by intracellular ZD7288. This is consistent with our experimental observations (Figure 3). In contrast, if depolarization from HCN1 channels is absent in all neurons and all of the spines within the glomeruli are hyperpolarized, then synaptic activation of dendritic Ca^{2+} spikes and subsequent activation of small conductance (SK) or large conductance (BK) calcium-activated potassium channels becomes unlikely, and no inhibitory component will be recorded at the soma. This is consistent with our recordings both from $HCN1^{-/-}$ mice and from $HCN1^{+/+}$ mice during extracellular application of ZD7288 (Figure 3). These interactions between gap junctions, intrinsic properties, and synaptic input appear distinct from synaptic integration in largely passive dendrites of cerebellar Golgi cells, which are also connected by gap junctions (Vervaeke et al., 2012).

Local Actions of HCN1 Channels Controls Action Potential Initiation and Content

In addition to controlling subthreshold integration, we found that HCN1 channels reduce the latency for synaptically driven action potential firing and support generation of spikelets during the action potential afterdepolarization (Figures 1 and 5). In several neuronal cell types that generate action potentials spontaneously, I_h is thought to act as an excitatory pacemaker current (Robinson and Siegelbaum, 2003). In contrast, in IO neurons, HCN1 channels reduce the frequency of spontaneous action potential firing but are required for the prolonged action potential ADP and its superimposed spikelets (Figures 4 and 5). These observations are initially paradoxical, as inward current flowing through HCN1 channels should drive pacemaking at resting potentials (Bal and McCormick, 1997), while HCN1 channels close at positive membrane potentials reached during the action potential ADP (Figure 1C). However, they are consistent with previous reports that T-type calcium channels drive spontaneous activity of hyperpolarized IO neurons (Llinás and Yarom, 1986) and observations of the voltage-dependence of the ADP (Llinás and Yarom, 1981a). Thus, the depolarizing influence of HCN1 channels causes inactivation of T-type Ca^{2+} channels, which prevents T-type Ca^{2+} channels from driving spontaneous firing, and enables the spike ADP. This interpretation is consistent with the reversibility of these phenotypes by somatic depolarization (Figures 4 and 5) and a lack of evidence for adaptation by other membrane conductances following deletion of HCN1 (Figures S5). In other neuron types, I_h also controls excitability via its actions on membrane potential, causing modified gating of voltage-gated ion channels (George et al., 2009). Just as for the influence of HCN1 channels on the excitability of IO neurons, these functions reflect influence of I_h on signaling within the recorded cell.

Implications for Computation by IO Networks during Motor Behavior

How do HCN1 channels influence the firing of IO neurons during behavior? Using complex spike firing by Purkinje cells as a readout of firing by neurons in the IO, we find that *in vivo* HCN1 channels primarily affect the pattern of climbing fiber activity. This is apparent as a striking increase in the variability of the interval between complex spikes. This increase in complex spike CV occurs both during quiet wakefulness and during movement (Figures 6D, 6E, 6G, and 6K). A dominant role for HCN1 in controlling synaptic integration within the IO may be consistent with these observations. Thus, HCN1 determines the timing of action potentials triggered by the depolarizing component of glutamatergic PSPs (Figure 1), and because it is required for the hyperpolarizing component of the PSP (Figures 1 and 2), it should also determine the spatial and temporal integration of bidirectional glutamatergic responses (cf. Garden et al., 2017). The changes to spike timing *in vivo* appear unlikely to be accounted for by a direct influence of HCN1 channels on spontaneous spiking of IO neurons, as we find that *in vitro*, when background synaptic activity is absent, HCN1 channels suppress spontaneous firing and increase the variability of spike intervals (Figure 4). This is the opposite of our finding in behaving animals. This difference may be because *in vivo* glutamatergic synaptic input drives spontaneous activity of IO neurons (Lang, 2001). Resonant or oscillatory roles of HCN1 channels also appear unlikely to explain the increase in variability of complex spike timing, as both would act on timescales in the 5- to 10-Hz range (interspike intervals of 100–200 ms), whereas the interspike intervals that contribute to increased variability are much longer (Figure S6). The changes in firing pattern are also unlikely to result from an absence of HCN1 from cerebellar Purkinje cells, as deletion of HCN1 channels does not affect the initiation or properties of Purkinje cell complex spikes (Rinaldi et al., 2013). Together, these observations indicate that models for computation by the IO must account for multiple complementary roles of HCN1 channels and point toward the importance of the influence of excitability on synaptic integration within the IO. These changes would be expected to influence motor coordination through altered signaling and plasticity in the cerebellar cortex; for example, through climbing-fiber-driven modification of parallel fiber input to Purkinje cells (Mathy et al., 2009), as well as modifications to climbing fiber synapses and intrinsic plasticity in Purkinje cells (Grasselli et al., 2016; Hansel and Linden, 2000; Ohtsuki et al., 2012).

In conclusion, our results indicate that integrative mechanisms in the IO are engaged during movement. HCN1 channels have both local and long-range actions in the IO, while their disruption modifies patterns of IO firing during behavior. This diversity of cellular functions for a single ion channel is consistent with the evolution of combinatorial patterns of ion channel expression that enable particular neuron types to perform specific computations (Marder and Goaillard, 2006).

EXPERIMENTAL PROCEDURES

Further details and an outline of methods and resources used in this work can be found in Supplemental Experimental Procedures.

Animals

Experimental studies conformed to the policies of the UK Animals (Scientific Procedures) Act 1986 and European Directive 2010/62/EU on the protection of animals used for experimental purposes. Experiments were carried out under a project license granted by the UK Home Office and according to the guidelines laid down by the University of Edinburgh's Animal Welfare Committee.

C57BL/6 mice (all males), mice expressing ChR2 under the control of the Thy1 promoter (Thy1-ChR2-YFP line 18, stock number 007612, The Jackson Laboratory, Bar Harbor, ME) (Arenkiel et al., 2007), and mice with a global deletion of HCN1 ($HCN1^{-/-}$; Nolan et al., 2003) and their wild-type littermates ($HCN^{+/+}$) (both males and females) were housed on a 12-hr light/dark cycle (light on 7:00–19.00 hr) in standard breeding cages. Food and water were available *ad libitum*. For brain slice experiments, the median age of mice used was 46 days (range 28–116 days). For *in vivo* experiments, the median age of mice used was 56.5 days (range, 48–76 days). During all experiments, the experimenter was blind to the group the mice were in.

Data Analysis and Statistical Methods

In vitro electrophysiological data were analyzed in IGOR pro (Wavemetrics) using Neuromatic (http://www.neuromatic.thinkrandom.com/) and custom-written routines or Axograph. *In vivo* electrophysiological data were analyzed using custom-written programs in Python (https://www.python.org). Simple spikes, complex spikes, and their associated spikelets were automatically detected and then visually verified. The reported number of spikelets per complex spike excludes the initial sodium spike component. Complex spike duration was defined as the time between the peak of the first sodium spike of the complex spike to the peak of the last spikelet of the same complex spike. Further statistical analysis was carried out using Python, IGOR pro, Excel (Microsoft), IBM SPSS Statistics version 17.0 (NY, USA), or R (www.R-project.org). Mean values are reported as ± SEM. Statistical significance was tested with linear regression, Student's t test, one-way ANOVA, and post hoc Fisher's LSD or Tukey's HSD where appropriate, two-way repeated-measures ANOVA, the Kolmogorov-Smirnov test, or the Mann-Whitney *U* test.

SUPPLEMENTAL INFORMATION

Supplemental Information includes Supplemental Experimental Procedures, six figures, and two tables and can be found with this article online at https://doi.org/10.1016/j.celrep.2018.01.069.

ACKNOWLEDGMENTS

This work was supported by the Medical Research Council (G0501216), the Wellcome Trust (093295/Z/10/Z and 086602/Z/08/Z), and the BBSRC (Bb/H020284/1). We thank Paolo Puggioni for help with motion analysis and the IMPACT facility at the University of Edinburgh for imaging resources.

AUTHOR CONTRIBUTIONS

Conceptualization, D.L.F.G., I.D., M.F.N., and M.O.; Methodology, D.L.F.G., M.O., M.J., A.R., I.D., and M.F.N.; Formal Analysis, D.L.F.G., M.N., and M.O.; Investigation, D.L.F.G. and M.O.; Writing – Original Draft: D.L.F.G., M.F.N., and M.O.; Writing – Review & Editing, D.L.F.G., M.O., M.J., A.R., I.D., and M.F.N.; Visualization, D.L.F.G. and M.O.; Supervision, I.D. and M.F.N.; Project Administration, M.N.; Funding Acquisition, I.D. and M.F.N.

DECLARATION OF INTERESTS

The authors declare no competing interests.

Received: December 20, 2016
Revised: January 3, 2018
Accepted: January 22, 2018
Published: February 13, 2018

REFERENCES

Albus, J.S. (1970). A theory of cerebellar function. Math. Biosci. *10*, 25–61.

Apps, R., and Hawkes, R. (2009). Cerebellar cortical organization: a one-map hypothesis. Nat. Rev. Neurosci. *10*, 670–681.

Arenkiel, B.R., Peca, J., Davison, I.G., Feliciano, C., Deisseroth, K., Augustine, G.J., Ehlers, M.D., and Feng, G. (2007). In vivo light-induced activation of neural circuitry in transgenic mice expressing channelrhodopsin-2. Neuron *54*, 205–218.

Bal, T., and McCormick, D.A. (1997). Synchronized oscillations in the inferior olive are controlled by the hyperpolarization-activated cation current I(h). J. Neurophysiol. *77*, 3145–3156.

Benardo, L.S., and Foster, R.E. (1986). Oscillatory behavior in inferior olive neurons: mechanism, modulation, cell aggregates. Brain Res. Bull. *17*, 773–784.

Chen, X., Shu, S., Schwartz, L.C., Sun, C., Kapur, J., and Bayliss, D.A. (2010). Homeostatic regulation of synaptic excitability: tonic GABA(A) receptor currents replace I(h) in cortical pyramidal neurons of HCN1 knock-out mice. J. Neurosci. *30*, 2611–2622.

Dayan, P., and Abbott, L.F. (2001). Theoretical Neuroscience: Computational and Mathematical Modeling of Neural Systems (MIT Press).

de Zeeuw, C.I., Ruigrok, T.J., Holstege, J.C., Jansen, H.G., and Voogd, J. (1990). Intracellular labeling of neurons in the medial accessory olive of the cat: II. Ultrastructure of dendritic spines and their GABAergic innervation. J. Comp. Neurol. *300*, 478–494.

De Zeeuw, C.I., Simpson, J.I., Hoogenraad, C.C., Galjart, N., Koekkoek, S.K., and Ruigrok, T.J. (1998). Microcircuitry and function of the inferior olive. Trends Neurosci. *21*, 391–400.

De Zeeuw, C.I., Hoebeek, F.E., Bosman, L.W., Schonewille, M., Witter, L., and Koekkoek, S.K. (2011). Spatiotemporal firing patterns in the cerebellum. Nat. Rev. Neurosci. *12*, 327–344.

Eccles, J., Llinas, R., and Sasaki, K. (1964). Excitation of cerebellar Purkinje cells by the climbing fibres. Nature *203*, 245–246.

Felix, R., Sandoval, A., Sánchez, D., Gómora, J.C., De la Vega-Beltrán, J.L., Treviño, C.L., and Darszon, A. (2003). ZD7288 inhibits low-threshold Ca(2+) channel activity and regulates sperm function. Biochem. Biophys. Res. Commun. *311*, 187–192.

Garden, D.L., Rinaldi, A., and Nolan, M.F. (2017). Active integration of glutamatergic input to the inferior olive generates bidirectional postsynaptic potentials. J. Physiol. *595*, 1239–1251.

George, M.S., Abbott, L.F., and Siegelbaum, S.A. (2009). HCN hyperpolarization-activated cation channels inhibit EPSPs by interactions with M-type K(+) channels. Nat. Neurosci. *12*, 577–584.

Grasselli, G., He, Q., Wan, V., Adelman, J.P., Ohtsuki, G., and Hansel, C. (2016). Activity-Dependent Plasticity of Spike Pauses in Cerebellar Purkinje Cells. Cell Rep. *14*, 2546–2553.

Hansel, C., and Linden, D.J. (2000). Long-term depression of the cerebellar climbing fiber–Purkinje neuron synapse. Neuron *26*, 473–482.

Jelitai, M., Puggioni, P., Ishikawa, T., Rinaldi, A., and Duguid, I. (2016). Dendritic excitation-inhibition balance shapes cerebellar output during motor behaviour. Nat. Commun. *7*, 13722.

Khosrovani, S., Van Der Giessen, R.S., De Zeeuw, C.I., and De Jeu, M.T. (2007). In vivo mouse inferior olive neurons exhibit heterogeneous subthreshold oscillations and spiking patterns. Proc. Natl. Acad. Sci. USA *104*, 15911–15916.

Kistler, W.M., and De Zeeuw, C.I. (2005). Gap junctions synchronize synaptic input rather than spike output of olivary neurons. Prog. Brain Res. *148*, 189–197.

Lang, E.J. (2001). Organization of olivocerebellar activity in the absence of excitatory glutamatergic input. J. Neurosci. *21*, 1663–1675.

Lee, J.H., Gomora, J.C., Cribbs, L.L., and Perez-Reyes, E. (1999). Nickel block of three cloned T-type calcium channels: low concentrations selectively block alpha1H. Biophys. J. *77*, 3034–3042.

Leznik, E., and Llinás, R. (2005). Role of gap junctions in synchronized neuronal oscillations in the inferior olive. J. Neurophysiol. *94*, 2447–2456.

Llinás, R., and Yarom, Y. (1981a). Electrophysiology of mammalian inferior olivary neurones in vitro. Different types of voltage-dependent ionic conductances. J. Physiol. *315*, 549–567.

Llinás, R., and Yarom, Y. (1981b). Properties and distribution of ionic conductances generating electroresponsiveness of mammalian inferior olivary neurones in vitro. J. Physiol. *315*, 569–584.

Llinás, R., and Yarom, Y. (1986). Oscillatory properties of guinea-pig inferior olivary neurones and their pharmacological modulation: an in vitro study. J. Physiol. *376*, 163–182.

Llinas, R., Baker, R., and Sotelo, C. (1974). Electrotonic coupling between neurons in cat inferior olive. J. Neurophysiol. *37*, 560–571.

Long, M.A., Deans, M.R., Paul, D.L., and Connors, B.W. (2002). Rhythmicity without synchrony in the electrically uncoupled inferior olive. J. Neurosci. *22*, 10898–10905.

Magee, J.C. (2000). Dendritic integration of excitatory synaptic input. Nat. Rev. Neurosci. *1*, 181–190.

Marder, E., and Goaillard, J.M. (2006). Variability, compensation and homeostasis in neuron and network function. Nat. Rev. Neurosci. *7*, 563–574.

Marr, D. (1969). A theory of cerebellar cortex. J. Physiol. *202*, 437–470.

Mathy, A., Ho, S.S., Davie, J.T., Duguid, I.C., Clark, B.A., and Häusser, M. (2009). Encoding of oscillations by axonal bursts in inferior olive neurons. Neuron *62*, 388–399.

Matsumoto-Makidono, Y., Nakayama, H., Yamasaki, M., Miyazaki, T., Kobayashi, K., Watanabe, M., Kano, M., Sakimura, K., and Hashimoto, K. (2016). Ionic basis for membrane potential resonance in neurons of the inferior olive. Cell Rep. *16*, 994–1004.

Nolan, M.F., Malleret, G., Lee, K.H., Gibbs, E., Dudman, J.T., Santoro, B., Yin, D., Thompson, R.F., Siegelbaum, S.A., Kandel, E.R., and Morozov, A. (2003). The hyperpolarization-activated HCN1 channel is important for motor learning and neuronal integration by cerebellar Purkinje cells. Cell *115*, 551–564.

Notomi, T., and Shigemoto, R. (2004). Immunohistochemical localization of Ih channel subunits, HCN1-4, in the rat brain. J. Comp. Neurol. *471*, 241–276.

Ohtsuki, G., Piochon, C., Adelman, J.P., and Hansel, C. (2012). SK2 channel modulation contributes to compartment-specific dendritic plasticity in cerebellar Purkinje cells. Neuron *75*, 108–120.

Placantonakis, D.G., Bukovsky, A.A., Aicher, S.A., Kiem, H.P., and Welsh, J.P. (2006). Continuous electrical oscillations emerge from a coupled network: a study of the inferior olive using lentiviral knockdown of connexin36. J. Neurosci. *26*, 5008–5016.

Rinaldi, A., Defterali, C., Mialot, A., Garden, D.L., Beraneck, M., and Nolan, M.F. (2013). HCN1 channels in cerebellar Purkinje cells promote late stages of learning and constrain synaptic inhibition. J. Physiol. *591*, 5691–5709.

Robinson, R.B., and Siegelbaum, S.A. (2003). Hyperpolarization-activated cation currents: from molecules to physiological function. Annu. Rev. Physiol. *65*, 453–480.

Santoro, B., Chen, S., Luthi, A., Pavlidis, P., Shumyatsky, G.P., Tibbs, G.R., and Siegelbaum, S.A. (2000). Molecular and functional heterogeneity of hyperpolarization-activated pacemaker channels in the mouse CNS. J. Neurosci. *20*, 5264–5275.

Stuart, G., and Spruston, N. (1998). Determinants of voltage attenuation in neocortical pyramidal neuron dendrites. J. Neurosci. *18*, 3501–3510.

Turecek, J., Yuen, G.S., Han, V.Z., Zeng, X.H., Bayer, K.U., and Welsh, J.P. (2014). NMDA receptor activation strengthens weak electrical coupling in mammalian brain. Neuron *81*, 1375–1388.

Vervaeke, K., Lorincz, A., Nusser, Z., and Silver, R.A. (2012). Gap junctions compensate for sublinear dendritic integration in an inhibitory network. Science *335*, 1624–1628.

Welsh, J.P., and Llinás, R. (1997). Some organizing principles for the control of movement based on olivocerebellar physiology. Prog. Brain Res. *114*, 449–461.

Cell Reports, Volume *22*

Supplemental Information

Inferior Olive HCN1 Channels Coordinate Synaptic Integration and Complex Spike Timing

Derek L.F. Garden, Marlies Oostland, Marta Jelitai, Arianna Rinaldi, Ian Duguid, and Matthew F. Nolan

SUPPLEMENTAL EXPERIMENTAL PROCEDURES

CONTACT FOR REAGENT AND RESOURCE SHARING

Matthew Nolan (mattnolan@ed.ac.uk) is the Lead Contact for reagent and resource sharing. All published reagents will be shared on an unrestricted basis; reagent requests should be directed to the lead author.

EXPERIMENTAL MODEL AND SUBJECT DETAILS

Experimental studies conformed to the policies of the UK Animals (Scientific Procedures) Act 1986 and European Directive 2010/62/EU on the protection of animals used for experimental purposes. Experiments were carried out under a project licence granted by the UK Home Office and according to the guidelines laid down by the University of Edinburgh's Animal Welfare Committee.

C57BL/6 mice, all males, mice expressing ChR2 under the control of the Thy1 promoter (Thy1-ChR2-YFP line 18, stock number 007612 from The Jackson Laboratory, Barr Harbor, ME)(Arenkiel et al., 2007), and mice with a global deletion of HCN1 ($HCN1^{-/-}$, Nolan et al. 2003) and their wildtype littermates ($HCN^{+/+}$), both males and females, were housed on a 12h light/dark cycle (light on 7:00 – 19.00h) in standard breeding cages. Food and water were available ad libitum. During all experiments the experimenter was blind to the group the mice were in.

METHOD DETAILS

Virus injections. C57BL/6 mice, all males, aged 5–6 weeks, were anesthetized with isoflurane and kept on a feedback-controlled heating pad (Homeothermic Blanket System 50300, Stoelting). Post-operative pain was prevented by administering 0.05 mg/kg buprenorphine hydrochloride (Vetergesic) during the procedure, and by giving the animals access to Vetergesic in jelly form during recovery. Injections targeting the motor cortex were carried out as described previously (Garden et al., 2017). The mice were mounted into a stereotaxic frame and a cut was made to expose the skull. For

injections into the neocortex, holes were drilled in the skull bilaterally above areas containing primarily M1 and M2 (1.2-1.4 ML, 1.0-1.5 AP from bregma) and the underlying dura was carefully removed. A pipette was then inserted at a depth of 1 mm from the pial surface and 500 nl of adeno-associated virus (AAV) (pACAGW-ChR2-Venus, Vector biolabs) was injected over 5 minutes. Pipettes were left for 5 minutes post-injection before removal.

In vitro electrophysiology. Slice preparation and patch-clamp recording from neurons in the IO was as described previously (Garden et al., 2017). The median age of mice used was 46 days (range 28 - 116 days). We did not find any change in measured parameters with age of the mice. Mice were killed by decapitation following isoflurane anesthesia and their brains rapidly removed, and placed in cooled (4-6 °C) oxygenated modified artificial cerebrospinal fluid (ACSF) composed of the following (in mM): NaCl (86), KCl (2.5), $CaCl_2$ (0.5), $MgCl_2$ (7), NaH_2PO_4 (1.2), $NaHCO_3$ (25), glucose (25), and sucrose (75), continuously bubbled with 95 % O_2 and 5 % CO_2 (pH = 7.4). The brain was placed ventral-side up and a coronal cut made through the widest part of the brainstem and cerebellum. The cut surface was glued to the stage of a sectioning system (Leica VT1200), with the caudal part of the brain facing upwards. Coronal sections of thickness 200 µm were cut submerged under cold modified ACSF. After slicing, brain slices were immediately immersed in regular ACSF, consisting of the following (in mM): NaCl (124), KCl (2.5), $CaCl_2$ (2), $MgCl_2$(1), NaH_2PO_4 (1.2), $NaHCO_3$ (25), and glucose (20), continuously bubbled with 95 % O2 and 5 % CO2 (pH=7.4). Slices were kept at a temperature of 33 – 35°C for 10 – 20 mins and then passively cooled to room temperature (20 – 24°C). Neurons in the IO were visually identified under infrared illumination with DIC optics. Whole-cell recordings were obtained at 35°C–37°C from the soma of IO neurons using electrodes with resistance 2–5 MΩ when filled with intracellular solution containing the following (in mM): K gluconate (130), KCl (10), EGTA (0.5), HEPES (10), MgCl (2), EGTA (0.1), NA_2ATP (2), NA_2GTP (0.3), and phosphocreatine (10)(pH adjusted to 7.3 with KOH). Recordings were made using a Multiclamp 700B amplifier (Molecular Devices, Sunnydale) and Axograph X software (Axograph Scientific, Sydney). Series resistances were < 15 MΩ for voltage-clamp experiments and < 40 MΩ for current-clamp experiments. Series resistance in voltage-clamp recordings was compensated by 70%–80%. For current-clamp recordings

appropriate bridge and electrode capacitance compensations were applied. Membrane current and voltage were filtered at 1–2 KHz and 4–20 KHz and sampled at 5–10 KHz and 10–50 KHz for voltage- and current-clamp experiments, respectively. Input resistance was calculated from the steady-state voltage response to injected 80 pA current steps. The sag was calculated as the steady-state voltage response to an injected negative current of 160 pA divided by the peak of this response. A lower value represents a larger membrane potential sag.

For optogenetic activation of ChR2 expressing axons an LED was attached to the epifluorescence port of the microscope used for identification of recorded neurons (see (Garden et al., 2017)). Activation of the LED was controlled by an analogue voltage output from a data acquisition board.

Measurement of I_h was carried out in ACSF of the following composition (in mM): NaCl (115), NaH_2PO_4 (1.2), KCl (5), $NaHCO_3$ (25), glucose (20), $CaCl_2$ (2), $MgCl_2$ (1), $BaCl_2$ (1), $CdCl_2$ (0.1), 4-AP (1), TEA (5), NBQX (0.005), picrotoxin (0.05), and TTX (0.0005). Experiments to examine the effects of pharmacological block of ion channels on the light-evoked responses were performed with blockers of ionotropic glutamate and GABA receptors added to the ACSF. All chemicals were purchased from Sigma (St. Louis, MO) with the exception of NBQX, D-AP5, picrotoxin and ZD7288 from Abcam biochemicals (Cambridge, UK). Drugs were made fresh daily from frozen stocks concentrated 1000-fold.

In vivo electrophysiology in awake mice. Mice with global deletion of HCN1 (*HCN1*$^{-/-}$) and their wild-type littermates (*HCN1*$^{+/+}$), both males and females, were obtained as previously described (Nolan et al. 2003). For all experiments, the mice were on a mixed average 50:50% 129SVEV:C57BL/6 background. Genotype was determined from ear notch biopsies by real-time PCR (Transnetyx, Cordova, TN, USA) and confirmed after each experiment using DNA from tail biopsies. Mice were housed in standard breeding cages with access to a running wheel for at least a week before the start of the experiment. Mice were kept on a reversed 12h light/dark cycle (light on 19:00 – 7:00h) for 2-4 weeks before the start of the experiment.

Surgical procedures and *in vivo* awake recordings were performed as previously described (Jelitai et al., 2016). All surgical procedures were performed under 1.5% isoflurane anaesthesia and with mice kept on a feedback-controlled heating pad (Homeothermic Blanket System 50300, Stoelting). A small lightweight headplate (0.75 g) was implanted using cyanoacrylate adhesive and dental acrylic (Jet Denture Repair, Lang Dental Manufacturing Co.) and sealed with a Kwik-Cast sealant (World Precision Instruments). Post-operative pain was prevented by administering 0.05 mg/kg buprenorphine hydrochloride (Vetergesic) during the procedure. After at least 24 hours of recovery, the Kwik-Cast sealant was removed and a craniectomy (~300 x 300 μm, using a Volvere Max GX35 hand drill, NSK Dental) was performed above lobule V of the cerebellum (2.5 mm posterior to lambda and 0.75 mm lateral to midline) and the dura removed. The craniectomy was sealed with agar (1.5%) and Kwik-Cast sealant and mice were returned to the home cage for ~1 hr to recover from anaesthesia before recording commenced. Post-operative pain was prevented by administering a non-steroidal, anti-inflammatory agent carprofen (4 mg/kg, Rimadyl) during the procedure.

In vivo cell-attached recordings were made from Purkinje cells in lobule V of the cerebellar vermis of awake mice head-fixed on a spherical treadmill. Mice were habituated to the head-restraint and experimental setup for 30-60 minutes before each recording session. Head-restrained mice were free to run, walk or sit on the cylindrical treadmill. The Kwik-Cast sealant was removed at the start of the recording. *In vivo* external solution consisted of the following (in mM): NaCl (150), KCl (2.5), HEPES (10), CaCl2 (1.5), MgCl2 (1), with pH 7.3. Glass pipettes (resistance 5-8 MΩ) were filled with internal solution consisting of (in mM): K-gluconate (135), KCl (7), HEPES (10), sodium phosphocreatine (10), MgATP (2), Na_2ATP (2), Na_2GTP (0.5), with pH 7.2 and 285-295 mOsm and lowered to the right depth at an angle of 60° using a micromanipulator (Scientifica). Biocytin (1-2 mg/ml) was added before recording. Cell-attached recordings were performed at 250-400 μm from the pial surface using a Multiclamp 700B amplifier (Molecular Devices, USA). The signal was filtered at 10 kHz and acquired at 20 kHz using PClamp 10 software using a DigiData 1440A DAC interface (Molecular Devices, USA). Purkinje cells were identified based on the occurrence of both simple spikes and complex spikes.

Recordings used for analysis lasted from 18 s to 372 s during quiet wakefulness (*HCN1*$^{+/+}$: mean average 166.9 ± 19.5 s, n = 21, in 7 mice; *HCN1*$^{-/-}$: mean average 185.3 ± 22.4 s, n = 17, in 9 mice) and from 5 s to 71 s during movement (*HCN1*$^{+/+}$: mean average 26.6 ± 7.2 s, n = 12, in 6 mice; *HCN1*$^{-/-}$: mean average 20.9 ± 3.5 s, n = 12 , in 6 mice). Each recording contained 10 – 539 complex spikes during quiet wakefulness (*HCN1*$^{+/+}$: mean average 194 ± 26 complex spikes; *HCN1*$^{-/-}$: mean average 135 ± 31 complex spikes) and 7 –95 complex spikes during movement (*HCN1*$^{+/+}$: mean average 38 ± 8 complex spikes; *HCN1*$^{-/-}$: mean average 33 ± 9 complex spikes).

Motion index. We used a motion index to separate periods of quiet wakefulness, in which the animal is not moving but seemingly still alert (not asleep), from periods were the animal was clearly moving. The motion index was calculated as described previously (Jelitai et al., 2016). All movements (positioning, grooming and locomotion) were captured using a digital camera (60 fps) and synchronized with each electrophysiological recording. We calculated the motion index for each successive frame: $MI_f=S^N{}_{i=1}(c_{f+1,i}-c_{f,i})2$, where $c_{f,i}$ is the grayscale level of the pixel i in frame f. Movement was defined as periods where the motion index was above 1 a.u. for at least 2 s. Periods of quiet wakefulness were defined as periods with a motion index below 1 a.u. for at least 2 s. Any periods which were not clearly movement or clearly quiet wakefulness were not analyzed. Visual inspection of each video confirmed the periods of movement (where the mice were either walking or running, but not grooming) and quiet wakefulness.

Immunohistochemistry. Procedures for immunohistochemical labeling of HCN1 channels are as described previously (Rinaldi et al., 2013). Mice were transcardially perfused and brains were left overnight in 4% PFA at 4°C, before being put in 30% sucrose for at least 36 hours at 4 ^{o}C. The caudal parts of the brains containing both cerebellum and brainstem were sliced coronally at a thickness of 40 µm at a freezing microtome (HM 450, Thermo Scientific) and embedded in O.C.T. compound (VWR). Slices were washed for 3×10 min in phosphate-buffered saline (PBS), and blocking was performed in 10 % normal goat serum (NGS) in PBS-0.5 % Triton (PBS-T) for 2 hours at room temperature. Primary polyclonal rabbit antibody against HCN1 (Neuromab) was incubated at a

dilution of 1:1000 in PBS-T with 1% NGS overnight at room temperature. Excess antibody was removed by washing 4×10 min in PBS, and secondary antibody (goat anti-rabbit Alexa 546, A11010, Molecular Probes) was incubated at a dilution of 1:1,000 in PBS-T with 1% NGS for 2 h. Slices were washed for 3×10 min in PBS and a 4,6-diamidino-2-phenylindole (DAPI) staining was performed for 5 min (1:6,000 dilution of staining solution in PBS, original staining solution stock 2 mg/ml, 32670 Sigma, in H_2O). Slices were washed 3×10 min in PBS and embedded with mowiol. Imaging of sections was carried out using a Nikon A1R confocal microscope.

Data analysis and statistical methods. *In vitro* electrophysiological data was analyzed in IGOR pro (Wavemetrics) using Neuromatic (http://www.neuromatic.thinkrandom.com/) and custom-written routines, or using Axograph. *In vivo* electrophysiological data was analyzed using custom-written programs in Python (www.python.org). Simple spikes, complex spikes and their associated spikelets were automatically detected and then visually verified. The reported number of spikelets per complex spike excludes the initial sodium spike component. Complex spike duration was defined as the time between the peak of the first sodium spike of the complex spike to the peak of the last spikelet of the same complex spike. Further statistical analysis was carried out using Python (www.python.org), IGOR pro, Excel (Microsoft), IBM SPSS Statistics version 17.0 (New York, USA), or R (www.R-project.org). Mean values are reported as ± standard error of the mean (SEM). Statistical significance was tested with with linear regression, Student's t test, one-way ANOVA and post hoc Fisher's LSD or Tukey's HSD where appropriate, two-way repeated measures ANOVA, Kolmogorov-Smirnov test, or the Mann-Whitney *U* test.

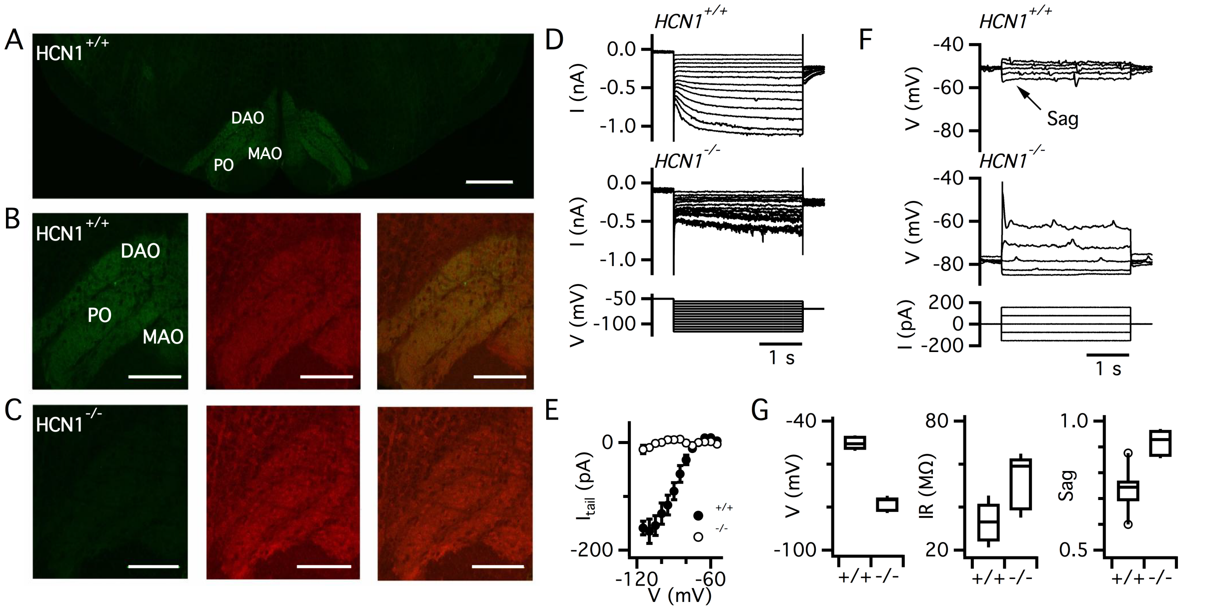

Figure S1. HCN1 channels mediate I_h in IO neurons (relates to Figure 2)

(A) Low magnification image of coronal brain stem section from a *HCN1*$^{+/+}$ mouse labelled with an antibody against HCN1 (green). DAO is dorsal accessory olive, PO is principal olive and MAO is medial accessory olive (scale bar 500 µm).

(B) Higher magnification view of the section in (A) illustrating double labeling with antibodies against HCN1 (left), MAP2 (centre) and merged (right)(scale bar 250 µm).

(C) Images of the IO from *HCN1*$^{-/-}$ mice labelled with antibodies against HCN1 (left), MAP2 (centre) and merged (right) (scale bar 250 µm).

(D) Voltage-clamp recordings of membrane current responses to voltage steps (lower) obtained from IO neurons in slices from *HCN1*$^{+/+}$ mice (upper) and *HCN1*$^{-/-}$ mice (middle).

(E) Plot of mean tail currents measured at -70 mV as a function of test potential. The maximum tail current amplitude for neurons from *HCN1*$^{+/+}$ mice was -172 ± 21 pA (n=9) compared with -12 ± 8 pA (n=9) for neurons from *HCN1*$^{-/-}$ mice ($p=6.3 \times 10^{-4}$, t-test).

(F) Examples of membrane potential responses to current steps (lower) recorded from IO neurons from *HCN1*$^{+/+}$ mice (upper) and *HCN1*$^{-/-}$ mice (middle).

(G) Box plots of modal resting membrane potential ($p = 4.1 \times 10^{-14}$, t-test, n = 9)(left), input resistance ($p = 7.0 \times 10^{-4}$) and sag ($p = 8.8 \times 10^{-6}$).

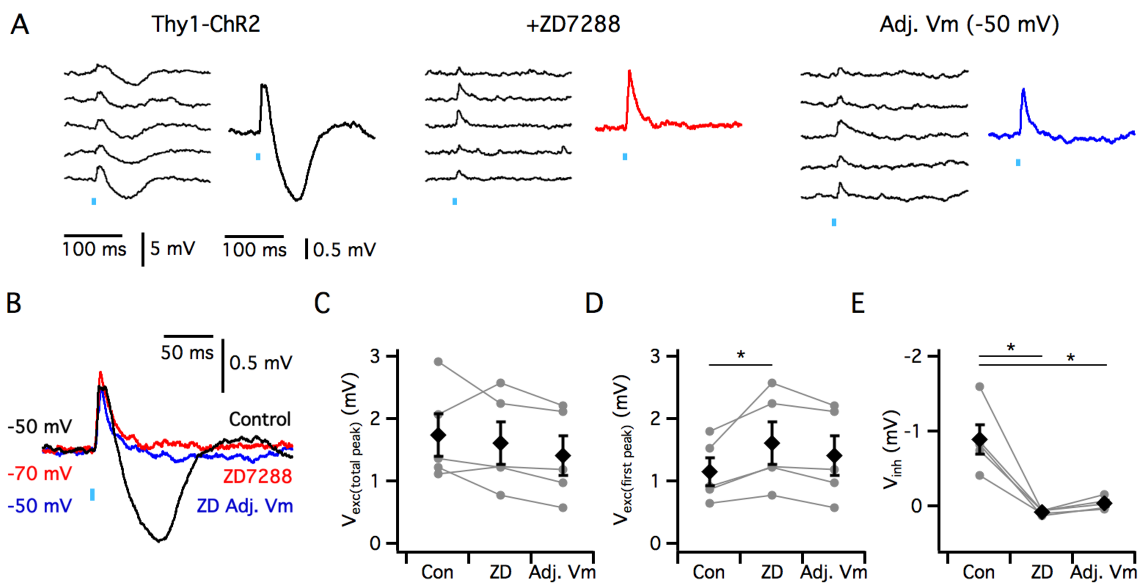

Figure S2. ZD7288-sensitive inhibitory component of PSPs is not rescued by depolarization (relates to Figure 2)

(A) Example responses of Thy1-ChR2 mice to optical activation in control conditions (left), and during application of ZD7288 in the absence of applied current (centre) and during injection of positive current to restore the membrane potential to its control value (right).

(B) Average responses superimposed for each condition in (A).

(C-E) Plots of the amplitude of the depolarizing (C, D) and hyperpolarizing (E) components for each condition. The maximum amplitude of the excitatory component (C) does not depend upon the condition ($F_{2,8}$ = 1.1 p = 0.37 one-way repeated measure ANOVA, n =5). The amplitude of the first peak (D) of the excitatory component depends on condition ($F_{2,8}$ = 8.1 p = 0.01, one-way repeated measure ANOVA), is increased by ZD7288 (Con v ZD p = 0.01, Fisher's LSD) and is restored by depolarization (Con v ZD AdjVm p = 0.22, Fisher's LSD). The amplitude of the inhibitory component (E) depends on condition ($F_{2,8}$ = 26.7 p = 0.007, one-way repeated measure ANOVA) is reduced by ZD7288 (Con vs ZD p = 6.4×10^{-5}, Fisher's LSD) and is not restored by subsequent depolarization (Con v ZD AdjVm p = 0.0002, Fisher's LSD).

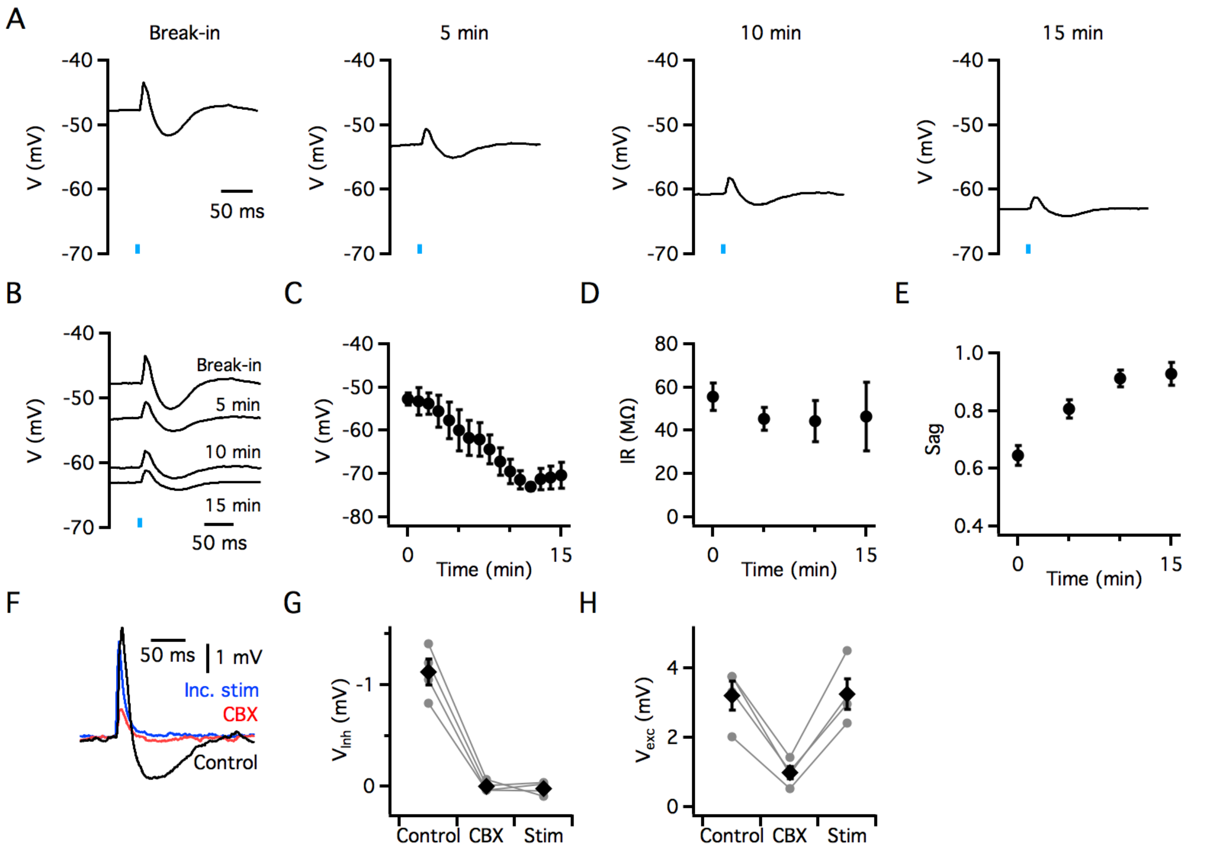

Figure S3. Change in intrinsic membrane properties during intracellular dialysis with ZD7288 (relates to Figure 3)

(A) Examples of light-evoked synaptic potentials recorded from a Thy1-ChR2 mouse at different times after break in.

(B) Data from (A) replotted to illustrate the change in membrane potential during intracellular dialysis with ZD7288.

(C-E) Mean resting membrane potential (C) ($F_{3,12}$ = 17.7, p = 0.0001, one-way repeated measures ANOVA, 0 min v 15 min p = 0.0005, Fisher's LSD, n = 5) , input resistance (D) ($F_{3,12}$ = 1.1, p = 0.40, one-way repeated measures ANOVA, n =5) and membrane potential sag response to current steps (E) ($F_{3,12}$ = 27.7, p = 1.1 x 10^{-5}, one-way repeated measures ANOVA, 0 min v 15 min p = 2.6 x 10^{-6}) plotted as a function of time since break in.

(F) Examples of light-evoked synaptic potentials recorded from a Thy1-ChR2 mouse in control conditions, during perfusion of carbenoxolone and then with the stimulus intensity increased.

(G-H) In the presence of carbenoxolone the amplitude of the depolarizing component of the glutamatergic synaptic response (control 3.5 ± 0.2 mV, carbenoxolone 1.3 ± 0.3, p =

0.0007, n = 4, paired t-test)(G) was reduced and the hyperpolarizing component was completely abolished (p = 0.0008, n = 4, paired t-test)(H). Carbenoxolone was bath applied for 30 minutes, also resulting in a moderate increase in input resistance (control 21.1 ± 4.9 MΩ, carbenoxolone 32.5 ± 9.1 MΩ, p = 0.33, n = 4, paired t-test) without any change in membrane potential (control -53.49 ± 2.06 mV, carbenoxolone -53.64 ± 0.68 mV, p = 0.95, n = 4, paired t-test).

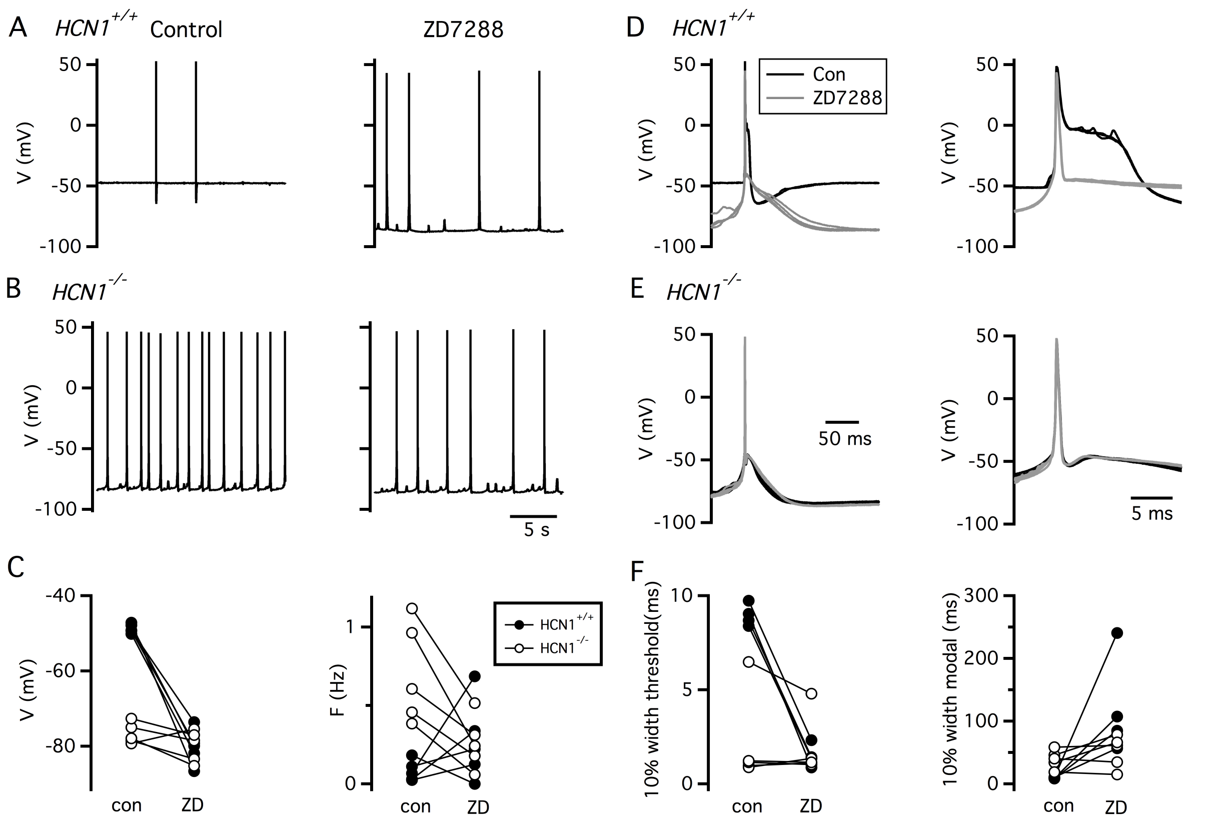

Figure S4. Effects of HCN1 deletion on action potential firing are reproduced by pharmacological block of I_h (relates to Figures 4 and 5)

(A-B) Examples of membrane potential of neurons *HCN1$^{+/+}$* mice (A) and *HCN1$^{-/-}$* mice (B) recorded in control conditions (left) and subsequently during perfusion of ZD7288 (right).

(C) Membrane potential ($F_{1,16}$ = 123.1 p = 6.36 x 10^{-9} for effect of ZD7288, $F_{1,16}$ = 81.6 p = 1.1 x 10^{-7} for interaction between genotype and ZD7288, one way repeated measures ANOVA, *HCN1$^{+/+}$* Con v ZD p = 1.0 x 10^{-7} *HCN1$^{-/-}$* Con v ZD p = 0.48, Tukey's HSD, n = 5), spike frequency ($F_{1,16}$ = 1.3 p = 0.28 for effect of ZD7288, $F_{1,16}$ = 11.6 p = 0.004 for interaction between genotype and ZD7288, one way repeated measures ANOVA, *HCN1$^{+/+}$* Con v ZD p = 0.32 *HCN1$^{-/-}$* Con v ZD p = 0.033, Tukey's HSD, n = 5) and coefficient of variation ($F_{1,16}$ = 8.8 p = 0.01 for effect of ZD7288, $F_{1,16}$ = 7.0 p = 0.019 for interaction between genotype and ZD7288, one way repeated measures ANOVA, *HCN1$^{+/+}$* Con v ZD p = 0.09 *HCN1$^{-/-}$* Con v ZD p = 0.68, Tukey's HSD, n = 5) for all neurons from *HCN1$^{+/+}$* mice (closed circles) and *HCN1$^{-/-}$* mice (open circles) in control conditions (con) and during subsequent perfusion of ZD7288 (ZD)

(D-E) Waveforms of consecutive spontaneous action potentials recorded from *HCN1*$^{+/+}$ (D) and *HCN1*$^{-/-}$ IO neurons (E) recorded in control conditions and during subsequent perfusion of ZD7288. Waveforms to the right show the spikes on a 10x expanded time scale.

(F) Width of the spike (left) ($F_{1,16}$ = 20.7 p = 0.0004 for effect of ZD7288, $F_{1,16}$ = 22.3 p = 0.0003 for interaction between genotype and ZD7288, one way repeated measures ANOVA, *HCN1*$^{+/+}$ Con v ZD p = 6.7 x 10^{-5} *HCN1*$^{-/-}$ Con v ZD p = 0.99, Tukey's HSD, n = 5), and the spike complex (right) ($F_{1,16}$ = 8.6 p = 0.011 for effect of ZD7288, $F_{1,16}$ = 6.8 p = 0.021 for interaction between genotype and ZD7288, one way repeated measures ANOVA, *HCN1*$^{+/+}$ Con v ZD p = 0.008 *HCN1*$^{-/-}$ Con v ZD p = 0.97, Tukey's HSD, n = 5), measured as in Figure 6, for all neurons perfused with ZD7288, labelled as in (C).

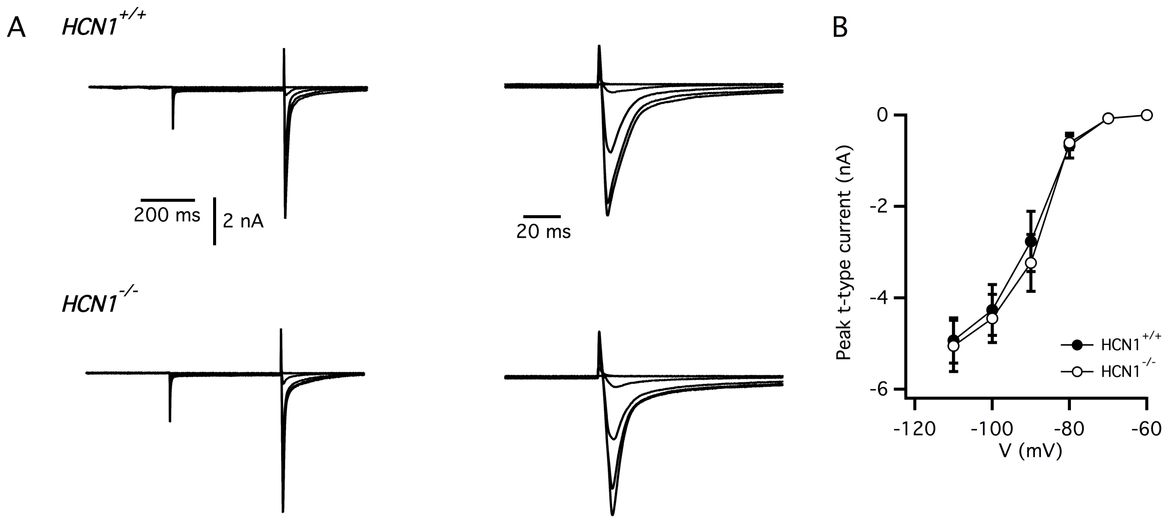

Figure S5. Deletion of HCN1 does not cause adaptation of T-type currents in IO neurons (relates to Figure 4)

(A) Examples of T-type currents recorded from IO neurons from *HCN1+/+* (top) and *HCN1-/-* mice (bottom).

(B) The mean peak amplitude of isolated T-type currents is plotted as a function of membrane potential for IO neurons from *HCN1+/+* and *HCN1-/-* mice. There was no significant difference in the maximal tail current amplitude (p=0.79, n=5, t-test).

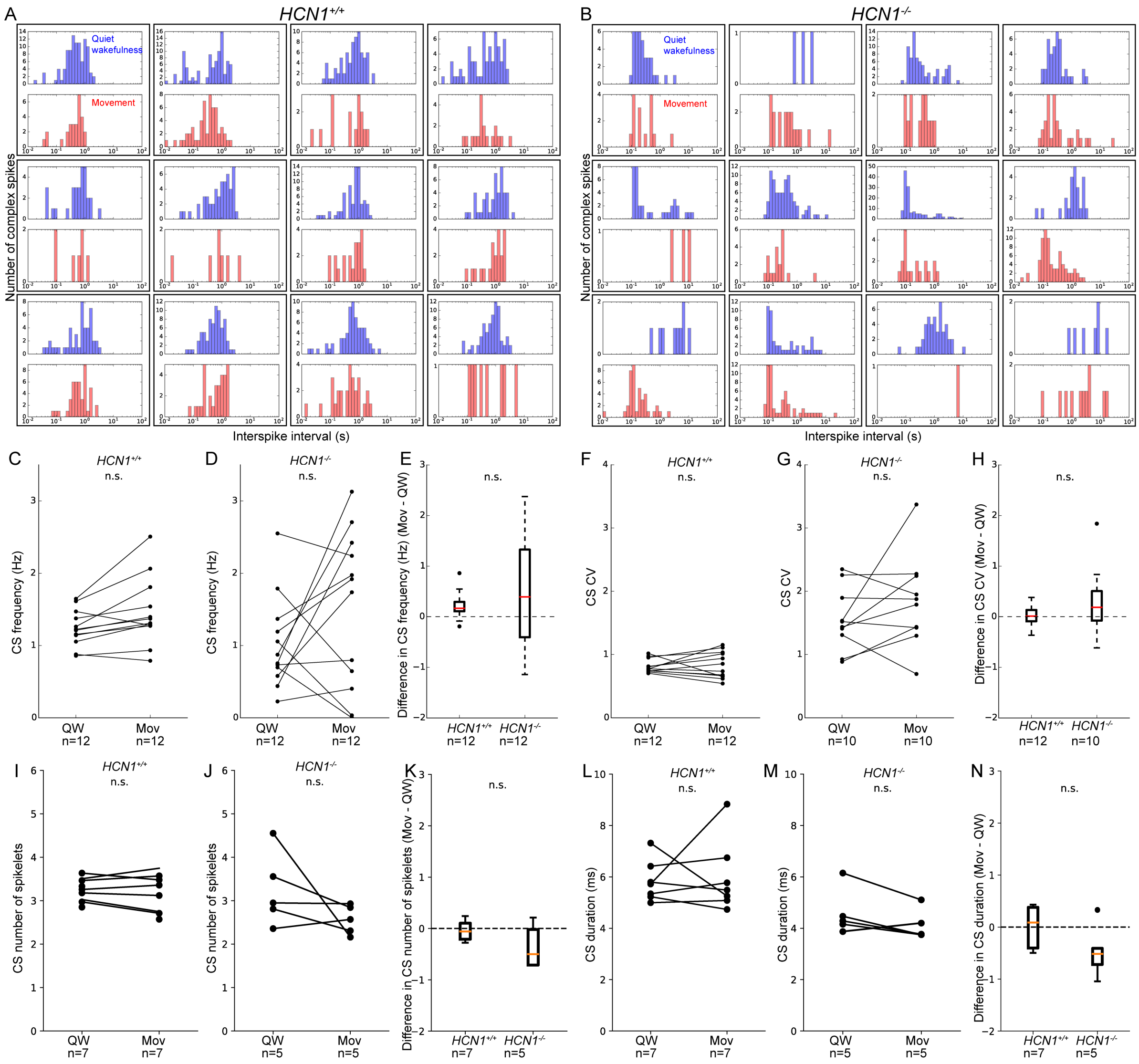

Figure S6. Complex spike properties during quiet wakefulness and movement (relates to Figure 6)

(A - B) Histograms of ISI distributions of individual cells from *HCN1+/+* mice (A) and from *HCN1-/-* mice (B), during quiet wakefulness (blue) and movement (red). Each black box indicates data for one Purkinje cell.

(C - D) Absolute values of complex spike frequency during quiet wakefulness and movement in *HCN1+/+* mice (C) and *HCN1-/-* mice (D).

(E) Difference in complex spike frequency between periods of movement and periods of quiet wakefulness.

(F - G) Absolute values of complex spike CV during quiet wakefulness and movement in *HCN1+/+* mice (F) and *HCN1-/-* mice (G).

(H) Change in complex spike CV during periods of movement when compared to periods of quiet wakefulness.

(I-J) Absolute values of the number of CS spikelets during quiet wakefulness and movement in *HCN1*$^{+/+}$ mice (I) and *HCN1*$^{-/-}$ mice (J).

(K) Difference in the number of CS spikelets between movement and quiet wakefulness.

(L-M) The duration of complex spikes recorded during quiet wakefulness and movement from *HCN1*$^{+/+}$ mice (L) and *HCN1*$^{-/-}$ mice (M).

(N) Difference in the CS duration between movement and quiet wakefulness.

	Quiet wakefulness		Movement	
	HCN1$^{+/+}$	*HCN1*$^{-/-}$	*HCN1*$^{+/+}$	*HCN1*$^{-/-}$
**CS frequency (Hz)**	1.21 ± 0.06 (n=21)	0.87 ± 0.14 (n=17)	1.46 ± 0.14 (n = 12)	1.50 ± 0.31 (n = 12)
**Difference in mean**[a]	*U* = 72, p = 0.002		*U* = 68, p = 0.840	
**Difference in distribution**[b]	*D* = 0.599, p = 0.001		*D* = 0.333, p = 0.433	
**CS CV**	0.79 ± 0.02 (n=21)	1.48 ± 0.13 (n=17)	0.83 ± 0.06 (n = 12)	1.83 ± 0.23 (n = 12)
**Difference in mean**[a]	*U* = 27, p = 0.000		*U* = 7, p = 0.001	
**Difference in distribution**[b]	*D* = 0.7507, p = 0.000		*D* = 0.9000, p = 0.000	
**Number of spikelets**	3.24 ± 0.08 (n=14)	3.37 ± 0.21 (n = 10)	3.20 ± 0.16 (n = 7)	2.56 ± 0.15 (n = 5)
**Difference in mean***	*U* = 61, p = 0.828		*U* = 4, p = 0.034	
**Difference in distribution**[b]	*D* = 0.3571, p = 0.363			
**CS duration (ms)**	5.81 ± 0.20 ms (n = 14)	6.14 ± 0.88 ms (n = 10)	5.99 ± 0.53 (n = 7)	4.11 ± 0.26 (n = 5)
**Difference in mean**[a]	*U* = 59, p = 0.734		*U* = 2, p = 0.014	
**Difference in distribution**[b]	*D* = 0.400, p = 0.237			
**SS frequency (Hz)**	54.50 ± 6.71 (n=21)	49.41 ± 3.72 (n=17)	67.10 ± 7.65 (n = 12)	62.89 ± 7.99 (n = 12)
**Difference in mean**[a]	*U* = 132, p = 0.255		*U* = 64, p = 0.665	
**Difference in distribution**[b]	*D* = 0.252, p = 0.525		*D* = 0.167, p = 0.991	

[a] Mann-Whitney U test
[b] Kolmogorov-Smirnov test

Table S1. Properties of complex spikes and simple spikes recorded from Purkinje cells *in vivo* (relates to Figure 6)

Data recorded from Purkinje cells in awake, behaving mice, comparing *HCN1*$^{+/+}$ and *HCN1*$^{-/-}$ mice during periods of quiet wakefulness and periods of movement.

	HCN1+/+		*HCN1*-/-	
	**Quiet wakefulness**	**Movement**	**Quiet wakefulness**	**Movement**
**CS frequency (Hz)**	1.24 ± 0.07 (n = 12)	1.46 ± 0.14 (n = 12)	1.02 ± 0.19 (n = 12)	1.50 ± 0.31 (n = 12)
**Difference in mean**[a]	*U* = 47, p = 0.158		*U* = 56, p = 0.370	
**Difference in distribution**[b]	*D* = 0.333, p = 0.433		*D* = 0.4167, p = 0.186	
**Difference in CS frequency (Mov – QW)**	0.22 ± 0.08 (n = 12)		0.48 ± 0.34 (n = 10)	
**Difference in mean**[a]	*U* = 65, p = 0.707			
**CS CV**	0.81 ± 0.03 (n = 12)	0.83 ± 0.06 (n = 12)	1.55 ± 0.16 (n = 10)	1.84 ± 0.23 (n = 10)
**Difference in mean**[a]	*U* = 67, p = 0.796		*U* = 40, p = 0.472	
**Difference in distribution**[b]	*D* = 0.4167, p = 0.186		*D* = 0.300, p = 0.675	
**Difference in CS CV (Mov – QW)**	0.019 ± 0.057 (n = 12)		0.28 ± 0.22 (n = 10)	
**Difference in mean**[a]	*U* = 47, p = 0.410			
**CS number of spikelets**	3.24 ± 0.10 (n = 7)	3.20 ± 0.16 (n = 7)	3.24 ± 0.38 (n = 5)	2.56 ± 0.15 (n = 5)
**Difference in mean**[a]	*U* = 24, p = 1.00		*U* = 20, p = 0.144	
**Difference in CS number of spikelets (Mov – QW)**	-0.044 ± 0.076 (n = 7)		-0.68 ± 0.46 (n = 5)	
**Difference in mean**[a]	*U* = 25, p = 0.256			
**CS duration**	5.83 ± 0.30 (n = 7)	5.99 ± 0.53 (n = 7)	4.59 ± 0.40 (n = 5)	4.11 ± 0.26 (n = 5)
**Difference in mean**[a]	*U* = 26, p = 0.90		*U* = 19, p = 0.210	
**Difference in CS duration (Mov – QW)**	0.16 ± 0.59 (n = 7)		-0.47 ± 0.23 (n = 5)	
**Difference in mean**[a]	*U* = 26, p = 0.194			

[a] Mann-Whitney U test
[b] Kolmogorov-Smirnov test

Table S2. Properties of complex spike firing compared between periods of quiet wakefulness and periods of movement in *HCN1*+/+ and *HCN1*-/- mice (relates to Figure 6)

Data recorded from Purkinje cells in awake, behaving mice, comparing periods of quiet wakefulness and periods of movement in *HCN1*$^{+/+}$ and *HCN1*$^{-/-}$ mice. Only cells that had both a period of quiet wakefulness and a period of movement were taken into account. Statistical comparisons correspond to the data shown in Figure S6(C-N).
